# Applying systems biology to biomedical research and health care: a précising definition of systems medicine

**DOI:** 10.1186/s12913-017-2688-z

**Published:** 2017-11-21

**Authors:** Sebastian Schleidgen, Sandra Fernau, Henrike Fleischer, Christoph Schickhardt, Ann-Kristin Oßa, Eva C. Winkler

**Affiliations:** 1 Faculty of Nursing Science, University of Philosophy and Theology Vallendar, Vallendar, Germany; 2 Chair of Systematic Theology II (Ethics), Friedrich-Alexander-University Erlangen-Nürnberg, Erlangen, Germany; 3 Institute for German, European and International Medical Law, Public Health Law and Bioethics (IMGB), Universities of Heidelberg and Mannheim, Mannheim, Germany; 4 National Center for Tumor Diseases (NCT), Program for Ethics and Patient-Oriented Care, Department of Medical Oncology, Heidelberg University Hospital, Heidelberg, Germany

**Keywords:** Bioinformatics, Conceptual vagueness, Data integration, Modeling, Patient participation, Stratification

## Abstract

**Background:**

Systems medicine has become a key word in biomedical research. Although it is often referred to as P4-(predictive, preventive, personalized and participatory)-medicine, it still lacks a clear definition and is open to interpretation. This conceptual lack of clarity complicates the scientific and public discourse on chances, risks and limits of Systems Medicine and may lead to unfounded hopes. Against this background, our goal was to develop a sufficiently precise and widely acceptable definition of Systems Medicine.

**Methods:**

In a first step, PubMed was searched using the keyword “systems medicine”. A data extraction tabloid was developed putting forward a means/ends-division. Full-texts of articles containing Systems Medicine in title or abstract were screened for definitions. Definitions were extracted; their semantic elements were assigned as either means or ends. To reduce complexity of the resulting list, summary categories were developed inductively. In a second step, we applied six criteria for adequate definitions (necessity, non-circularity, non-redundancy, consistency, non-vagueness, and coherence) to these categories to derive a so-called précising definition of Systems Medicine.

**Results:**

We identified 185 articles containing the term Systems Medicine in title or abstract. 67 contained at least one definition of Systems Medicine. In 98 definitions, we found 114 means and 132 ends. From these we derived the précising definition: Systems Medicine is an approach seeking to improve medical research (i.e. the understanding of complex processes occurring in diseases, pathologies and health states as well as innovative approaches to drug discovery) and health care (i.e. prevention, prediction, diagnosis and treatment) through stratification by means of Systems Biology (i.e. data integration, modeling, experimentation and bioinformatics). Our study also revealed the visionary character of Systems Medicine.

**Conclusions:**

Our insights, on the one hand, allow for a realistic identification of actual ethical as well as legal issues arising in the context of Systems Medicine and, in consequence, for a realistic debate of questions concerning its matter and (future) handling. On the other hand, they help avoiding unfounded hopes and unrealistic expectations. This especially holds for goals like improving patient participation which are intensely debated in the context of Systems Medicine, however not implied in the concept.

**Electronic supplementary material:**

The online version of this article (10.1186/s12913-017-2688-z) contains supplementary material, which is available to authorized users.

## Background

Systems Medicine recently became a buzz word in debates on biomedical research and future health care [1, 2]. However, there is no consensus on the term’s meaning. Currently, five main positions seem to dominate the discussion: (a) Systems Medicine is the successor of Personalized Medicine [3, 4]; (b) Systems Medicine is a precursor of Personalized Medicine or P4-Medicine [3, 5]; (c) Systems Medicine is an equivalent term for Precision Medicine [4]; (d) Systems Medicine means the translation of Systems Biology into medical practice [3, 5–7]; and (e) Systems Medicine is an “assemblage of scientific strategies and practices that include bioinformatics approaches to human biology […]; ‘big data’ statistical analysis; and medical informatics tools” [8].

This divergence in understanding is, however, problematic as it complicates sound debates on chances, risks and limits of Systems Medicine. Moreover, it is difficult to identify and solve ethical as well as legal issues that could arise in the context of Systems Medicine. As a consequence, it is impossible to discuss questions of its matter and (future) handling. Finally, the term’s underspecification may lead to unfounded hopes of patients, for instance, regarding the possibilities of Systems Medicine approaches [6, 9]. Against this background, it becomes clear why it is still seen as one of the key challenges to define Systems Medicine [6].

The goal of this paper is to develop a sufficiently precise, formally adequate definition of Systems Medicine. Hurley [10] differentiates, among others, between stipulative, lexical and précising definitions. While a lexical definition captures the way a word is commonly used, a stipulative definition arbitrarily assigns a meaning to a certain expression, whereas a précising definition tries to reduce the vagueness of a term used in practice [11]. Hence, a précising definition is geared to the everyday usage of a term while, at the same time, it aims at standardizing, harmonizing and structuring the different occurring meanings in everyday usage with regard to formal criteria for adequate definitions.

As the term Systems Medicine is already established in everyday usage, however divergently understood, in the following we develop a précising definition. For this purpose, first, the usage of Systems Medicine in the relevant scientific literature was analyzed and, second, harmonized by appealing to formal criteria in order to develop a *formally* adequate definition of Systems Medicine.

## Methods^1^

In order to identify definitions in the relevant scientific publications on Systems Medicine and provide an overall picture of the research field, we followed the approach of a systematic literature review [12, 13]. We therefore searched PubMed using the keyword “systems medicine”. We focused on PubMed as we were interested in definitions of Systems Medicine virulent in the scientific context. We did not include MeSH-terms in the search strategy as the thesaurus of the US National Library of Medicine relates “systems medicine” to the term “system analysis”, which is defined as “[t]he analysis of an activity, procedure, method, technique, or business to determine what must be accomplished and how the necessary operations may best be accomplished”.^2^ Including MeSH-terms would therefore have pre-selected certain articles according to this understanding of systems analysis. Excluding MeSH-terms, on the contrary, allowed us to stay open to alternative understandings of Systems Medicine. We only searched titles and abstracts to identify those articles in which Systems Medicine is the main focus. Moreover, we assumed that those articles were more likely to contain a definition. We did not restrict the date of publication; our last search was performed on December 31, 2015. Furthermore, we included only articles written in English as our goal was to capture the international debate. Subsequently, we checked full-text availability of the articles identified. Where full-texts were not available, we contacted the respective authors.

### Step 1: Description of the areas of application of systems medicine

To describe the areas of application of Systems Medicine, in a first step, all papers fitting our search strategy were assigned to deductively developed main categories based on general presuppositions regarding content alignment of the papers. These main categories are: *1) with reference to disease* and *2) without reference to disease*. This distinction was made in order to evaluate the current state of clinical application of Systems Medicine. Several subcategories of category 1) were developed inductively based on specific findings from the papers to ensure representing the whole spectrum of diseases dealt with in the articles. Due to the inductive approach, the classification of the diseases was mainly based on terms and references used in the articles and does not refer to a specific disease ontology. However, by categorizing the specific diseases we generally distinguish between chronic, non-cancer diseases and hematological and solid cancers as well as various (unspecified) forms of disease in order to examine the areas of application of Systems Medicine. In main category 2), those papers were included which have no reference to any disease or context of disease.

The first categorization showed that papers with as well as without reference to disease can be further specified with regard to their content alignment: they are either research-related or programmatic articles, i.e. both types of papers only partially relate to a specific context of disease. Accordingly, in a second step, all papers were assigned to the inductively developed main categories *A) research context* and *B) programmatic context* based on their content alignment. Category A) includes papers which present own findings or (new) methods in the fields of basic research, clinical research or translational research and/or discuss results of other research projects in these fields. Basic research was defined as research conducted to increase fundamental knowledge and understanding of physical, chemical or functional mechanisms of life processes and diseases, thereby providing the foundation for clinical research. Clinical research was understood as patient-oriented research, conducted with human subjects (or on material of human origin) including inter alia research on mechanisms of human disease, therapeutic interventions, and the development of new technologies as well as epidemiologic and behavioral studies or health services research. Translational research fosters the multidirectional integration of basic research, clinical research, and population-based research applying basic research to human subjects and moving discoveries and knowledge into initial clinical testing. It can be described as mechanism-oriented clinical research that may include laboratory-based research aimed at clarifying mechanisms of disease, developing drugs etc. [14, 15]. Category B) contains papers which focus on the description of general potentials and challenges as well as expectations and future prospects of Systems Medicine. These include visions of a better or more comprehensive disease understanding and management as well as visions of general possibilities for translation and implementation of Systems Medicine in clinical practice.

In a third step, categories 1) and 2) were crossed with categories A) and B), in order to evaluate the current state of research on Systems Medicine in general as well as in specific contexts of disease.

In a final step, the temporal distribution of publications within the categories 1), 2), A) and B) was examined. The aim thereby was to determine possible trends over time within scientific literature regarding Systems Medicine’s areas of application.

### Step 2: Reconstruction of current systems medicine definitions

In order to reconstruct the usage of defining Systems Medicine, two researchers, Christoph Schickhardt and Sebastian Schleidgen, independently screened full-texts of the papers fitting our search strategy for definitions. Included were not only explicit definitions of Systems Medicine, but also text passages containing definition-like descriptions or characterizations of Systems Medicine. In case their assessments differed, discrepancies were discussed and resolved consensually (thereby ensuring inter-coder reliability). Subsequently, as a prerequisite for developing a précising definition of Systems Medicine, a data extraction tabloid was developed which puts forward a means/ends-distinction. This decision is based on the following two assumptions: first, medicine is a field of action, in which *certain* goals or intentions are pursued by applying *certain* means or methods. Against this background, medical applications can be defined by the means they employ to reach *certain* ends [16]. Second, putting forward a means-ends distinction appears to be an adequate way to arrange the multiple semantic elements of Systems Medicine definitions in order to evaluate them regarding their potential for a précising definition of Systems Medicine. Christoph Schickhardt, Sebastian Schleidgen and Henrike Fleischer arranged the definitional elements found in the literature according to the extraction tabloid resulting in a list of ends and means constitutive for the use of the term of Systems Medicine. For instance, from the definition “systems medicine is an emerging discipline that aims to find novel diagnostic markers and therapeutic targets by combining omics with bioinformatics” [17] there were derived the ends “to find novel diagnostic markers” and “to find therapeutic targets” as well as the means of “combining omics with bioinformatics”.

### Step 3: Development of a précising definition of systems medicine

For the purpose of developing a précising definition of Systems Medicine intending to reduce the vagueness of its usage in practice, we referred to six formal criteria for adequate definitions. These criteria are:

1. necessity (a definition must be necessary, i.e. there must not exist any well-established term equivalent with its definiens),^3^
2. non-circularity (a definition must not be circular, i.e. the definiendum must not appear in the definiens),
3. non-redundancy (a definition must not be redundant, i.e. it must not contain any components which are implied by any other of its components),
4. consistency (a definition must not be inconsistent, i.e. it must not include any logical contradictions),
5. non-vagueness (a definition must not be vague, i.e. the definiens’ meaning must be clear), and
6. coherence (a definition must be coherent, i.e. its single semantic elements must reciprocally underpin each other so that their combination makes sense and is plausible) [10, 11, 16, 18].

An adequate précising definition has to satisfy all six criteria, i.e. the *whole definition* (its semantic components and their relations) must satisfy them. This is the kind of definition we aim at developing in the following. In order to develop such a definition, most criteria can be used to assess the *single components* derived in step 2 before evaluating the whole definition, i.e. to exclude the ends and means found in the literature which are not eligible for a précising definition of Systems Medicine. The criterion of non-circularity (2), for instance, can be applied to *single terms* like “systems approach”, which is not eligible as definitional element of Systems Medicine as the definiens must not contain any central terms of the definiendum. Other criteria like consistency (4) or coherency (6), however, can only be applied to *predicative units of meaning*. Thus, the criteria (2)–(6) can be applied to preselect potential components (single terms or predicative units of meaning, respectively) of a précising definition of Systems Medicine.

Accordingly, we applied the criteria to the ends and means of Systems Medicine found in the literature and assigned the remaining ends and means to categories. This does not mean to accept the ends and means identified in this step as ultimately eligible for a précising definition of Systems Medicine. Rather, in view of the criterion of coherence (6), we started an iterative process, compared the ends and means at several stages of our analysis with the goal of establishing a meaningful equilibrium between them and finally approached a précising definition of Systems Medicine.

## Results

### General results

**Fig. 1:**
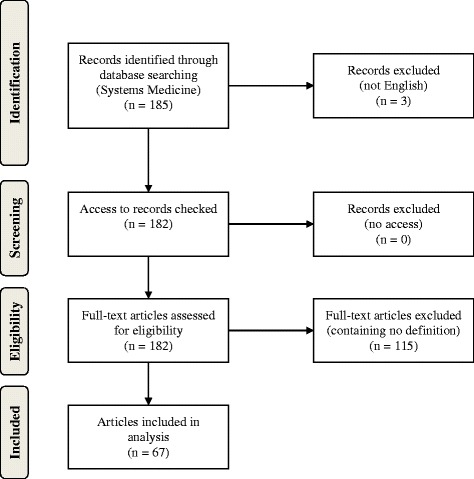
Flow diagram of data collection process according to the PRISMA statement [42]

We identified 185 articles containing the term “systems medicine” in title or abstract. 3 papers were not written in English, but in Chinese, German and Italian, and therefore excluded from further analysis. We screened the remaining 182 articles. 67 contained at least one definition of Systems Medicine and were thus included in steps 2 and 3 of our review (see also Fig. 1).^4^

**Fig. 2:**
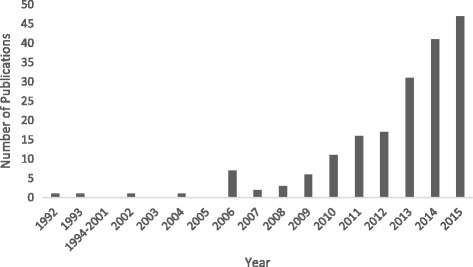
Annual number of papers containing “Systems Medicine” (1992–2015)

In our sample, Systems Medicine was first mentioned in 1992 [19], but the discourse on Systems Medicine did not substantially intensify before 2010/2011. Figure 2 shows the annual number of papers containing the term “systems medicine” in title or abstract in the period of 1992–2015.

The average growth rate of literature written on Systems Medicine was about 41% per year. This is substantially higher than the annual increment of PubMed’s database in the period of 1993–2015, whose average growth rate was 5%.

### Step 1: Description of the areas of application of systems medicine

The reviewed papers (*N* = 182) were assigned to the two main categories *1) with reference to disease* and *2) without reference to specific disease* as follows: 77 papers were assigned to main category 1), 105 papers to main category 2). This frequency distribution shows that 58% of the papers have no reference to specific diseases.

With regard to the specific contexts of disease mentioned in the articles, the literature review reveals that chronic, non-cancer diseases (*n* = 35) as well as hematological and solid cancers (*n* = 33) are the most frequent foci of Systems Medicine (see Table 1). The subcategory chronic, non-cancer diseases includes a variety of diseases. Pulmonary and respiratory diseases (*n* = 12), metabolic and nutritional disorders (*n* = 6), psychiatric and behavioral disorders (*n* = 5) are examined particularly frequently. The third subcategory various types of diseases includes papers not specifying the examined diseases as chronic or non-chronic. It consists of 9 papers. Within this subcategory, immune disorders (*n* = 4) are investigated most frequently. Overall, the frequency distribution of the papers shows that with regard to a single context of disease, Systems Medicine currently is most frequently focused on cancer research. With regard to content alignment, 100 papers were assigned to category *A) research context*, 82 articles to category *B) programmatic context*. Category A) includes 56 papers with reference to disease, 44 papers without reference to specific disease, which means that almost half of the research-related papers do not refer to a specific disease. Furthermore there are some disease-related articles (*n* = 21), which do not present or discuss findings or (new) methods of Systems Medicine and thus are classified as programmatic. Of all papers allocated to category B) programmatic context, 61 papers have no reference to disease. All articles in category B) are formulated in a very general manner meaning they are not related to any specific research methods or findings. Several papers are of a more advertising nature, others have a more basic informative intent.

**Table 1:** Areas of application of Systems Medicine: frequency distribution (*N* = 182)

	A) Research context (*n* = 100)	B) Programmatic context (*n* = 82)
1) With reference to disease (n = 77)	56	21
Cancer, hematological and solid (n = 33)	27	6
Chronic, non-cancer diseases (n = 35)	22	13
- Pulmonary and respiratory diseases (n = 12)	5	7
- Metabolic and nutritional disorders (n = 7)	6	1
- Psychiatric and behavioral disorders (n = 5)	5	/
- Cardiovascular Diseases (n = 4)	1	3
- Neurological diseases (n = 4)	2	2
- Gastrointestinal diseases (n = 3)	3	/
Various types of diseases (n = 9)	7	2
- Immune disorders (n = 4)	4	/
- Influenza (n = 1)	1	/
- Traumatic brain injury (n = 1)	1	/
- Sepsis (n = 1)	/	1
- Allergy (n = 1)	/	1
- Musculoskeletal Diseases (n = 1)	1	/
2) Without reference to specific disease (n = 105)	44	61

Table 1 depicts the frequencies of papers assigned to categories 1) and 2), A) and B) as well as the resulting frequencies from crossing the categories:

The temporal distribution of papers indicates that there are no major scientific changes regarding thematic focus. Focusing on the articles published within the last five years, we found that the number of papers increased in all four categories approximately to the same extent. The same holds for the crossed categories.^5^

### Step 2: Reconstruction of current systems medicine definitions

There seems to be no consensus on the meaning of Systems Medicine. In the 67 papers containing definitions of Systems Medicine or definition-like text passages,^6^ we found 98 definitions with 114 means and 132 ends, depicted in Tables 2 and 3.^7^ We did not carry out a word-by-word analysis to find out the semantic overlap of all definitions and definitions-like text passages found in the literature. This would have been outside our approach which aimed to analyze all definitions and definition-like-text passages by way of building inductive categories and the applying the six formal criteria to the single elements. Furthermore, we did not seek for a consensus definition.

**Table 2:** Means of Systems Medicine in the literature

combines systems biology and pathophysiological approaches to translational research, integrating various bio-medical tools and using the power of computational and mathematical modelling using molecular and dynamic parameters
inferred models
incorporating genomic information (genomic medicine) along with appropriate biological and computational tools for data interpretation
leverages systems biology for clinical application
information and communication technologies, and the conceptual framework of complex system studies
shedding light in multiple research scenarios, ultimately leading to the practical result of uncovering novel dynamic interaction networks that are critical clinical and molecular know-how scrutinizing overall molecular network interactions, rather than individual molecules
an implementation of Systems Biology in the Medical disciplines implies the establishment of a connection between a molecular-centered to a patient-centered world, through an organ-centered intermediate layer. This mapping requires the extensive use of computational tools such as statistical, mathematical and bioinformatical techniques through a shifting paradigm, starting from a cellular, toward a patient centered framework. According to this vision, the three pillars of SM are Biomedical hypotheses, experimental data, mainly achieved by Omics technologies and tailored computational, statistical and modeling tools. The three SM pillars are highly interconnected, and their balancing is crucial is deeply related to complex networks: it involves a systemic view of the organism where the various building elements are considered in their interplay
with all of a patient’s medical data being computationally integrated and accessible to functionally interpret omics and big data incorporating a range of personalized data including genomic, epigenetic, environmental, lifestyle and medical history To achieve these goals, precision medicine aims to develop computational models that integrate data and knowledge from both clinic and basic research to gain a mechanistic understanding of disease
Systems medicine analyzes the dynamic data cloud that surrounds each patient and uses this rely on data as the primary modeling material, not knowledge which purports to design multiscale mathematical disease models
is concerned with the network of molecular interactions that define biological processes. Additionally, disease states are viewed as a perturbation of these molecular networks
amalgamates systems biology techniques with medical treatment decision-making, where information from many biological measurements is combined and analysed for complex patterns of change.
Systems medicine is not simply the application of systems biology in medicine; rather, it is the logical next step and necessary extension of systems biology with more emphasis on clinically relevant applications. Building on the success of systems biology, systems medicine is defined as an emerging discipline that integrates comprehensively computational modeling, ‘omics data, clinical data, and environmental factors utilizes all types of nonlinear information
where traditional model-driven experiments are informed by data-driven models in an iterative manner
molecular fingerprints resulting from biological networks perturbed by the disease will be used the use of network-based models of biological process combined with the information on the patient, mainly of molecular origin integrates physiopathology, network biology and molecular variations through stratification of patients and diseases
data are collected from all the components of the immune system, analyzed and integrated
embraces this paradigm [Systems Biology]
a) taking advantage and emphasizing information and tools made available by the greatest possible spectrum of scientific disciplines b) standardization, information, integration, monitoring and personalization
application of systems biology to medical research and practice
analyzing the interactions between the different components within one organizational level (genome, transcriptome, proteome), and then between the different levels
combining omics with bioinformatics, as well as functional and clinical studies
representing all the available knowledge on the disease of interest with a mathematical symbolism allowing generation and testing of hypotheses through computational simulation and experimental validation
integrate a variety of data at all relevant levels of cellular organisation with clinical and patientreported disease markers, using the power of computational and mathematical modelling
applies the perspective of SB [Systems Biology] to the study of disease mechanisms
a) network-based approach to analysis of high-throughput and routine clinical data to predict disease mechanisms to diagnoses and treatments b) interdisciplinary approach that integrates research data and clinical practice and others view it as fusion of systems biology and bioinformatics with a focus on disease and the clinic c) high-precision, mathematical model of variables from different genomic layers that relate to clinical outcomes such as treatment response
a) interdisciplinary approach that integrates data from basic research and clinical practice b) close integration of data generation with mathematical modeling c) development of concepts, methods and tools that support the integration of organizational levels
a) interdisciplinary effort b) applies the tools and concepts from systems biology and addresses complexity in two key ways. First, systems medicine uses molecular diagnostics to stratify patients and diseases c) applying a network-level view of disease d) identifying important functional and regulatory modules within these networks e) by analyzing and targeting hubs—the most highly interconnected nodes—within these regulatory networks, and enzymatic activity in metabolic networks
a) iterative and reciprocal feedback between data-driven computational and mathematical models as well as model-driven translational and clinical investigations b) specific but large and static data sets acquired across multiple modalities are used
based on theoretical methods and high-throughput “omics” data
a) statistical and computational analysis of metabolic, phenotypic, and physiological data b) application of computational and statistical approaches to support clinical decisions
a) tools for data integration b) sophisticated measurement of molecular moieties
united genomics and genetics through family genomics
different specific complex factors are important in disease management and that these factors need to be incorporated in some meaningful way
standardization of data
integrating experiments in iterative cycles with computational modeling, simulation, and theory
a) identifying all the components of a system, establishing their interactions and assessing their dynamics – both temporal and spatial – as related to their functions b) utilizes all types of biological information – DNA, RNA, protein, metabolites, small molecules, interactions, cells, organs, individuals, social networks and external environmental signals – integrating them
the fully implementation of which requires marrying basic and clinical researches through advanced systems thinking and the employment of high-throughput technologies in genomics, proteomics, nanofluidics, single-cell analysis, and computation strategies in a highly-orchestrated discipline
using the power of computational and mathematical modeling
using knowledge of their molecular components must exploit more limited data sets, arising from multiple open-ended investigations upon highly heterogeneous patient populations in conjunction with vast amounts of poorly correlated published results. Hence, systems medicine must proceed on the basis of existing, highly heterogeneous data and not on the basis of homogeneous datasets arising from specifically targeted investigations.
companion molecular diagnostics for personalized therapy the mounting influx of global quantitative data from both wellness and diseases, which requires new strategies, both scientific and organizational
by determining the links between genotypes, phenotypes and environmental factors (e.g. diet and exposure to toxins) by analysing its different constituents
emphasizes the role of systems biology in medical/clinical applications With the advent of new technologies, the “omics” explosion (i.e., next generation sequencing) and the induced changes from data-poor to data-rich applications (for instance related to high-content imaging, physiology, and structural biology) have established the necessity of a systems approach (Noble, 2008 Systems medicine represents a mosaic of distinct and interconnected micro-systems originated by a variety of information sources and consequently characterized.
leverages complex computational tools and high-dimensional data the effective use of petabytes of data, which necessitates the development of both new types of tools and a new type of physician—one with a grasp of modern computational sciences, “omics” technologies, and a systems approach to the practice of medicine systems biology
This proposed holistic strategy involves comprehensive patient-centered integrated care and multi-scale, multi-modal and multi-level systems approaches Rather than studying each disease individually, it will take into account their intertwined gene-environment, socio-economic interactions and co-morbidities that lead to individual-specific complex phenotypes. based on a robust and extensive knowledge management infrastructure that contains individual patient information. It will be supported by strategic partnerships involving all stakeholders, including general practitioners associated with patient-centered care. This systems medicine strategy, which will take a holistic approach to disease It uses the power of computational and mathematical modeling]. takes a holistic view of health and disease through integrated care using multidisciplinary and teamwork approaches centered in primary and community
Understanding the unique events in an individual’s life as influencing the development of illness and disease appears to be the key to what is emerging under the names of ‘personalized medicine’ and ‘systems medicine’. Personalized medicine presupposes systems biology and complexity sciences, […]
Systems biology and medicine focuses on deciphering mechanisms at multiple levels, reconstructing networks in cells, tissues and organs, measuring and predicting phenotypes, building quantitative models that describe and simulate normal and pathological physiological functions, and then testing the validity of these models and predictions experimentally.
exploration of tumor microenvironment2,15 and of a more global approach to link individual tumors with their multiple host variables,including heritable causal mutations, environmental exposures and lifestyle,
the elucidation of drug targets, an important step in the search for new drugs or novel targets for existing drugs. Incorporating multiple biological information sources is of essence
applicable methodology tool, systems biology.
Systems medicine, the translational science counterpart to basic science’s systems biology, is the interface at which these tools may be constructed […] systems medicine is the coupling of systems science with medical treatment decision-making.
systems medicine approaches focus on the dynamic interactions among multiple factors that affect complex diseases, such as diabetes, coronary artery disease and cancers1. The increasing availability of powerful high-throughput technologies, computational tools and integrated knowledge bases, has made it possible to establish new links between genes, biologic functions and human diseases, providing the hallmarks of systems medicine, including signatures of pathology biology, and links to clinical research and drug discovery. Holistic systems biology methodologies through the construction of integrated biomolecular networks.
The knowledge of network dynamics through in vitro experimental perturbation and modeling allows us to determine the state of the networks, to identify molecular correlates, and. The transformation in biology through systems biology The central premise of systems medicine is that clinically detectable molecular fingerprints resulting from disease-perturbed biological networks will be used to detect and stratify various pathological conditions. Disease associated molecular fingerprints will eventually be used to group individuals into sub-populations based on variations in genetic makeup of the population that affects disease progression. The key to this revolution lies in harnessing the power of network models of core biological processes learned through systems biology methods, combined with vast amounts of diverse molecular information generated from patient samples. depends on our ability to: 1) precisely infer network state from the results of assessing the levels of a panel of informative, diagnostic biomarkers in the blood, and 2) specifically manipulate a network to avoid or revert the pathology. the application of our understanding of the integrated dynamical responses of various molecular networks that determine the critical states of the body. the therapeutic component of systems medicine then, in which we infer network states from biomarker measurements
the application of systems biology
incorporates the complex biochemical, physiological, and environmental interactions that sustain living organisms. incorporates interactions between all components of health and disease. A key feature of systems medicine is that existing networks, through dynamic (time-dependent) interactions, manifest “emergent properties” that define the whole and that these properties are not simply the sum of the features of its component parts.
by integrating all levels of quantitative functional, structural, and morphological information into a coherent model. It investigates the physiological network of diseases from gene to organ systems
via an integrative approach that includes clinical examinations, experimental modeling and in-silico simulation. by integrating all levels of quantitative functional, structural and morphological information into a coherent model.
Systems medicine is an emerging concept that acknowledges the complexity of a multitude of non-linear interactions among molecular and physiological variables. Under this new paradigm, rather than a collection of symptoms, diseases are seen as the product of deviations from a robust steady state compatible with life. the incorporation of mathematics and physics to the more classical arsenal of physiology and molecular biology with which physicians are trained today.

**Table 3:** Ends of Systems Medicine in the literature

enables the personalization of diagnosis, prognosis and treatment helps to re-define clinical phenotypes to discover new diagnostic and prognostic biomarkers to guide the design of new clinical trials
accurately predict sensitivity of an individual tumor to a drug or drug combination to generate genomics informed personalized therapeutic regimes with higher efficacy assist in designing personalized cancer therapy treatments with expected effectiveness significantly higher than current standard of care approaches
to deliver P4 and precision medicine in the future. This will enable introduction of individualized tailored prevention and/or treatment strategies
to understand the critical points of health maintanance and prevent disease development to aid understanding of the nonpulmonary determinants of heterogeneity in the common and debiliating condition of chronic obstructive pulmonary disease (COPD)
identify clinically important molecular targets for diagnostic and therapeutic measures against such a condition influencing the course of medical conditions to produce exquisite datasets that are employed to generate pathway models and treatment and will hopefully directly contribute to stratified medicine en-route to personalized healthcare The application of systems biology for more effective and clinically applicable research outcomes
links disease-associated genes to the phenotypes they produce, a key goal within systems medicine.
a particular attention to clinical applications, including clinical Bioinformatics and the discrimination of pathological states and related morbidities and comorbidities extension of Systems Biology to Clinical-Epidemiological disciplines
identify new patterns in the pathogenesis, diagnosis and prognosis of chronic diseases
to achieve a shift to future healthcare systems with a more proactive and predictive approach to medicine, where the emphasis is on disease prevention rather than the treatment of symptoms. The individualization of treatment for each patient will be at the centre of this approach to facilitate their application [of omics and big data] to healthcare provision the aim is to treat every patient as an individual case inform rational therapy design for each patient thereby facilitating personalized treatment decisions
to derive “actionable possibilities” that can improve wellness or avoid disease for each patient. predictive, preventive, personalized, and participatory medicine developing new diagnostic and therapeutic reagents to terminate a disease trajectory for each individual early, returning them to wellness aims at predicting the course of a disease in a given patient and how far it can be altered by available therapies the prediction of benefit–risk for a single subject, a group, or a population
the application of systems biology to medicine concerned with the complex network interplay of a biological unit and represents injury and illness as a perturbation to the network
aims to offer new approaches for addressing the diagnosis and treatment of major human diseases uniquely, effectively, and with personalized precision to model and predict disease expression (the pathophenome). Systems medicine integrates basic research and clinical practice, and emphasizes translational and clinical research highly comprehensive and integrative aims to offer a powerful set of methodologies to improve our understanding of disease pathogenesis and to design personalized therapies to address the complexity of human diseases
the clinical application of Systems Biology approaches to medicine
to detect and stratify various pathological conditions providing novel insights into the mechanisms of various diseases, such as diabetes and obesity, overcoming the current limitations of disease complexity
a) to generate a mathematical model that describes or predicts the response of the system to individual perturbations b) interdisciplinary approach that systematically describes the complex interactions between all parts of a biological system, with a view to elucidating new biological rules capable of predicting the behavior of the biological system
adaptation and extension of Systems Biology
aimed at improving risk prediction and individual treatment respecting ethical and legal requirements
to find novel diagnostic markers to find novel therapeutic targets
innovative approach to complex diseases understanding and drug discovery
enable the understanding of the mechanisms, prognosis, diagnosis and treatment of disease
improving the diagnostic process, disease management, and outcomes
a) gain a translational understanding of the complex mechanisms underlying common diseases b) to address the problem that a disease is rarely caused by malfunction of one individual gene product, but instead depends on multiple gene products that interact in a complex network c) natural extension of, or is complementary to, current models for clinical decision-making
a) improve our understanding and treatment of diseases b) further development of systems biology and bioinformatics towards applications of clinical relevance c) to derive a mechanistic understanding of pathologies, prophylaxy and support of therapy optimization d) develop interfaces between the computational and mathematical frameworks used in systems medicine
a) integrate molecular, cellular, tissue, organ, and organism levels of function into computational models that facilitate the identification of general principles. Systems medicine adds a disease focus. b) to better characterize and understand disease complexity c) to create disease networks d) overcome current limitations in drug discovery e) network-based approaches will be able to explore the effects of various drugs in mathematical models
a better understanding of cellular and molecular networks as key pathogenic elements of human diseases
a) implementation of Systems Biology approaches in medical concepts, research and practice b) to construct computational models for the dynamic prediction of disease progression or response to treatment at a personal level
application of the systems biology approach to disease-focused or clinically relevant research problems
a) provide a conceptual and theoretical framework b) practical goal is to provide physicians the tools necessary for harnessing the rapid advances in basic biomedical science into their routine clinical arsenal c) to provide the tools to take into account the complexity of the human body and disease in the everyday medical practice
to answer clinical questions
a) clinical decision making is supported b) integrated study of system level metabolic, phenotypic, and physiological changes in response to disease processes or therapies
application of systems biology in a clinical context
not the mere translation of the terminology from computer and life sciences to the medical field
a) dedicated to deciphering the control mechanisms existing within model organisms such as yeast b) Systems models of disease
more readily identify disease genes
treatment selection and delivery
a) application of a systems biology approach in medical research and clinical practice b) to intervene at an early stage to prevent the occurrence and reduce the suffering of the effects of disease, in contrast to chiefly targeting reactive measures only following the occurrence of disease c) embraces and includes programs such as P4 medicine and personalized medicine d) data integration from omics to the clinic
a) extension of systems biology b) carries this approach forward into a disease-oriented era
application of systems biology approaches to medical research and medical practice
application of systems biology to the challenge of human disease
a) a systems approach to health and disease b) to lead to predictive and actionable models for health and disease
predictive, preventive, personalized, and participatory (P4) medicine translational systems medicine
to integrate a variety of biological/medical data on all relevant levels of cellular organization, to enable an understanding of the pathophysiological mechanisms, prognosis, diagnosis and treatment of disease to represent signs and symptoms of diseases in multi-level computational models of cells, tissues, organs, organ systems and even organisms the application of systems biology approaches to medical research and medical practice molecular) systems biology in medicine
to reconstruct organs and organisms to determine clinical behaviours and interventions a holistic approach to medicine (systems medicine), that could benefit patients and society
is shaping up a transformational paradigm in medicine we termed predictive, preventive, personalized, and participatory (P4) medicine to enable bringing this revolution in medicine to patients and to the healthcare system.
The reconstruction of such biological network models, the combination of these models with omics data and their application to specific medical questions are often referred to as systems medicine. a better understanding of the structure and function of the human genome and its associations helps to understand the behaviour of the human body at all levels of organization it offers the prospects of modelling complex diseases, establishing novel diagnostic and therapeutic techniques, identifying new drug targets, developing a system-orientated drug design strategy and eventually achieving effective personalized medicine
not to be caught in the data deluge. allowing to infer the macro-systems dynamics and produce elements of synthesis such as signatures and profiles
an application of systems biology approaches to biomedical problems in the clinical setting, to derive personalized assessments of disease risk more effective individualized diagnosis, prognosis, and treatment options the foundation for a practice of systems medicine in the future that will be predictive, personalized, preventive, and participatory
Systems or ‘P4’ medicine offers a grand vision for achieving better population health. The four Ps - predictive, preventive, personalized and participatory - invoke a patient-centered approach that prioritizes health promotion over disease treatment
to tackle NCDs as a common group of diseases. for predictive, preventive, personalized and participatory (P4) medicine designed to allow the results to be used globally, taking into account the needs and specificities of local economies and health systems. Systems medicine is the application of systems biology to medical research and practice. to integrate a variety of data at all relevant levels of cellular organization with clinical and patient-reported disease markers. to enable understanding of the mechanisms, prognosis, diagnosis and treatment of disease. It involves a transition to predictive, preventive, personalized and participatory (P4) medicine, which is a shift from reactive to prospective medicine that extends far beyond what is usually covered by the term personalized medicine to tackle all components of the complexity of NCDs so as to understand these various phenotypes and hence enable prevention (Box 2), control through health promotion and personalized medicine, and an efficient use of health service resources
The main goal of systems medicine is to provide predictive models of the pathophysiology of complex diseases as well as define healthy states.
Understanding drugs and their modes of action for improving the accuracy of drug target prediction
new strategies capable of integrating all known information about the elements that make up the reality called asthma, thus offering a detailed mapping of its complexity.
[…] systems medicine, as a translationally relevant extension of systems biology
promise to provide the foundation for such prospective medicine
to derive new disease treatment approaches to reverse the pathology or prevent its progress into a more severe state through the manipulation of network states This general approach, including diagnostics and therapeutics, is becoming known as systems medicine. will enable a new medical discipline – systems medicine intervene to halt and reverse the networks progress into an undesired state
to the prevention of, understanding and modulation of, and recovery from developmental disorders and pathologic processes in human health systems medicine emphasizes that the essential purpose and relevance of models is translational, aimed at diagnostic, predictive, and therapeutic applications. systems medicine aims to discover and select the key factors at each level and integrate them into models of translational relevance, which include measurable readouts and clinical predictions.
tries to understand perturbed physiological systems and complex pathologies in their entirety an integrative and systemic approach for the diagnosis, therapy, and prevention of diseases with four main goals — predictive, preventive, personalized, and participative medicine (P4 medicine).
to understand perturbed physiological systems and complex pathologies in their entirety geared towards obtaining clinical impact with both diagnostic and therapeutic end points.

### Step 3: Development of a précising definition of systems medicine

After applying our criteria (2)–(6) for determining the adequacy of definitions (non-circularity, non-redundancy, consistency, non-vagueness, and coherence) to the ends of Systems Medicine found in the literature and excluding all ends not meeting the criteria,^8^ we inductively assigned the remaining ends to the following preliminary categories^9^:

End i)   Improving Participation
End ii)   Improving Prediction
End iii)   Improving Prevention
End iv)   Improving Stratification
End v)   Improving Treatment
End vi)   Improving Diagnostics
End vii)   Modelling
End viii)    Improving Understanding of Disease/Pathologies/Health States
End ix)   Innovative Approach to Drug Discovery
End x)   Finding Novel Therapeutic Targets
End xi)   Discovering New Diagnostic and Prognostic Biomarkers
End xii)   Re-Defining Clinical Phenotypes
End xiii)    Improving Health Care
End xiv)    Achieving better Population Health

Categories Ends ii) (Improving Prediction), iii) (Improving Prevention), v) (Improving Treatment) and vi) (Improving Diagnostics) are classical ends with regard to improving health care. Accordingly, we assigned them to category End xiii) (Improving Health Care). Categories End x) (Finding Novel Therapeutic Targets), xi) (Discovering New Diagnostic and Prognostic Biomarkers) and xii) (Re-Defining Clinical Phenotypes) were subsumed under category End iv) (Improving Stratification). With regard to category End x) (Finding Novel Therapeutic Targets), this decision followed the insight that it is a particular goal of modern efforts of stratification to base therapeutic measures on novel targets, i.e. targets identified through methods of molecular specification [20–22]. A similar point can be made for categories End xi) (Discovering New Diagnostic and Prognostic Biomarkers) and xii) (Re-Defining Clinical Phenotypes): it is a declared goal of activities to improve stratification (for instance through so called Personalized, Individualized or Precision Medicine approaches), to stratify diseases into smaller subgroups by using (new diagnostic and prognostic) biomarkers [16, 23]. Another goal of stratification, which is particularly associated with the molecular approach of Personalized Medicine, is to re-interpret clinical phenotypes, e.g. through a new specification and classification of tumors on a molecular basis [24]. These considerations left us with the following categories of Systems Medicine ends:

-   Improving Participation
-   Improving Stratification
-   Modelling
-   Improving Understanding of Diseases/Pathologies/Health States
-   Innovative Approach to Drug Discovery
-   Improving Health Care
-   Achieving better Population Health.

In a next step, we applied our criteria (2)–(6) (non-circularity, non-redundancy, consistency, non-vagueness, and coherence) to the means found in the literature on Systems Medicine and excluded all means not meeting the criteria.^10^ Subsequently, we inductively derived ten categories from the remaining means^11^:

Means i)    Application of Systems Biology to Medical Research and Practice
Means ii)    Data Integration
Means iii)    Modelling
Means iv)    Networks
Means v)    Bioinformatics/Computer Tools/Computational Analysis
Means vi)    Addressing Complexity
Means vii)    Stratification
Means viii)    Holistic Approach
Means ix)    Understanding of Illnesses/Diseases
Means x)    Experimental Validation/Experimental Examination

A closer look revealed that some of these categories do not depict adequate means of Systems Medicine and thus have to be excluded. First, as regards category Means iv) (Networks), it has to be stated that, although terms like “network-based approach” [25, 26] or “network-level view” [25] might suggest that networks are a distinctive methodological feature of Systems Medicine, most text passages with reference to “network” show an understanding of the term in the sense of “physiological network”: networks are seen as part of healthy as well as ill physiological processes that are to be investigated rather than to be used as a means for medical research and practice. Networks are a structural characteristic of the physiological reality that Systems Medicine seeks to understand. Networks are thus *object* of research efforts and cannot be a means of research at the same time.

Second, regarding category Means vi) (Addressing Complexity), the text passages addressing complexity state that complexity, understood as dynamic interactions between multiple factors, is a fundamental challenge of Systems Medicine calling for a distinctive conceptual orientation. In this understanding, however, addressing complexity cannot be seen as a specific means for Systems Medicine research or practice. We thus learnt that both categories, networks and addressing complexity, are no means but rather indicate a characteristic feature of the physiological reality that Systems Medicine seeks to investigate and understand. Furthermore, for they consist in the dynamic interactions and interdependencies of several factors, networks can be considered as a phenomenon of complexity and therefore subsumed under addressing complexity. We finally subsumed addressing complexity, including networks, under the category Understanding of Illness/Diseases and considered it a specific challenge of Systems Medicine research efforts.

Third, however, regarding category Means ix) (Understanding of Illnesses/Diseases), it is obvious that understanding of illnesses/diseases is *not only a means*. In particular, this applies to medical approaches like Systems Medicine which not only refers to medical practice but also to biomedical research. The extent to which Systems Medicine is a biomedical research orientation, understanding of the researched phenomena, e.g. illnesses or diseases, is a primary objective. The fact that understanding of diseases was already identified as category End d) also indicates that Means ix) (Understanding of Illnesses/Diseases) rather has to be understood as an end of Systems Medicine.

Fourth, taking a look at category Means viii) (Holistic Approach), it is obvious, that “holistic approach” cannot be considered a concrete means of Systems Medicine. Usually it is used as an attribute, assigned to the perspective or, without specification, to the general approach of Systems Medicine, e.g. “a holistic approach to disease”, “a holistic view of health and disease” [27]. The precise meaning of “holistic” cannot be derived from the respective text passages. If it is used in the sense of “everything”, the term is relative in the sense that “everything” is included, which is considered necessary for the respective phenomenon to be investigated. However, in this understanding, a holistic approach is rather an ideal than a specific means of Systems Medicine.

Fifth, like category Means ix) (Understanding of Illnesses/Diseases), category Means vii) (Stratification) was already assigned to Systems Medicine’s ends (category End b)). And there are good reasons for stratification of patients and diseases not being a specific means of Systems Medicine: stratification was, for instance, identified as an essential goal of Personalized Medicine [16]. With regard to Systems Medicine, it also seems plausible to understand stratification as an *intermediate end* indicating *the specific manner to achieve the ultimate overall goal of Systems Medicine,* i.e. *the improvement of healthcare provision*. Therefore, we decided to exclude stratification from Systems Medicine’s means and to treat it as an end.

These considerations left us at the following list of Systems Medicine means categories:

-    Application of Systems Biology to Medical Research and Practice
-    Data Integration
-    Modelling
-    Bioinformatics/Computer Tools/Computational Analysis
-    Experimental Validation/Experimental Examination

When relating categories Means a)-e) to the categories Ends a)-g), we discovered three incoherencies: First, with regard to the category End a) (Participation) we found almost no means outlining how to achieve this end; of the 98 definitions or definition-like text passages only two mention “patient-centered integrated care” [28], one speaks of an “patient-centered approach” [29]. Patient-centered care is widely understood as aiming at patients’ individual needs as well as personal preferences and values in order to actively include them in the process of shared decision-making [30]. However, the text passages referring to patient-centered care consider it as part of the *strategy*, i.e. the means of System Medicine. Stating that participation (as an end) is pursued through patient-centered care (as a means), however, does not make sense: patient-centered care is a particular form of participation and thus not a plausible means to realize participation. Against this background, we conclude that none of the mentioned means addresses the question of *how* to concretely improve patients’ participation or empowerment in comparison to the status quo. Furthermore, it is striking that “participation” as an end in none of the reviewed papers is mentioned sole or in combination with a semantically identical or similar term. It is only referred to as one of the “P”s in “P4-Medicine”. This indicates that “participation” in the context of Systems Medicine is used as an empty phrase, which is presumably why it has not been taken into consideration by which means the end of (improving) participation could be achieved. Therefore, we excluded “participation” as an end of Systems Medicine. It is important, however, that by excluding participation (including patient-centered care) from the ends of Systems Medicine we are not suggesting on a normative level that participation *should not* be pursued by Systems Medicine approaches. The exclusion is solely due to the application of formal criteria to currently existing definitions of Systems Medicine in the literature.

Second, it is striking that End g) (Achieving better Population Health) is mentioned only once [29]. Although there is much academic debate on the term “population health” [31, 32], in our understanding it designates the aim of improving public health, defined as “the art and science of preventing disease, prolonging life and promoting health through the organized efforts of society” [33] and referring to “all organized measures (whether public or private) to prevent disease, promote health, and prolong life among the population as a whole. Its activities aim to provide conditions in which people can be healthy and focus on entire populations, not on individual patients or diseases” [34]. Classical means to improve population or public health are surveillance and assessment of a population’s health and well-being, the identification of health problems and health hazards in a community as well as providing health protection services (regarding, e.g., the environment, food safety, social determinants and economic inequalities) [34]. Among the means analyzed, however, none of such or similar means are mentioned. Against this background, we excluded the end “achieving better population health” due to it being not coherent with the means.

Third, category Means c) (Modelling) was also mentioned as category End c). If, however, the application of Systems Biology to medical research and practice (Means a)) is a means of Systems Medicine, modelling can only be a means, too, for Means b)-e) (Data Integration, Modelling, Bioinformatics/Computer Tools/Computational Analysis and Experimental Validation/Experimental Examination) are strongly connected to system biology. Data integration (category Means b)) means merging different types of data or data gained from different sources. This aggregation of Data is an essential means of Systems Biology: “Modern Systems Biology tackles biocomplexity through a unique approach that integrates biological data […]” [35]. Such data integration, in turn, is strongly connected to (mathematical) modelling (category Means c)) as part of Systems Biology: “systems biology is a methodology that employs mathematical modeling and computational biology tools to integrate and analyze quantitative biological data” [36]. Against this background, we excluded modelling as an end and arrived at the following list of categories of Systems Medicine ends:

End A)   Improving Stratification
End B)   Improving Understanding of Diseases/Pathologies/Health States
End C)   Innovative Approach to Drug Discovery
End D)   Improving Health Care

Ends B) und C) can be assigned to a new category “Medical research”, leaving us at the final list of Systems Medicine Ends:

End I)   Improving Stratification
End II)   Improving Medical Research
End III)   Improving Health Care

Ends II) and III) show that Systems Medicine aims at improving the classical target areas of medicine per se. Against this background, in turn, End I) is plausibly to be understood as an interim goal that indicates the manner in which improvement shall be achieved in the areas of medical research and health care.

Returning to our list of Systems Medicine means categories, both data integration and (mathematical) modelling (Means b) and c)) point towards bioinformatics (category Means d)) as a means of Systems Biology: bioinformatics, defined as “advancing the scientific understanding of living systems through computation” [37], includes a whole arsenal of computational means and methods of system biology such as information management (of omics and clinical data), data analysis and interpretation, design of new omics experiments, quality control and pre-processing of omics data, (statistical) data analysis methods of large and complex omics-based datasets serving the goal of elucidation and analysis of biological networks [38].

Finally, experimental validation and experimental examination (category Means e)) is a necessary supplement of modelling in Systems Biology, as “systems biology requires ‘detail’ and hence accurate experimentation in vivo” [39]. The procedure of combining (mathematical) modelling with in vivo experiments is an established method of system biology tackling “biocomplexity through a unique approach that integrates biological data, mathematical modeling, and experimental verification in iterative feedback cycles” [35].

It follows that Means b)-e) are well-established means of Systems Biology and, thus, have to be subsumed under category Means a). This leaves us with the only Means of Systems Medicine:

Means Application of Systems Biology to Medical Research and Practice.

Our considerations on adequate means and ends can finally be summarized to derive the following précising definition of Systems Medicine:

*Systems Medicine is an approach seeking to improve medical research (*i.e. *the understanding of complex processes occurring in diseases, pathologies and health states as well as innovative approaches to drug discovery) and health care (*i.e. *prevention, prediction, diagnosis and treatment) through stratification by means of Systems Biology (*i.e. *data integration, modeling, experimentation and bioinformatics).*

It is apparent that neither the means, as already established in the context of Systems Biology, nor the ends of Systems Medicine, especially stratification, are for themselves new elements of medical research and practice. Novelty, nevertheless, results from the specific combination of these means and ends. Thereby, our definition meets the first criterion for adequate definitions (necessity). Furthermore, our previous analysis and iterative critical comparison of means and ends shows that criteria 2–6 (non-circularity, non-redundancy, consistency, non-vagueness, and coherence) are also met.

## Discussion

43% of the papers with reference to disease focus on cancer contexts (hematological or solid cancers), 82% of these papers present research results. This suggests that development of Systems Medicine is most advanced in the area of oncology. 45% of the papers on Systems Medicine, however, have a programmatic alignment, which shows that the description of general future prospects, potentials and challenges play an important role in ongoing works on Systems Medicine. Accordingly, a variety of articles are of *general and visionary nature*. This, in combination with the fact that 58% of *all papers* have no reference to a specific disease, implies that Systems Medicine is rather in an early stage of development and not (yet) part of everyday biomedical research and practice. Moreover, almost half of the papers on Systems Medicine *research* do not refer to any diseases. This strengthens the impression of Systems Medicine’s *visionary character*: much research on Systems Medicine seems to be basic research rather than translational or clinical research. Furthermore, we found no evidence for Systems Medicine being part of health care practice. The temporal distribution of publications on Systems Medicine can be interpreted similarly as it indicates no major changes in scientific publishing with regard to thematic focus. This may be due to issues regarding methods as well as feasibility that are direct consequences of the Systems Biology approach [7].

Against this background, it is vital to avoid unrealistic expectations regarding Systems Medicine approaches. This especially holds for goals like improving *patient participation* which are intensely debated in the context of Systems Medicine: Although our analysis showed that improving participation is referred to in the scientific community, we found no respective proposals for adequate means by which this goal could be achieved. Similar to the debates on the meaning of Personalized or Precision Medicine [16], the vision of Systems Medicine – due to its focus on approaches of the natural sciences and bioinformatics – does not focus on wants or preferences of individual patients, but rather on physiological needs.

Our précising definition also shows that the vision of Systems Medicine, first, aims at improving all classical target areas of health care (i.e. prevention, prediction, diagnosis and treatment) through stratification. Second, it aims at improving biomedical research through a specific research orientation, namely understanding of complex processes occurring in health and diseases by means of Systems Biology. It is highly plausible to consider Systems Medicine a comprehensive approach addressing both biomedical research and health care and aiming at advancements in both research and health care. Progress in health care relevant stratification is attainable only through the discovery, understanding and development of new biomarkers as well as new targeted therapies.

Regarding its focus on stratification, Systems Medicine indeed can be understood as some kind of successor of Personalized or Precision Medicine: Systems Medicine addresses the same, or at least similar goals as Personalized or Precision Medicine. It differs, however, regarding its means: while Personalized Medicine focused on utilizing biological information and biomarkers on the molecular level in order to improve stratification of health care [16, 40], Systems Medicine focuses on means of Systems Biology.

Admittedly, this conclusion is not without difficulties, given that Systems Biology, as it is practiced today, is a relatively young discipline differently defined and understood. However, Systems Biology is now fully established in the life sciences community and evolved into a worldwide recognized discipline [35]. As opposed to Systems Medicine there is a general consensus on its key elements amongst the scientific community. Our understanding of Systems Biology is based on the following descriptions reflecting these key elements: Systems Biology is based on the principles of systems theory. Bridging biological sciences, applied mathematics as well as computational and engineering sciences, it is focused on a systemic understanding of biological processes. It employs mathematical modeling and computational biology tools to integrate and analyze quantitative biological data [35, 36].

The frequent statement that Systems Medicine is the application of Systems Biology in medical research and practice thus clearly points in the right direction and is confirmed by our findings – as long as Systems Biology and its typical means are specified. It is important, however, to keep in mind that stratification in the context of Systems Medicine is *not* to be understood as an *end in itself*, but rather as an intermediate goal referring to the ultimate end of improving medical research and health care.

## Conclusions

Our literature review revealed 67 papers containing 98 definitions of Systems Medicine, which mention 114 means and 132 ends of Systems Medicine. By applying six criteria for adequate definitions to these means and ends (non-circularity, non-redundancy, consistency, non-vagueness, and coherence), we derived the following précising definition:

*Systems Medicine is an approach seeking to improve medical research (*i.e. *the understanding of complex processes occurring in diseases, pathologies and health states as well as innovative approaches to drug discovery) and health care (*i.e. *prevention, prediction, diagnosis and treatment) through stratification by means of Systems Biology (*i.e. *data integration, modeling, experimentation and bioinformatics).*

Our study also revealed the visionary character of Systems Medicine: most papers presenting research results are to be located in the context of basic research. These insights allow for a realistic identification of actual ethical as well as legal issues arising in the context of Systems Medicine and, in consequence, for a realistic debate of questions concerning its matter and (future) handling.

It is important that our definition is *not* a “consensus definition”: our literature analysis reveals that there is no consensus on the meaning of “Systems Medicine”. Against this background, our approach of developing a definition of Systems Medicine combines two different approaches of determining the meaning of a term: one being not normative at all by confining itself to reconstructing the meaning(s) attributed to a term by the members of a community when using the term, and one being highly normative by postulating the “right” meaning of a term, independently from how it is used in practice. It has a reconstructive aspect in starting from definitions or definition-like passages as used or proposed by members of the scientific community. On the other hand, it also has a normative aspect when requiring all (elements of) definitions to meet six formal criteria of an adequate definition. The fact that we only worked on what people had stated and that we did not include content elements by our own might be seen as a “democratic” or consensus oriented procedure. However, we excluded all definitional elements which did not meet our six criteria. Hence, consensus was neither a goal nor a criterion.

### Limitations

The definition presented above is to be understood as *formally* adequate, insofar it satisfies our six criteria for adequate definitions. On the other hand, *as regards content*, we do not claim that it is the only correct definition of Systems Medicine.

## Additional files

Additional file 1: Papers identified through research strategy (DOCX 44 kb)

Additional file 2: Annual number of papers with reference to specific disease (DOCX 15 kb)

Additional file 3: Annual number of papers without reference to specific disease (DOCX 15 kb)

Additional file 4: Annual number of papers in research context (DOCX 15 kb)

Additional file 5: Annual number of papers in programmatic context (DOCX 15 kb)

Additional file 6: Annual number of papers in research context with reference to specific disease (DOCX 15 kb)

Additional file 7: Annual number of papers in programmatic context with reference to specific disease (DOCX 15 kb)

Additional file 8: Annual number of papers in research context without reference to specific disease (DOCX 15 kb)

Additional file 9; Annual number of papers in programmatic context without reference to specific disease (DOCX 15 kb)

Additional file 10; Overview of all definitions and definition-like text passages identified (DOCX 32 kb)

Additional file 11; List of all definitions divided into means and ends (DOCX 30 kb)

Additional file 12: Application of the six adequacy criteria to the ends of Systems Medicine (DOCX 32 kb)

Additional file 13: Inductive assigning of remaining ends to preliminary categories (DOCX 30 kb)

Additional file 14: Application of the six adequacy criteria to the means of Systems Medicine (DOCX 30 kb)

Additional file 15: Inductive derived categories from the remaining means (DOCX 37 kb)

## Abbreviation

P4-Medicine: Predictive, Preventive, Personalized and Participatory Medicine

## Footnotes

1 The methods presented here are based on the approach developed in [16]. Differences in methods result from taking into account applicable critiques of the original account [41].

2
https://meshb.nlm.nih.gov/record/ui?ui=D013597.

3 As stated above, medical applications can be defined by the means they employ to reach certain ends. From this it can be inferred that defining a medical term is necessary if either (1) new medical means (or new combinations of medical means) are employed to reach conventional ends, or (2) conventional medical means are employed to reach new ends, or (3) new medical means (or new combinations of medical means) are employed to reach new ends.

4 See Additional file 1 for an overview of all the papers identified.

5 See Additional files 2, 3, 4, 5, 6, 7, 8 and 9.

6 See Additional file 10 for an overview of all definitions and definition-like text passages identified.

7 See Additional file 11 for a list of all definitions separately divided into ends and means.

8 See Additional file 12.

9 See Additional file 13.

10 See Additional file 14.

11 See Additional file 15.
