# Supplementary material for: Applying systems biology to biomedical research and health care: a précising definition of systems medicine
Source: BMC Health Serv Res. 2017 Nov 21;17:761. doi: 10.1186/s12913-017-2688-z (PMC5698952; doi:10.1186/s12913-017-2688-z)
Supplement: Supplementary file 1 — Papers identified through research strategy (DOCX 44 kb) [file 12913_2017_2688_MOESM1_ESM.docx]

**Papers identified through research strategy**

Stand: 11.01.2016

2015 (47):

1: Ghaffari P, Mardinoglu A, Nielsen J. Cancer Metabolism: A Modeling

Perspective. Front Physiol. 2015 Dec 16;6:382. doi: 10.3389/fphys.2015.00382.

eCollection 2015. Review. PubMed PMID: 26733270; PubMed Central PMCID:

PMC4679931.

2: Kulesskiy E, Saarela J, Turunen L, Wennerberg K. Precision Cancer Medicine in

the Acoustic Dispensing Era: Ex Vivo Primary Cell Drug Sensitivity Testing. J Lab

Autom. 2015 Dec 2. pii: 2211068215618869. [Epub ahead of print] PubMed PMID:

26721820.

3: Petta S, Valenti L, Bugianesi E, Targher G, Bellentani S, Bonino F; Special

Interest Group on Personalised Hepatology of the Italian Association for the

Study of the Liver (AISF); Special Interest Group on Personalised Hepatology of

the Italian Association for the Study of the Liver AISF. A "systems medicine"

approach to the study of non-alcoholic fatty liver disease. Dig Liver Dis. 2015

Nov 14. pii: S1590-8658(15)00691-X. doi: 10.1016/j.dld.2015.10.027. [Epub ahead

of print] PubMed PMID: 26698409.

4: Gürsoy UK, Özdemir V. Introduction to Special Issue: Ready to Link Oral Health

to Systems Medicine and Next Generation Biomarkers? OMICS. 2015 Dec 18. [Epub

ahead of print] PubMed PMID: 26684591.

5: Duffy DJ, Krstic A, Halasz M, Schwarzl T, Fey D, Iljin K, Mehta JP, Killick K,

Whilde J, Turriziani B, Haapa-Paananen S, Fey V, Fischer M, Westermann F, Henrich

KO, Bannert S, Higgins DG, Kolch W. Integrative omics reveals MYCN as a global

suppressor of cellular signalling and enables network-based therapeutic target

discovery in neuroblastoma. Oncotarget. 2015 Dec 22;6(41):43182-201. doi:

10.18632/oncotarget.6568. PubMed PMID: 26673823.

6: Gupta A, Bhatnagar S. Vasoregression: A Shared Vascular Pathology Underlying

Macrovascular And Microvascular Pathologies? OMICS. 2015 Dec;19(12):733-53. doi:

10.1089/omi.2015.0128. PubMed PMID: 26669709; PubMed Central PMCID: PMC4684001.

7: Akhtar MT, Mushtaq MY, Verpoorte R, Richardson MK, Choi YH. Zebrafish as a

Model for Systems Medicine R&D: Rethinking the Metabolic Effects of Carrier

Solvents and Culture Buffers Determined by (1)H NMR Metabolomics. OMICS. 2015 Dec

15. [Epub ahead of print] PubMed PMID: 26669610.

8: Haider S, Rahman R, Ghosh S, Pal R. A Copula Based Approach for Design of

Multivariate Random Forests for Drug Sensitivity Prediction. PLoS One. 2015 Dec

10;10(12):e0144490. doi: 10.1371/journal.pone.0144490. eCollection 2015. PubMed

PMID: 26658256; PubMed Central PMCID: PMC4684346.

9: Manchanda R, Jacobs I. Genetic screening for gynecological cancer: where are

we heading? Future Oncol. 2016 Jan;12(2):207-20. doi: 10.2217/fon.15.278. Epub

2015 Dec 7. PubMed PMID: 26638726.

10: Russo P, Del Bufalo A, Fini M. Deep sea as a source of novel-anticancer

drugs: update on discovery and preclinical/clinical evaluation in a systems

medicine perspective. EXCLI J. 2015 Feb 10;14:228-36. doi:

10.17179/excli2015-632. eCollection 2015. Review. PubMed PMID: 26600744; PubMed

Central PMCID: PMC4652633.

11: de Andrés F, Terán S, Bovera M, Fariñas H, Terán E, LLerena A. Multiplex

Phenotyping for Systems Medicine: A One-Point Optimized Practical Sampling

Strategy for Simultaneous Estimation of CYP1A2, CYP2C9, CYP2C19, and CYP2D6

Activities Using a Cocktail Approach. OMICS. 2015 Nov 24. [Epub ahead of print]

PubMed PMID: 26600202.

12: Canonica GW, Bachert C, Hellings P, Ryan D, Valovirta E, Wickman M, De

Beaumont O, Bousquet J. Allergen Immunotherapy (AIT): a prototype of Precision

Medicine. World Allergy Organ J. 2015 Nov 10;8(1):31. doi:

10.1186/s40413-015-0079-7. eCollection 2015. Review. PubMed PMID: 26594303;

PubMed Central PMCID: PMC4640346.

13: Brown SA. Building SuperModels: emerging patient avatars for use in precision

and systems medicine. Front Physiol. 2015 Nov 6;6:318. doi:

10.3389/fphys.2015.00318. eCollection 2015. PubMed PMID: 26594179; PubMed Central

PMCID: PMC4635220.

14: Klauschen F, Heim D, Stenzinger A. Histological tumor typing in the age of

molecular profiling. Pathol Res Pract. 2015 Dec;211(12):897-900. doi:

10.1016/j.prp.2015.08.001. Epub 2015 Sep 5. Review. PubMed PMID: 26589872.

15: Sintim HO, Gürsoy UK. Biofilms as "Connectors" for Oral and Systems Medicine:

A New Opportunity for Biomarkers, Molecular Targets, and Bacterial Eradication.

OMICS. 2015 Nov 19. [Epub ahead of print] PubMed PMID: 26583256.

16: Wu H, Tremaroli V, Bäckhed F. Linking Microbiota to Human Diseases: A Systems

Biology Perspective. Trends Endocrinol Metab. 2015 Dec;26(12):758-70. doi:

10.1016/j.tem.2015.09.011. Epub 2015 Nov 7. Review. PubMed PMID: 26555600.

17: Emmert-Streib F, Dehmer M. Biological networks: the microscope of the

twenty-first century? Front Genet. 2015 Oct 13;6:307. doi:

10.3389/fgene.2015.00307. eCollection 2015. PubMed PMID: 26528327; PubMed Central

PMCID: PMC4602153.

18: Li H, Pouladi N, Achour I, Gardeux V, Li J, Li Q, Zhang HH, Martinez FD,

'Skip' Garcia JG, Lussier YA. eQTL networks unveil enriched mRNA master

integrators downstream of complex disease-associated SNPs. J Biomed Inform. 2015

Dec;58:226-34. doi: 10.1016/j.jbi.2015.10.010. Epub 2015 Oct 30. PubMed PMID:

26524128; PubMed Central PMCID: PMC4684766.

19: Özdemir V. OMICS 2.0: An Accelerator for Global Science, Systems Medicine and

Responsible Innovation. OMICS. 2015 Oct;19(10):579-80. doi:

10.1089/omi.2015.0133. PubMed PMID: 26484977; PubMed Central PMCID: PMC4615774.

20: Kirschner M, Bauch A, Agusti A, Hilke S, Merk S, Pison C, Roldan J, Seidenath

B, Wilken M, Wouters EF, Mewes HW, Heumann K, Maier D. Implementing systems

medicine within healthcare. Genome Med. 2015 Sep 29;7(1):102. doi:

10.1186/s13073-015-0224-5. PubMed PMID: 26419521; PubMed Central PMCID:

PMC4587913.

21: Siest G, Auffray C, Taniguchi N, Ingelman-Sundberg M, Murray H,

Visvikis-Siest S, Ansari M, Marc J, Jacobs P, Meyer U, Van Schaik RH, Müller MM,

Wevers RA, Simmaco M, Kussmann M, Manolopoulos VG, Alizadeh BZ, Beastall G,

Németh G. Systems medicine, personalized health and therapy. Pharmacogenomics.

2015 Sep;16(14):1527-39. doi: 10.2217/pgs.15.103. Epub 2015 Sep 24. PubMed PMID:

26401575.

22: Shaw DE, Sousa AR, Fowler SJ, Fleming LJ, Roberts G, Corfield J, Pandis I,

Bansal AT, Bel EH, Auffray C, Compton CH, Bisgaard H, Bucchioni E, Caruso M,

Chanez P, Dahlén B, Dahlen SE, Dyson K, Frey U, Geiser T, Gerhardsson de Verdier

M, Gibeon D, Guo YK, Hashimoto S, Hedlin G, Jeyasingham E, Hekking PP,

Higenbottam T, Horváth I, Knox AJ, Krug N, Erpenbeck VJ, Larsson LX, Lazarinis N,

Matthews JG, Middelveld R, Montuschi P, Musial J, Myles D, Pahus L, Sandström T,

Seibold W, Singer F, Strandberg K, Vestbo J, Vissing N, von Garnier C, Adcock IM,

Wagers S, Rowe A, Howarth P, Wagener AH, Djukanovic R, Sterk PJ, Chung KF;

U-BIOPRED Study Group. Clinical and inflammatory characteristics of the European

U-BIOPRED adult severe asthma cohort. Eur Respir J. 2015 Nov;46(5):1308-21. doi:

10.1183/13993003.00779-2015. Epub 2015 Sep 10. PubMed PMID: 26357963.

23: Ayers D, Day PJ. Systems Medicine: The Application of Systems Biology

Approaches for Modern Medical Research and Drug Development. Mol Biol Int.

2015;2015:698169. doi: 10.1155/2015/698169. Epub 2015 Aug 18. Review. PubMed

PMID: 26357572; PubMed Central PMCID: PMC4556074.

24: Calimlioglu B, Karagoz K, Sevimoglu T, Kilic E, Gov E, Arga KY.

Tissue-Specific Molecular Biomarker Signatures of Type 2 Diabetes: An Integrative

Analysis of Transcriptomics and Protein-Protein Interaction Data. OMICS. 2015

Sep;19(9):563-73. doi: 10.1089/omi.2015.0088. PubMed PMID: 26348713.

25: Goldman AW, Burmeister Y, Cesnulevicius K, Herbert M, Kane M, Lescheid D,

McCaffrey T, Schultz M, Seilheimer B, Smit A, St Laurent G 3rd, Berman B.

Bioregulatory systems medicine: an innovative approach to integrating the science

of molecular networks, inflammation, and systems biology with the patient's

autoregulatory capacity? Front Physiol. 2015 Aug 19;6:225. doi:

10.3389/fphys.2015.00225. eCollection 2015. PubMed PMID: 26347656; PubMed Central

PMCID: PMC4541032.

26: Cornish AJ, Filippis I, David A, Sternberg MJ. Exploring the cellular basis

of human disease through a large-scale mapping of deleterious genes to cell

types. Genome Med. 2015 Sep 1;7(1):95. doi: 10.1186/s13073-015-0212-9. PubMed

PMID: 26330083; PubMed Central PMCID: PMC4557825.

27: van den Hoogen LL, van Roon JA, Radstake TR, Fritsch-Stork RD, Derksen RH.

Delineating the deranged immune system in the antiphospholipid syndrome.

Autoimmun Rev. 2016 Jan;15(1):50-60. doi: 10.1016/j.autrev.2015.08.011. Epub 2015

Aug 28. Review. PubMed PMID: 26318678.

28: Castellani GC, Menichetti G, Garagnani P, Giulia Bacalini M, Pirazzini C,

Franceschi C, Collino S, Sala C, Remondini D, Giampieri E, Mosca E, Bersanelli M,

Vitali S, Valle IF, Liò P, Milanesi L. Systems medicine of inflammaging. Brief

Bioinform. 2015 Aug 24. pii: bbv062. [Epub ahead of print] PubMed PMID: 26307062.

29: Milic M, Frustaci A, Del Bufalo A, Sánchez-Alarcón J, Valencia-Quintana R,

Russo P, Bonassi S. DNA damage in non-communicable diseases: A clinical and

epidemiological perspective. Mutat Res. 2015 Jun;776:118-27. doi:

10.1016/j.mrfmmm.2014.11.009. Epub 2014 Dec 10. Review. PubMed PMID: 26255943.

30: Duffy DJ. Problems, challenges and promises: perspectives on precision

medicine. Brief Bioinform. 2015 Aug 5. pii: bbv060. [Epub ahead of print] PubMed

PMID: 26249224.

31: Boissel JP, Auffray C, Noble D, Hood L, Boissel FH. Bridging Systems Medicine

and Patient Needs. CPT Pharmacometrics Syst Pharmacol. 2015 Mar;4(3):e00026. doi:

10.1002/psp4.26. Epub 2015 Mar 27. Review. PubMed PMID: 26225243; PubMed Central

PMCID: PMC4394618.

32: Wheelock ÅM, Paulson L, Litton JE; EuPA Biobank Initiative group. The EuPA

Biobank Initiative: Meeting the Future Challenges of Biobanking in Proteomics &

Systems Medicine. J Proteomics. 2015 Jul 24. pii: S1874-3919(15)30067-1. doi:

10.1016/j.jprot.2015.07.014. [Epub ahead of print] PubMed PMID: 26216594.

33: Gupta SK, Jaitly T, Schmitz U, Schuler G, Wolkenhauer O, Vera J. Personalized

cancer immunotherapy using Systems Medicine approaches. Brief Bioinform. 2015 Jul

14. pii: bbv046. [Epub ahead of print] PubMed PMID: 26174229.

34: Cairelli MJ, Fiszman M, Zhang H, Rindflesch TC. Networks of neuroinjury

semantic predications to identify biomarkers for mild traumatic brain injury. J

Biomed Semantics. 2015 May 18;6:25. doi: 10.1186/s13326-015-0022-4. eCollection

2015. PubMed PMID: 25992264; PubMed Central PMCID: PMC4436163.

35: Thompson JP, Coats TJ, Sims MR. Known knowns, known unknowns, and unknown

unknowns: can systems medicine provide a new approach to sepsis? Br J Anaesth.

2015 Jun;114(6):874-7. doi: 10.1093/bja/aev097. Epub 2015 Apr 22. PubMed PMID:

25904640.

36: Wang RS, Maron BA, Loscalzo J. Systems medicine: evolution of systems biology

from bench to bedside. Wiley Interdiscip Rev Syst Biol Med. 2015

Jul-Aug;7(4):141-61. doi: 10.1002/wsbm.1297. Epub 2015 Apr 17. PubMed PMID:

25891169; PubMed Central PMCID: PMC4457580.

37: Tillmann T, Gibson AR, Scott G, Harrison O, Dominiczak A, Hanlon P. Systems

Medicine 2.0: potential benefits of combining electronic health care records with

systems science models. J Med Internet Res. 2015 Mar 23;17(3):e64. doi:

10.2196/jmir.3082. PubMed PMID: 25831125; PubMed Central PMCID: PMC4387294.

38: DGIM Kommission Wissenschaft und Nachwuchsförderung. [Developing internal

medicine into systems medicine: opportunities and challenges]. Dtsch Med

Wochenschr. 2015 Apr;140(7):523-7. doi: 10.1055/s-0041-101102. Epub 2015 Mar 31.

German. PubMed PMID: 25826039. [DEUTSCH]

39: Seaburn DB. Donald A. Bloch, MD: a remembrance. Fam Syst Health. 2015

Mar;33(1):3-4. doi: 10.1037/fsh0000104. PubMed PMID: 25751179.

40: Mauksch LB, Fogarty CT. Reflections. Fam Syst Health. 2015 Mar;33(1):1-2.

doi: 10.1037/fsh0000114. PubMed PMID: 25751178.

41: Wagner A, Cohen N, Kelder T, Amit U, Liebman E, Steinberg DM, Radonjic M,

Ruppin E. Drugs that reverse disease transcriptomic signatures are more effective

in a mouse model of dyslipidemia. Mol Syst Biol. 2015 Mar 3;11(1):791. doi:

10.15252/msb.20145486. PubMed PMID: 25735304; PubMed Central PMCID: PMC4380926.

42: Ben-Hamo R, Efroni S. MicroRNA regulation of molecular pathways as a generic

mechanism and as a core disease phenotype. Oncotarget. 2015 Jan 30;6(3):1594-604.

PubMed PMID: 25593195; PubMed Central PMCID: PMC4359317.

43: Agustí A, Antó JM, Auffray C, Barbé F, Barreiro E, Dorca J, Escarrabill J,

Faner R, Furlong LI, Garcia-Aymerich J, Gea J, Lindmark B, Monsó E, Plaza V,

Puhan MA, Roca J, Ruiz-Manzano J, Sampietro-Colom L, Sanz F, Serrano L, Sharpe J,

Sibila O, Silverman EK, Sterk PJ, Sznajder JI. Personalized respiratory medicine:

exploring the horizon, addressing the issues. Summary of a BRN-AJRCCM workshop

held in Barcelona on June 12, 2014. Am J Respir Crit Care Med. 2015 Feb

15;191(4):391-401. doi: 10.1164/rccm.201410-1935PP. PubMed PMID: 25531178; PubMed

Central PMCID: PMC4351599.

44: Trefois C, Antony PM, Goncalves J, Skupin A, Balling R. Critical transitions

in chronic disease: transferring concepts from ecology to systems medicine. Curr

Opin Biotechnol. 2015 Aug;34:48-55. doi: 10.1016/j.copbio.2014.11.020. Epub 2014

Dec 10. Review. PubMed PMID: 25498477.

45: Caberlotto L, Lauria M. Systems biology meets -omic technologies: novel

approaches to biomarker discovery and companion diagnostic development. Expert

Rev Mol Diagn. 2015 Feb;15(2):255-65. doi: 10.1586/14737159.2015.975214. Epub

2014 Nov 1. Review. PubMed PMID: 25362968.

46: Lahoz-Beneytez J, Schnizler K, Eissing T. A pharma perspective on the systems

medicine and pharmacology of inflammation. Math Biosci. 2015 Feb;260:2-5. doi:

10.1016/j.mbs.2014.07.006. Epub 2014 Jul 21. PubMed PMID: 25057776.

47: Kuiper J, Rothova A, de Boer J, Radstake T. The immunopathogenesis of birdshot

chorioretinopathy; a bird of many feathers. Prog Retin Eye Res. 2015

Jan;44C:99-110. doi: 10.1016/j.preteyeres.2014.11.003. Epub 2014 Nov 26. Review.

PubMed PMID: 25434765.

2014 (41):

48: Agusti A, Antó JM, Auffray C, Barbé F, Barreiro E, Dorca J, Escarrabill J,

Faner R, Furlong LI, Garcia-Aymerich J, Gea J, Lindmark B, Monsó E, Plaza V,

Puhan MA, Roca J, Ruiz-Manzano J, Sampietro-Colom L, Sanz F, Serrano L, Sharpe J,

Sibila O, Silverman EK, Sterk PJ, Sznajder JI. Personalized Respiratory Medicine:

Exploring the Horizon, Addressing the Issues. Am J Respir Crit Care Med. 2014 Dec

22. [Epub ahead of print] PubMed PMID: 25531178.

49: Trefois C, Antony PM, Goncalves J, Skupin A, Balling R. Critical transitions

in chronic disease: transferring concepts from ecology to systems medicine. Curr

Opin Biotechnol. 2014 Dec 9;34C:48-55. doi: 10.1016/j.copbio.2014.11.020. [Epub

ahead of print] Review. PubMed PMID: 25498477.

50: Ben-Hamo R, Efroni S. MicroRNA regulation of molecular pathways as a generic

mechanism and as a core disease phenotype. Oncotarget. 2014 Dec 6. [Epub ahead of

print] PubMed PMID: 25593195.

51: Wolkenhauer O. Pushing limits by embracing complexity. IET Syst Biol. 2014

Dec;8(6):244-250. doi: 10.1049/iet-syb.2014.0031. Review. PubMed PMID: 25478699.

52: Carusi A. Validation and variability: dual challenges on the path from systems

biology to systems medicine. Stud Hist Philos Biol Biomed Sci. 2014 Dec;48 Pt

A:28-37. doi: 10.1016/j.shpsc.2014.08.008. Epub 2014 Sep 28. PubMed PMID:

25262024.

53: Miralles F, Gomez-Cabrero D, Lluch-Ariet M, Tegnér J, Cascante M, Roca J;

Synergy-COPD consortium. Predictive medicine: outcomes, challenges and

opportunities in the Synergy-COPD project. J Transl Med. 2014 Nov 28;12 Suppl

2:S12. doi: 10.1186/1479-5876-12-S2-S12. Epub 2014 Nov 28. PubMed PMID: 25472742;

PubMed Central PMCID: PMC4255885.

54: Cascante M, de Atauri P, Gomez-Cabrero D, Wagner P, Centelles JJ, Marin S,

Cano I, Velickovski F, Marin de Mas I, Maier D, Roca J, Sabatier P. Workforce

preparation: the Biohealth computing model for Master and PhD students. J Transl

Med. 2014 Nov 28;12 Suppl 2:S11. doi: 10.1186/1479-5876-12-S2-S11. Epub 2014 Nov

28. PubMed PMID: 25472654; PubMed Central PMCID: PMC4255883.

55: Cano I, Lluch-Ariet M, Gomez-Cabrero D, Maier D, Kalko S, Cascante M, Tegnér

J, Miralles F, Herrera D, Roca J; Synergy-COPD consortium. Biomedical research in

a Digital Health Framework. J Transl Med. 2014 Nov 28;12 Suppl 2:S10. doi:

10.1186/1479-5876-12-S2-S10. Epub 2014 Nov 28. PubMed PMID: 25472554; PubMed

Central PMCID: PMC4255881.

56: Gomez-Cabrero D, Menche J, Cano I, Abugessaisa I, Huertas-Migueláñez M, Tenyi

A, de Mas I, Kiani NA, Marabita F, Falciani F, Burrowes K, Maier D, Wagner P,

Selivanov V, Cascante M, Roca J, Barabási AL, Tegnér J. Systems Medicine: from

molecular features and models to the clinic in COPD. J Transl Med. 2014 Nov 28;12

Suppl 2:S4. doi: 10.1186/1479-5876-12-S2-S4. Epub 2014 Nov 28. PubMed PMID:

25471042; PubMed Central PMCID: PMC4255907.

57: Montecucco F, Carbone F, Dini FL, Fiuza M, Pinto FJ, Martelli A, Palombo D,

Sambuceti G, Mach F, De Caterina R. Implementation strategies of Systems Medicine

in clinical research and home care for cardiovascular disease patients. Eur J

Intern Med. 2014 Nov;25(9):785-94. doi: 10.1016/j.ejim.2014.09.015. Epub 2014 Oct

3. PubMed PMID: 25283057.

58: Elo LL, Karjalainen R, Ohman T, Hintsanen P, Nyman TA, Heckman CA,

Aittokallio T. Statistical detection of quantitative protein biomarkers provides

insights into signaling networks deregulated in acute myeloid leukemia.

Proteomics. 2014 Nov;14(21-22):2443-53. doi: 10.1002/pmic.201300460. Epub 2014

Oct 15. PubMed PMID: 25211154.

59: Vogt H, Ulvestad E, Eriksen TE, Getz L. Getting personal: can systems

medicine integrate scientific and humanistic conceptions of the patient? J Eval

Clin Pract. 2014 Oct 14. doi: 10.1111/jep.12251. [Epub ahead of print] PubMed

PMID: 25312489.

60: Cardinal-Fernández P, Nin N, Ruíz-Cabello J, Lorente JA. Systems medicine: a

new approach to clinical practice. Arch Bronconeumol. 2014 Oct;50(10):444-51.

doi: 10.1016/j.arbres.2013.10.010. Epub 2014 Jan 5. English, Spanish. PubMed

PMID: 24397963.

61: Zhang H, Gustafsson M, Nestor C, Chung KF, Benson M. Targeted omics and

systems medicine: personalising care. Lancet Respir Med. 2014 Oct;2(10):785-7.

doi: 10.1016/S2213-2600(14)70188-2. Epub 2014 Sep 16. PubMed PMID: 25239796.

62: Rappaport N, Twik M, Nativ N, Stelzer G, Bahir I, Stein TI, Safran M, Lancet

D. MalaCards: A Comprehensive Automatically-Mined Database of Human Diseases.

Curr Protoc Bioinformatics. 2014 Sep 8;47:1.24.1-1.24.19. doi:

10.1002/0471250953.bi0124s47. PubMed PMID: 25199789.

63: van Ommen B, van der Greef J, Ordovas JM, Daniel H. Phenotypic flexibility as

key factor in the human nutrition and health relationship. Genes Nutr. 2014

Sep;9(5):423. doi: 10.1007/s12263-014-0423-5. Epub 2014 Aug 9. PubMed PMID:

25106484; PubMed Central PMCID: PMC4172643.

64: Levin N. Multivariate statistics and the enactment of metabolic complexity.

Soc Stud Sci. 2014 Aug;44(4):555-78. PubMed PMID: 25272612.

65: Lahoz-Beneytez J, Schnizler K, Eissing T. A pharma perspective on the systems

medicine and pharmacology of inflammation. Math Biosci. 2014 Jul 21. pii:

S0025-5564(14)00139-4. doi: 10.1016/j.mbs.2014.07.006. [Epub ahead of print]

PubMed PMID: 25057776.

66: Zhou X, Menche J, Barabási AL, Sharma A. Human symptoms-disease network. Nat

Commun. 2014 Jun 26;5:4212. doi: 10.1038/ncomms5212. PubMed PMID: 24967666.

67: Roncada P, Modesti A, Timperio AM, Bini L, Castagnola M, Fasano M, Urbani A.

One medicine--one health--one biology and many proteins: proteomics on the verge

of the One Health approach. Mol Biosyst. 2014 Jun;10(6):1226-7. doi:

10.1039/c4mb90011a. Epub 2014 Apr 29. PubMed PMID: 24777557.

68: Würstle ML, Zink E, Prehn JH, Rehm M. From computational modelling of the

intrinsic apoptosis pathway to a systems-based analysis of chemotherapy

resistance: achievements, perspectives and challenges in systems medicine. Cell

Death Dis. 2014 May 29;5:e1258. doi: 10.1038/cddis.2014.36. PubMed PMID:

24874730; PubMed Central PMCID: PMC4047923.

69: Pattini L, Sassi R, Cerutti S. Dissecting heart failure through the

multiscale approach of systems medicine. IEEE Trans Biomed Eng. 2014

May;61(5):1593-603. doi: 10.1109/TBME.2014.2307758. PubMed PMID: 24759286.

70: Kinross J, Li JV, Muirhead LJ, Nicholson J. Nutritional modulation of the

metabonome: applications of metabolic phenotyping in translational nutritional

research. Curr Opin Gastroenterol. 2014 Mar;30(2):196-207. doi:

10.1097/MOG.0000000000000036. Review. PubMed PMID: 24468802.

71: Pison C, Magnan A, Botturi K, Sève M, Brouard S, Marsland BJ, Ernst F,

Paprotka T, Deplanche K, Fritz A, Siroux V, Boissel JP, Corris PA, Auffray C,

Nicod LP; SysCLAD consortium. Prediction of chronic lung allograft dysfunction: a

systems medicine challenge. Eur Respir J. 2014 Mar;43(3):689-93. doi:

10.1183/09031936.00161313. PubMed PMID: 24585866.

72: Kim N, He N, Yoon S. Cell line modeling for systems medicine in cancers

(review). Int J Oncol. 2014 Feb;44(2):371-6. doi: 10.3892/ijo.2013.2202. Epub

2013 Dec 2. Review. PubMed PMID: 24297677; PubMed Central PMCID: PMC3898721.

73: Emmert-Streib F, Zhang SD, Hamilton P. Dry computational approaches for wet

medical problems. J Transl Med. 2014 Jan 25;12:26. doi: 10.1186/1479-5876-12-26.

PubMed PMID: 24460894; PubMed Central PMCID: PMC3905162.

74: Qasba P. A systems approach to blood disorders. Adv Exp Med Biol.

2014;844:395-9. doi: 10.1007/978-1-4939-2095-2_19. PubMed PMID: 25480652.

75: McDermott JE, Huang Y, Zhang B, Xu H, Zhao Z. Integrative genomics and

computational systems medicine. Biomed Res Int. 2014;2014:945253. doi:

10.1155/2014/945253. Epub 2014 Jun 15. PubMed PMID: 25025078; PubMed Central

PMCID: PMC4082850.

76: Russo P, Del Bufalo A, Milic M, Salinaro G, Fini M, Cesario A. Cholinergic

receptors as target for cancer therapy in a systems medicine perspective. Curr

Mol Med. 2014;14(9):1126-38. PubMed PMID: 25324001.

77: Bousquet J, Jorgensen C, Dauzat M, Cesario A, Camuzat T, Bourret R, Best N,

Anto JM, Abecassis F, Aubas P, Avignon A, Badin M, Bedbrook A, Blain H, Bourdin

A, Bringer J, Camu W, Cayla G, Costa DJ, Courtet P, Cristol JP, Demoly P, de la

Coussaye JE, Fesler P, Gouzi F, Gris JC, Guillot B, Hayot M, Jeandel C, Jonquet

O, Journot L, Lehmann S, Mathieu G, Morel J, Ninot G, Pelissier J, Picot MC,

Radier-Pontal F, Robine JM, Rodier M, Roubille F, Sultan A, Wojtusciszyn A,

Auffray C, Balling R, Barbara C, Cambon-Thomsen A, Chavannes NH, Chuchalin A,

Crooks G, Dedeu A, Fabbri LM, Garcia-Aymerich J, Hajjam J, Melo Gomes E, Palkonen

S, Piette F, Pison C, Price D, Samolinski B, Schunemann HJ, Sterk PJ, Yiallouros

P, Roca J, Van de Perre P, Mercier J. Systems medicine approaches for the

definition of complex phenotypes in chronic diseases and ageing. From concept to

implementation and policies. Curr Pharm Des. 2014;20(38):5928-44. PubMed PMID:

24641234.

78: Cesario A, Auffray C, Agusti A, Apolone G, Balling R, Barbanti P, Bellia A,

Boccia S, Bousquet J, Cardaci V, Cazzola M, Dall'Armi V, Daraselia N, Ros LD,

Bufalo AD, Ducci G, Ferri L, Fini M, Fossati C, Gensini G, Granone PM, Kinross J,

Lauro D, Cascio GL, Lococo F, Lococo A, Maier D, Marcus F, Margaritora S, Marra

C, Minati G, Neri M, Pasqua F, Pison C, Pristipino C, Roca J, Rosano G, Rossini

PM, Russo P, Salinaro G, Shenhar S, Soreq H, Sterk PJ, Stocchi F, Torti M,

Volterrani M, Wouters EF, Frustaci A, Bonassi S. A systems medicine clinical

platform for understanding and managing non- communicable diseases. Curr Pharm

Des. 2014;20(38):5945-56. PubMed PMID: 24641232.

79: Cesario A, Auffray C, Russo P, Hood L. P4 medicine needs P4 education. Curr

Pharm Des. 2014;20(38):6071-2. PubMed PMID: 24641231.

80: Ndiaye NC. Systems Medicine in the era of 'Big Data': a game-changer for

Personalized Medicine? Drug Metabol Drug Interact. 2014;29(3):127. doi:

10.1515/dmdi-2014-0022. PubMed PMID: 24995404.

81: Siest G. Systems medicine, stratified medicine, personalized medicine but not

precision medicine. Drug Metabol Drug Interact. 2014;29(1):1-2. doi:

10.1515/dmdi-2013-0068. PubMed PMID: 24468611.

82: Henney A, Coaker H. The Virtual Liver Network: systems understanding from

bench to bedside. Future Med Chem. 2014;6(16):1735-40. PubMed PMID: 25574529.

83: Gustafsson M, Nestor CE, Zhang H, Barabási AL, Baranzini S, Brunak S, Chung

KF, Federoff HJ, Gavin AC, Meehan RR, Picotti P, Pujana MÀ, Rajewsky N, Smith KG,

Sterk PJ, Villoslada P, Benson M. Modules, networks and systems medicine for

understanding disease and aiding diagnosis. Genome Med. 2014 Oct 17;6(10):82.

doi: 10.1186/s13073-014-0082-6. eCollection 2014. PubMed PMID: 25473422; PubMed

Central PMCID: PMC4254417.

84: Wolkenhauer O, Auffray C, Brass O, Clairambault J, Deutsch A, Drasdo D,

Gervasio F, Preziosi L, Maini P, Marciniak-Czochra A, Kossow C, Kuepfer L,

Rateitschak K, Ramis-Conde I, Ribba B, Schuppert A, Smallwood R, Stamatakos G,

Winter F, Byrne H. Enabling multiscale modeling in systems medicine. Genome Med.

2014 Mar 21;6(3):21. doi: 10.1186/gm538. eCollection 2014. PubMed PMID: 25031615;

PubMed Central PMCID: PMC4062045.

85: Brunetto MR, Bonino F. Interferon therapy of chronic hepatitis B.

Intervirology. 2014;57(3-4):163-70. doi: 10.1159/000360941. Epub 2014 Jul 15.

PubMed PMID: 25034484.

86: Dammann O, Gray P, Gressens P, Wolkenhauer O, Leviton A. Systems

Epidemiology: What's in a Name? Online J Public Health Inform. 2014 Dec

15;6(3):e198. doi: 10.5210/ojphi.v6i3.5571. eCollection 2014. PubMed PMID:

25598870.

87: Front matter. Stud Health Technol Inform. 2014;200:i-xi. PubMed PMID:

24851992.

88: Vanfleteren LE, Kocks JW, Stone IS, Breyer-Kohansal R, Greulich T, Lacedonia

D, Buhl R, Fabbri LM, Pavord ID, Barnes N, Wouters EF, Agusti A. Moving from the

Oslerian paradigm to the post-genomic era: are asthma and COPD outdated terms?

Thorax. 2014 Jan;69(1):72-9. doi: 10.1136/thoraxjnl-2013-203602. Epub 2013 Jun

22. Review. PubMed PMID: 23794191.

2013 (31):

89: Pemovska T, Kontro M, Yadav B, Edgren H, Eldfors S, Szwajda A, Almusa H,

Bespalov MM, Ellonen P, Elonen E, Gjertsen BT, Karjalainen R, Kulesskiy E,

Lagström S, Lehto A, Lepistö M, Lundán T, Majumder MM, Marti JM, Mattila P,

Murumägi A, Mustjoki S, Palva A, Parsons A, Pirttinen T, Rämet ME, Suvela M,

Turunen L, Västrik I, Wolf M, Knowles J, Aittokallio T, Heckman CA, Porkka K,

Kallioniemi O, Wennerberg K. Individualized systems medicine strategy to tailor

treatments for patients with chemorefractory acute myeloid leukemia. Cancer

Discov. 2013 Dec;3(12):1416-29. doi: 10.1158/2159-8290.CD-13-0350. Epub 2013 Sep

20. PubMed PMID: 24056683.

90: Kolch W, Kholodenko BN. Systems medicine: opportunities and challenges for

systems biology approaches. FEBS J. 2013 Dec;280(23):5937. doi:

10.1111/febs.12560. Epub 2013 Oct 30. PubMed PMID: 24128262.

91: Caie PD, Schuur K, Oniscu A, Mullen P, Reynolds PA, Harrison DJ. Human tissue

in systems medicine. FEBS J. 2013 Dec;280(23):5949-56. doi: 10.1111/febs.12550.

Epub 2013 Oct 29. Review. PubMed PMID: 24118991.

92: Wolkenhauer O, Green S. The search for organizing principles as a cure

against reductionism in systems medicine. FEBS J. 2013 Dec;280(23):5938-48. doi:

10.1111/febs.12311. Epub 2013 May 24. Review. PubMed PMID: 23621685.

93: Siest G, Ndiaye NC, El Shamieh S, Shahabi P, Stathopoulou M, Saleh AS, Godjo

T, Albertini L, Visvikis-Siest S. Conference Scene: Systems biology and

personalized health science and translation. Pharmacogenomics. 2013

Dec;14(16):1953-64. doi: 10.2217/pgs.13.201. PubMed PMID: 24279850.

94: Spanagel R, Durstewitz D, Hansson A, Heinz A, Kiefer F, Köhr G, Matthäus F,

Nöthen MM, Noori HR, Obermayer K, Rietschel M, Schloss P, Scholz H, Schumann G,

Smolka M, Sommer W, Vengeliene V, Walter H, Wurst W, Zimmermann US; Addiction

GWAS Resource Group, Stringer S, Smits Y, Derks EM. A systems medicine research

approach for studying alcohol addiction. Addict Biol. 2013 Nov;18(6):883-96. doi:

10.1111/adb.12109. PubMed PMID: 24283978.

95: Zhang A, Sun H, Xu H, Qiu S, Wang X. Cell metabolomics. OMICS. 2013

Oct;17(10):495-501. doi: 10.1089/omi.2012.0090. Epub 2013 Aug 29. Review. PubMed

PMID: 23988149; PubMed Central PMCID: PMC3783970.

96: Vandamme D, Fitzmaurice W, Kholodenko B, Kolch W. Systems medicine: helping

us understand the complexity of disease. QJM. 2013 Oct;106(10):891-5. doi:

10.1093/qjmed/hct163. Epub 2013 Jul 30. Review. PubMed PMID: 23904523.

97: Wheelock CE, Goss VM, Balgoma D, Nicholas B, Brandsma J, Skipp PJ, Snowden S,

Burg D, D'Amico A, Horvath I, Chaiboonchoe A, Ahmed H, Ballereau S, Rossios C,

Chung KF, Montuschi P, Fowler SJ, Adcock IM, Postle AD, Dahlén SE, Rowe A, Sterk

PJ, Auffray C, Djukanovic R; U-BIOPRED Study Group. Application of 'omics

technologies to biomarker discovery in inflammatory lung diseases. Eur Respir J.

2013 Sep;42(3):802-25. doi: 10.1183/09031936.00078812. Epub 2013 Feb 8. Review.

PubMed PMID: 23397306.

98: Pey J, Tobalina L, de Cisneros JP, Planes FJ. A network-based approach for

predicting key enzymes explaining metabolite abundance alterations in a disease

phenotype. BMC Syst Biol. 2013 Jul 19;7:62. doi: 10.1186/1752-0509-7-62. PubMed

PMID: 23870038; PubMed Central PMCID: PMC3733687.

99: Robinette SL, Lindon JC, Nicholson JK. Statistical spectroscopic tools for

biomarker discovery and systems medicine. Anal Chem. 2013 Jun 4;85(11):5297-303.

doi: 10.1021/ac4007254. Epub 2013 May 23. PubMed PMID: 23614579.

100: Misgeld T, Lichtenthaler SF, Dichgans M. Between new genetic discoveries and

large randomized trials--neurological research in the era of systems medicine.

EMBO Rep. 2013 Jun;14(6):489-92. doi: 10.1038/embor.2013.70. Epub 2013 May 14.

PubMed PMID: 23670197; PubMed Central PMCID: PMC3674454.

101: Rehm M, Prehn JH. Systems modelling methodology for the analysis of apoptosis

signal transduction and cell death decisions. Methods. 2013 Jun 1;61(2):165-73.

doi: 10.1016/j.ymeth.2013.04.007. Epub 2013 Apr 19. PubMed PMID: 23607991.

102: Murphy ÁC, Weyhenmeyer B, Schmid J, Kilbride SM, Rehm M, Huber HJ, Senft C,

Weissenberger J, Seifert V, Dunst M, Mittelbronn M, Kögel D, Prehn JH, Murphy BM.

Activation of executioner caspases is a predictor of progression-free survival in

glioblastoma patients: a systems medicine approach. Cell Death Dis. 2013 May

16;4:e629. doi: 10.1038/cddis.2013.157. PubMed PMID: 23681224; PubMed Central

PMCID: PMC3674364.

103: Mashayekhi K, O'Brien M, Zugun-Eloae F, Labusca L. Novel approaches for

treating musculoskeletal diseases: molecular orthopedics and systems medicine.

Open Orthop J. 2013 May 3;7:144-51. doi: 10.2174/1874325001307010144. Print 2013.

PubMed PMID: 23798982; PubMed Central PMCID: PMC3664448.

104: Karimiani EG, Day P. Personalised treatment of haematological malignancies

through systems medicine based on single molecules in single cells. Integr Biol

(Camb). 2013 May;5(5):759-67. doi: 10.1039/c3ib20258e. Review. PubMed PMID:

23532213.

105: Hood L. Systems biology and p4 medicine: past, present, and future. Rambam

Maimonides Med J. 2013 Apr 30;4(2):e0012. doi: 10.5041/RMMJ.10112. Print 2013

Apr. PubMed PMID: 23908862; PubMed Central PMCID: PMC3678833.

106: Pantzaris MC, Loukaides GN, Ntzani EE, Patrikios IS. A novel oral

nutraceutical formula of omega-3 and omega-6 fatty acids with vitamins (PLP10) in

relapsing remitting multiple sclerosis: a randomised, double-blind,

placebo-controlled proof-of-concept clinical trial. BMJ Open. 2013 Apr 17;3(4).

pii: e002170. doi: 10.1136/bmjopen-2012-002170. Print 2013. PubMed PMID:

23599375; PubMed Central PMCID: PMC3641495.

107: Tegnér J, Abugessaisa I. Pediatric systems medicine: evaluating needs and

opportunities using congenital heart block as a case study. Pediatr Res. 2013

Apr;73(4 Pt 2):508-13. doi: 10.1038/pr.2013.19. Epub 2013 Jan 31. Review. PubMed

PMID: 23370412.

108: Wolkenhauer O, Auffray C, Jaster R, Steinhoff G, Dammann O. The road from

systems biology to systems medicine. Pediatr Res. 2013 Apr;73(4 Pt 2):502-7. doi:

10.1038/pr.2013.4. Epub 2013 Jan 11. Review. PubMed PMID: 23314297.

109: Sittka A, Vera J, Lai X, Schmeck BT. Asthma phenotyping, therapy, and

prevention: what can we learn from systems biology? Pediatr Res. 2013 Apr;73(4 Pt

2):543-52. doi: 10.1038/pr.2013.8. Epub 2013 Jan 11. Review. PubMed PMID:

23314293.

110: Mayr M. From data gathering to systems medicine. Cardiovasc Res. 2013 Mar

15;97(4):599-600. doi: 10.1093/cvr/cvt017. Epub 2013 Feb 5. PubMed PMID:

23386274; PubMed Central PMCID: PMC3583261.

111: Okser S, Pahikkala T, Aittokallio T. Genetic variants and their interactions

in disease risk prediction - machine learning and network perspectives. BioData

Min. 2013 Mar 1;6(1):5. doi: 10.1186/1756-0381-6-5. PubMed PMID: 23448398; PubMed

Central PMCID: PMC3606427.

112: Klöhn PC, Wuellner U, Zizlsperger N, Zhou Y, Tavares D, Berger S, Zettlitz

KA, Proetzel G, Yong M, Begent RH, Reichert JM. IBC's 23rd Annual Antibody

Engineering, 10th Annual Antibody Therapeutics international conferences and the

2012 Annual Meeting of The Antibody Society: December 3-6, 2012, San Diego, CA.

MAbs. 2013 Mar-Apr;5(2):178-201. doi: 10.4161/mabs.23655. PubMed PMID: 23575266;

PubMed Central PMCID: PMC3893229.

113: Regierer B, Zazzu V, Sudbrak R, Kühn A, Lehrach H. Future of medicine: models

in predictive diagnostics and personalized medicine. Adv Biochem Eng Biotechnol.

2013;133:15-33. doi: 10.1007/10_2012_176. Review. PubMed PMID: 23463359.

114: Zhao Z, Shen B, Lu X, Vongsangnak W. Translational biomedical informatics and

computational systems medicine. Biomed Res Int. 2013;2013:237465. doi:

10.1155/2013/237465. Epub 2013 Dec 2. PubMed PMID: 24350252; PubMed Central

PMCID: PMC3856116.

115: Shen B, Shen HB, Tian T, Lü Q, Hu G. Translational bioinformatics and

computational systems medicine. Comput Math Methods Med. 2013;2013:375641. doi:

10.1155/2013/375641. Epub 2013 May 19. PubMed PMID: 23762184; PubMed Central

PMCID: PMC3671527.

116: Villas-Boas SG. Analytical techniques & applications of metabolomics in

systems medicine and systems biotechnology. Comput Struct Biotechnol J. 2013 Feb

18;4:e201301001. doi: 10.5936/csbj.201301001. eCollection 2013. PubMed PMID:

24688683; PubMed Central PMCID: PMC3962207.

117: Emmert-Streib F, Dehmer M. Enhancing systems medicine beyond genotype data by

dynamic patient signatures: having information and using it too. Front Genet.

2013 Nov 19;4:241. doi: 10.3389/fgene.2013.00241. eCollection 2013. PubMed PMID:

24312119; PubMed Central PMCID: PMC3832803.

118: Dimitrov DV, Hoeng J. Systems approaches to computational modeling of the

oral microbiome. Front Physiol. 2013 Jul 10;4:172. doi: 10.3389/fphys.2013.00172.

eCollection 2013. PubMed PMID: 23847548; PubMed Central PMCID: PMC3706740.

119: Flores M, Glusman G, Brogaard K, Price ND, Hood L. P4 medicine: how systems

medicine will transform the healthcare sector and society. Per Med.

2013;10(6):565-576. PubMed PMID: 25342952; PubMed Central PMCID: PMC4204402.

2012 (17):

120: Leach AR, Wallace K, Alepee N, Daston G, Humphris C, Manou I, Modi S,

Ringeissen S, Whelan M, Kimber I. Reprint of: Characterising hepatic

mitochondrial function as a model for systemic toxicity: a commentary.

Toxicology. 2012 Dec 16;302(2-3):e1-4. doi: 10.1016/j.tox.2012.10.007. Epub 2012

Nov 8. PubMed PMID: 23142426.

121: Hood L, Flores M. A personal view on systems medicine and the emergence of

proactive P4 medicine: predictive, preventive, personalized and participatory. N

Biotechnol. 2012 Sep 15;29(6):613-24. doi: 10.1016/j.nbt.2012.03.004. Epub 2012

Mar 18. PubMed PMID: 22450380.

122: Hood L, Tian Q. Systems approaches to biology and disease enable

translational systems medicine. Genomics Proteomics Bioinformatics. 2012

Aug;10(4):181-5. doi: 10.1016/j.gpb.2012.08.004. Epub 2012 Aug 23. Review. PubMed

PMID: 23084773; PubMed Central PMCID: PMC3844613.

123: Subramaniam S. Evolution of bioengineering at UCSD: opening new vistas. IEEE

Pulse. 2012 Jul;3(4):49-55. doi: 10.1109/MPUL.2012.2198739. PubMed PMID:

22850838.

124: Tretter F, an der Heiden U, Rujescu D, Pogarell O. Computational modelling of

schizophrenic symptoms: basic issues. Pharmacopsychiatry. 2012 May;45 Suppl

1:S2-11. doi: 10.1055/s-0032-1311563. Epub 2012 May 7. Review. PubMed PMID:

22565230.

125: Iris F. Psychiatric systems medicine: closer at hand than anticipated but not

with the expected portrait. Pharmacopsychiatry. 2012 May;45 Suppl 1:S12-21. doi:

10.1055/s-0032-1309002. Epub 2012 May 7. Review. PubMed PMID: 22565229.

126: Holzhütter HG, Drasdo D, Preusser T, Lippert J, Henney AM. The virtual liver:

a multidisciplinary, multilevel challenge for systems biology. Wiley Interdiscip

Rev Syst Biol Med. 2012 May-Jun;4(3):221-35. doi: 10.1002/wsbm.1158. Epub 2012

Jan 13. PubMed PMID: 22246674.

127: Roukos DH. Longevity with systems medicine? Epigenome, genome and environment

interactions network. Epigenomics. 2012 Apr;4(2):119-23. doi: 10.2217/epi.12.1.

PubMed PMID: 22449182.

128: Roukos DH. Biotechnological, genomics and systems-synthetic biology

revolution: redesigning genetic code for a pragmatic systems medicine. Expert Rev

Med Devices. 2012 Mar;9(2):97-101. doi: 10.1586/erd.11.68. Review. PubMed PMID:

22404769.

129: Pristipino C. [Systems medicine as a scientific method for individualizing

therapies in cardiology]. Monaldi Arch Chest Dis. 2012 Mar;78(1):3-5. Italian.

PubMed PMID: 22928396.

130: Tian Q, Price ND, Hood L. Systems cancer medicine: towards realization of

predictive, preventive, personalized and participatory (P4) medicine. J Intern

Med. 2012 Feb;271(2):111-21. doi: 10.1111/j.1365-2796.2011.02498.x. Review.

PubMed PMID: 22142401; PubMed Central PMCID: PMC3978383.

131: Mardinoglu A, Nielsen J. Systems medicine and metabolic modelling. J Intern

Med. 2012 Feb;271(2):142-54. doi: 10.1111/j.1365-2796.2011.02493.x. Review.

PubMed PMID: 22142312.

132: Nielsen J. Translational and systems medicine. J Intern Med. 2012

Feb;271(2):108-10. doi: 10.1111/j.1365-2796.2011.02490.x. PubMed PMID: 22142230.

133: O'Malley MA. Evolutionary systems biology: historical and philosophical

perspectives on an emerging synthesis. Adv Exp Med Biol. 2012;751:1-28. doi:

10.1007/978-1-4614-3567-9_1. Review. PubMed PMID: 22821451.

134: Capobianco E. Ten challenges for systems medicine. Front Genet. 2012 Sep

27;3:193. doi: 10.3389/fgene.2012.00193. eCollection 2012. PubMed PMID: 23060899;

PubMed Central PMCID: PMC3461702.

135: Capobianco E. Dynamic networks in systems medicine. Front Genet. 2012 Sep

19;3:185. doi: 10.3389/fgene.2012.00185. eCollection 2012. PubMed PMID: 23049537;

PubMed Central PMCID: PMC3445994.

136: Yan Q. The role of psychoneuroimmunology in personalized and systems

medicine. Methods Mol Biol. 2012;934:3-19. doi: 10.1007/978-1-62703-071-7_1.

PubMed PMID: 22933138.

2011 (16):

137: Borlawsky TB, Lele O, Payne PR. Research-IQ: development and evaluation of an

ontology-anchored integrative query tool. J Biomed Inform. 2011 Dec;44 Suppl

1:S56-62. doi: 10.1016/j.jbi.2011.07.006. Epub 2011 Jul 29. PubMed PMID:

21821150; PubMed Central PMCID: PMC3251722.

138: Bordbar A, Feist AM, Usaite-Black R, Woodcock J, Palsson BO, Famili I. A

multi-tissue type genome-scale metabolic network for analysis of whole-body

systems physiology. BMC Syst Biol. 2011 Oct 31;5:180. doi:

10.1186/1752-0509-5-180. PubMed PMID: 22041191; PubMed Central PMCID: PMC3219569.

139: Atzei A, Atzori L, Moretti C, Barberini L, Noto A, Ottonello G, Pusceddu E,

Fanos V. Metabolomics in paediatric respiratory diseases and bronchiolitis. J

Matern Fetal Neonatal Med. 2011 Oct;24 Suppl 2:59-62. doi:

10.3109/14767058.2011.607012. Review. PubMed PMID: 21966897.

140: Dothager RS, Piwnica-Worms D. Nano in cancer: linking chemistry, biology, and

clinical applications in vivo. Cancer Res. 2011 Sep 1;71(17):5611-5. doi:

10.1158/0008-5472.CAN-11-0817. Epub 2011 Aug 23. PubMed PMID: 21862634.

141: Madhavan S, Gusev Y, Harris M, Tanenbaum DM, Gauba R, Bhuvaneshwar K,

Shinohara A, Rosso K, Carabet LA, Song L, Riggins RB, Dakshanamurthy S, Wang Y,

Byers SW, Clarke R, Weiner LM. G-DOC: a systems medicine platform for

personalized oncology. Neoplasia. 2011 Sep;13(9):771-83. PubMed PMID: 21969811;

PubMed Central PMCID: PMC3182270.

142: Burke W, Trinidad SB. Systems medicine and the public's health. Genome Med.

2011 Jul 27;3(7):47. doi: 10.1186/gm263. PubMed PMID: 21867575; PubMed Central

PMCID: PMC3221550.

143: Bousquet J, Anto JM, Sterk PJ, Adcock IM, Chung KF, Roca J, Agusti A,

Brightling C, Cambon-Thomsen A, Cesario A, Abdelhak S, Antonarakis SE, Avignon A,

Ballabio A, Baraldi E, Baranov A, Bieber T, Bockaert J, Brahmachari S, Brambilla

C, Bringer J, Dauzat M, Ernberg I, Fabbri L, Froguel P, Galas D, Gojobori T,

Hunter P, Jorgensen C, Kauffmann F, Kourilsky P, Kowalski ML, Lancet D, Pen CL,

Mallet J, Mayosi B, Mercier J, Metspalu A, Nadeau JH, Ninot G, Noble D, Oztürk M,

Palkonen S, Préfaut C, Rabe K, Renard E, Roberts RG, Samolinski B, Schünemann HJ,

Simon HU, Soares MB, Superti-Furga G, Tegner J, Verjovski-Almeida S, Wellstead P,

Wolkenhauer O, Wouters E, Balling R, Brookes AJ, Charron D, Pison C, Chen Z, Hood

L, Auffray C. Systems medicine and integrated care to combat chronic

noncommunicable diseases. Genome Med. 2011 Jul 6;3(7):43. doi: 10.1186/gm259.

PubMed PMID: 21745417; PubMed Central PMCID: PMC3221551.

144: Manahan B. The whole systems medicine of tomorrow: a half-century

perspective. Explore (NY). 2011 Jul-Aug;7(4):212-4. doi:

10.1016/j.explore.2011.04.007. PubMed PMID: 21724152.

145: Oosterhof R, Ith M, Trepp R, Christ E, Flück M. Regulation of whole body

energy homeostasis with growth hormone replacement therapy and endurance

exercise. Physiol Genomics. 2011 Jun 28;43(12):739-48. doi:

10.1152/physiolgenomics.00034.2010. Epub 2011 Mar 29. PubMed PMID: 21447747.

146: Kanodia AK, Kim I, Sturmberg JP. A personalized systems medicine approach to

refractory rumination. J Eval Clin Pract. 2011 Jun;17(3):515-9. doi:

10.1111/j.1365-2753.2011.01669.x. PubMed PMID: 21569182.

147: Bousquet J, Anto J, Auffray C, Akdis M, Cambon-Thomsen A, Keil T, Haahtela

T, Lambrecht BN, Postma DS, Sunyer J, Valenta R, Akdis CA, Annesi-Maesano I, Arno

A, Bachert C, Ballester F, Basagana X, Baumgartner U, Bindslev-Jensen C,

Brunekreef B, Carlsen KH, Chatzi L, Crameri R, Eveno E, Forastiere F,

Garcia-Aymerich J, Guerra S, Hammad H, Heinrich J, Hirsch D, Jacquemin B,

Kauffmann F, Kerkhof M, Kogevinas M, Koppelman GH, Kowalski ML, Lau S,

Lodrup-Carlsen KC, Lopez-Botet M, Lotvall J, Lupinek C, Maier D, Makela MJ,

Martinez FD, Mestres J, Momas I, Nawijn MC, Neubauer A, Oddie S, Palkonen S, Pin

I, Pison C, Rancé F, Reitamo S, Rial-Sebbag E, Salapatas M, Siroux V, Smagghe D,

Torrent M, Toskala E, van Cauwenberge P, van Oosterhout AJ, Varraso R, von

Hertzen L, Wickman M, Wijmenga C, Worm M, Wright J, Zuberbier T. MeDALL

(Mechanisms of the Development of ALLergy): an integrated approach from

phenotypes to systems medicine. Allergy. 2011 May;66(5):596-604. doi:

10.1111/j.1398-9995.2010.02534.x. Epub 2011 Jan 24. Review. PubMed PMID:

21261657.

148: Subramaniam S, Nadeau JH. Pathways, networks, and systems medicine--the

meeting place of the Aegean and the mind. Wiley Interdiscip Rev Syst Biol Med.

2011 May-Jun;3(3):253-4. doi: 10.1002/wsbm.153. Epub 2011 Apr 1. PubMed PMID:

21462354.

149: Nibbe RK, Chowdhury SA, Koyutürk M, Ewing R, Chance MR. Protein-protein

interaction networks and subnetworks in the biology of disease. Wiley Interdiscip

Rev Syst Biol Med. 2011 May-Jun;3(3):357-67. doi: 10.1002/wsbm.121. Epub 2010 Sep

23. Review. PubMed PMID: 20865778.

150: Roukos DH. Trastuzumab and beyond: sequencing cancer genomes and predicting

molecular networks. Pharmacogenomics J. 2011 Apr;11(2):81-92. doi:

10.1038/tpj.2010.81. Epub 2010 Oct 26. Review. PubMed PMID: 20975737.

151: Majumder D, Mukherjee A. A passage through systems biology to systems

medicine: adoption of middle-out rational approaches towards the understanding of

therapeutic outcomes in cancer. Analyst. 2011 Feb 21;136(4):663-78. doi:

10.1039/c0an00746c. Epub 2010 Nov 25. Review. PubMed PMID: 21109854.

152: Wang Z, Bordas V, Deisboeck TS. Discovering Molecular Targets in Cancer with

Multiscale Modeling. Drug Dev Res. 2011 Feb 1;72(1):45-52. PubMed PMID: 21572568;

PubMed Central PMCID: PMC3092304.

2010 (11):

153: Perlman L, Gottlieb A, Atias N, Ruppin E, Sharan R. Combining drug and gene

similarity measures for drug-target elucidation. J Comput Biol. 2011

Feb;18(2):133-45. doi: 10.1089/cmb.2010.0213. PubMed PMID: 21314453.

154: Westerhoff HV. Systems biology left and right. Methods Enzymol.

2011;500:3-11. doi: 10.1016/B978-0-12-385118-5.00001-3. PubMed PMID: 21943889.

155: Chang RL, Xie L, Xie L, Bourne PE, Palsson BØ. Drug off-target effects

predicted using structural analysis in the context of a metabolic network model.

PLoS Comput Biol. 2010 Sep 23;6(9):e1000938. doi: 10.1371/journal.pcbi.1000938.

PubMed PMID: 20957118; PubMed Central PMCID: PMC2950675.

156: Auffray C, Charron D, Hood L. Predictive, preventive, personalized and

participatory medicine: back to the future. Genome Med. 2010 Aug 26;2(8):57. doi:

10.1186/gm178. PubMed PMID: 20804580; PubMed Central PMCID: PMC2945014.

157: Perpiñá Tordera M. [Why do we look at asthma through the keyhole?]. Arch

Bronconeumol. 2010 Aug;46(8):433-8. doi: 10.1016/j.arbres.2010.03.010. Epub 2010

May 11. Review. Spanish. PubMed PMID: 20462683.

158: Parker RS, Clermont G. Systems engineering medicine: engineering the

inflammation response to infectious and traumatic challenges. J R Soc Interface.

2010 Jul 6;7(48):989-1013. doi: 10.1098/rsif.2009.0517. Epub 2010 Feb 10. Review.

PubMed PMID: 20147315; PubMed Central PMCID: PMC2880083.

159: Subramaniam S, Nadeau JH. Systems medicine--viewed through the real and

computing lenses. Wiley Interdiscip Rev Syst Biol Med. 2010 Jul-Aug;2(4):383-4.

doi: 10.1002/wsbm.103. PubMed PMID: 20836036.

160: Borlawsky TB, Li J, Shagina L, Crowson MG, Liu Y, Friedman C, Lussier YA.

Evaluation of an Ontology-anchored Natural Language-based Approach for Asserting

Multi-scale Biomolecular Networks for Systems Medicine. AMIA Jt Summits Transl

Sci Proc. 2010 Mar 1;2010:6-10. PubMed PMID: 21347135; PubMed Central PMCID:

PMC3041541.

161: Roukos DH. Systems medicine: a real approach for future personalized

oncology? Pharmacogenomics. 2010 Mar;11(3):283-7. doi: 10.2217/pgs.10.36. Review.

PubMed PMID: 20235782.

162: Wang K, Lee I, Carlson G, Hood L, Galas D. Systems biology and the discovery

of diagnostic biomarkers. Dis Markers. 2010;28(4):199-207. doi:

10.3233/DMA-2010-0697. Review. PubMed PMID: 20534905; PubMed Central PMCID:

PMC3021550.

163: Yan Q. Systems biology of influenza: understanding multidimensional

interactions for personalized prevention and treatment. Methods Mol Biol.

2010;662:285-302. doi: 10.1007/978-1-60761-800-3_14. PubMed PMID: 20824477.

2009 (6):

164: Clermont G, Auffray C, Moreau Y, Rocke DM, Dalevi D, Dubhashi D, Marshall

DR, Raasch P, Dehne F, Provero P, Tegner J, Aronow BJ, Langston MA, Benson M.

Bridging the gap between systems biology and medicine. Genome Med. 2009 Sep

29;1(9):88. doi: 10.1186/gm88. PubMed PMID: 19754960; PubMed Central PMCID:

PMC2768995.

165: Federoff HJ, Gostin LO. Evolving from reductionism to holism: is there a

future for systems medicine? JAMA. 2009 Sep 2;302(9):994-6. doi:

10.1001/jama.2009.1264. PubMed PMID: 19724047.

166: Lehr S, Meyer HE. Integrative proteomics for the future US HUPO Fifth Annual

Conference from genes to function 22-25 February 2009, San Diego, CA, USA.

Proteomics Clin Appl. 2009 Jul;3(7):756-7. doi: 10.1002/prca.200900052. Epub 2009

Jun 25. PubMed PMID: 21136984.

167: Tolvanen M, Ojala PJ, Törönen P, Anderson H, Partanen J, Turpeinen H.

Interspliced transcription chimeras: neglected pathological mechanism

infiltrating gene accession queries? J Biomed Inform. 2009 Apr;42(2):382-9. doi:

10.1016/j.jbi.2008.11.002. Epub 2008 Nov 13. PubMed PMID: 19041732.

168: Auffray C, Chen Z, Hood L. Systems medicine: the future of medical genomics

and healthcare. Genome Med. 2009 Jan 20;1(1):2. doi: 10.1186/gm2. PubMed PMID:

19348689; PubMed Central PMCID: PMC2651587.

169: Orešič M, Lötjönen J, Soininen H. Systems medicine and the integration of

bioinformatic tools for the diagnosis of Alzheimer's disease. Genome Med.

2009;1(11):83. doi: 10.1186/gm204. Epub 2010 Nov 15. PubMed PMID: 21092145;

PubMed Central PMCID: PMC3016625.

2008 (3):

170: Shim EB, Lee S, Kim JY, Earm YE. Physiome and Sasang Constitutional

Medicine. J Physiol Sci. 2008 Dec;58(7):433-40. doi: 10.2170/physiolsci.RV004208.

Epub 2008 Oct 21. Review. PubMed PMID: 18928639.

171: Costa J. Systems medicine in oncology. Nat Clin Pract Oncol. 2008

Mar;5(3):117. doi: 10.1038/ncponc1070. PubMed PMID: 18305488.

172: Yan Q. The integration of personalized and systems medicine: bioinformatics

support for pharmacogenomics and drug discovery. Methods Mol Biol. 2008;448:1-19.

doi: 10.1007/978-1-59745-205-2_1. Review. PubMed PMID: 18370227.

2007 (2):

173: Li W, Xiao DM, Jiang HP. [Exploration of systems medicine in the 21st

century]. Zhonghua Yi Xue Za Zhi. 2007 Aug 21;87(31):2172-5. Chinese. PubMed

PMID: 18001524.

174: Liu ET, Lemberger T. Higher order structure in the cancer transcriptome and

systems medicine. Mol Syst Biol. 2007;3:94. Epub 2007 Mar 13. PubMed PMID:

17353936; PubMed Central PMCID: PMC1847947.

2006 (7):

175: Baranzini SE. Systems-based medicine approaches to understand and treat

complex diseases. The example of multiple sclerosis. Autoimmunity. 2006

Dec;39(8):651-62. Review. PubMed PMID: 17178562.

176: Katoh Y, Katoh M. Comparative integromics on FAT1, FAT2, FAT3 and FAT4. Int

J Mol Med. 2006 Sep;18(3):523-8. PubMed PMID: 16865240.

177: Katoh Y, Katoh M. Canonical WNT signaling pathway and human AREG. Int J Mol

Med. 2006 Jun;17(6):1163-6. PubMed PMID: 16685431.

178: Hyman MA. The evolution of research: meeting the needs of systems medicine,

part 1. Altern Ther Health Med. 2006 May-Jun;12(3):10-1. Review. PubMed PMID:

16708765.

179: Katoh Y, Katoh M. Comparative integromics on BMP/GDF family. Int J Mol Med.

2006 May;17(5):951-5. PubMed PMID: 16596286.

180: Katoh M, Katoh M. Comparative integromics on Eph family. Int J Oncol. 2006

May;28(5):1243-7. PubMed PMID: 16596241.

181: Liu ET, Kuznetsov VA, Miller LD. In the pursuit of complexity: systems

medicine in cancer biology. Cancer Cell. 2006 Apr;9(4):245-7. Review. PubMed

PMID: 16616330.

2005 (0):

2004 (1):

182: Tao Y, Liu Y, Friedman C, Lussier YA. Information Visualization Techniques

in Bioinformatics during the Postgenomic Era. Drug Discov Today Biosilico. 2004

Nov;2(6):237-245. PubMed PMID: 20976032; PubMed Central PMCID: PMC2957900.

2003 (0):

2002 (1):

183: Taanila A, Larivaara P, Korpio A, Kalliokoski R. Evaluation of a

family-oriented continuing medical education course for general practitioners.

Med Educ. 2002 Mar;36(3):248-57. PubMed PMID: 11879515.

2001-1994 (0):

1993 (1):

184: Wood BL. Beyond the "psychosomatic family": a biobehavioral family model of

pediatric illness. Fam Process. 1993 Sep;32(3):261-78. PubMed PMID: 8243617.

1992 (1):

185: Lieberman S. Family therapy. Postgrad Med J. 1992 Nov;68(805):921-4. PubMed

PMID: 1494515; PubMed Central PMCID: PMC2399469.
