# Supplementary material for: Applying systems biology to biomedical research and health care: a précising definition of systems medicine
Source: BMC Health Serv Res. 2017 Nov 21;17:761. doi: 10.1186/s12913-017-2688-z (PMC5698952; doi:10.1186/s12913-017-2688-z)
Supplement: Supplementary file 6 — Annual number of papers in research context with reference to specific disease (DOCX 15 kb) [file 12913_2017_2688_MOESM6_ESM.docx]

Annual number of papers in research context with reference to specific disease
