# Supplementary material for: Applying systems biology to biomedical research and health care: a précising definition of systems medicine
Source: BMC Health Serv Res. 2017 Nov 21;17:761. doi: 10.1186/s12913-017-2688-z (PMC5698952; doi:10.1186/s12913-017-2688-z)
Supplement: Supplementary file 10 — Overview of all definitions and definition-like text passages identified (DOCX 32 kb) [file 12913_2017_2688_MOESM10_ESM.docx]

**Overview of all definitions and definition-like text passages identified**

| **Number** | **Quotation** |
| --- | --- |
| **3** | “Systems medicine” combines systems biology and pathophysiological approaches to translational research, integrating various bio-medical tools and using the power of computational and mathematical modelling. This enables the personalization of diagnosis, prognosis and treatment. Systems medicine helps to re-define clinical phenotypes using molecular and dynamic parameters to discover new diagnostic and prognostic biomarkers and to guide the design of new clinical trials.  […the “systems biology” approach, i.e. the interdisciplinary study of complex interactions within differentbiological systems. “Systems pathophysiology” studies the complex interactions between major human vital systems and their interplay.] |
| **8** | An important goal of systems medicine is to generate genomics informed personalized therapeutic regimes with higher efficacy. The ability of inferred models to accurately predict sensitivity of an individual tumor to a drug or drug combination can assist in designing personalized cancer therapy treatments with expected effectiveness significantly higher than current standard of care approaches. |
| **9** | Systems medicine driven approaches incorporating genomic information (genomic medicine) along with appropriate biological and computational tools for data interpretation will be used to deliver P4 and precision medicine in the future. This will enable introduction of individualized tailored prevention and/or treatment strategies.  [‘P4 medicine’ consists of predictive, preventive, personalized and participatory medicine. ‘Precision medicine’ includes development of prevention and treatment strategies that take individual variability into account.] |
| **13** | Precision medicine tailors prevention, diagnosis, therapeutics, and prognosis for each patient. Related to precision medicine is systems medicine, which leverages systems biology for clinical application, with resulting data termed “systems medicine data”. Systems biology studies the behavior of organisms or cells as whole systems, and uses various advances in biotechnology, including genomics, transcriptomics, proteomics, metabolomics, methylomics, microbiomics, and elucidation of cellular interaction networks by network biology. Often, systems medicine data from these various advances can be modeled and simulated with complementary computer science, mathematics, chemistry, physics, and engineering concepts in computational biology. |
| **21** | ...introduced the subject of systems medicine by explaining how information and communication technologies, and the conceptual framework of complex system studies can be used to understand the critical points of health maintanance and prevent disease development.  ...described the systems medicine approach to aid understanding of the nonpulmonary determinants of heterogeneity in the common and debiliating condition of chronic obstructive pulmonary disease (COPD). |
| **23** | Systems medicine approaches can therefore be employed for shedding light in multiple research scenarios, ultimately leading to the practical result of uncovering novel dynamic interaction networks that are critical for influencing the course of medical conditions. Consequently, systems medicine also serves to identify clinically important molecular targets for diagnostic and therapeutic measures against such a condition  Systems medicine (sometimes referred to as systems healthcare) promises to lead with clinical and molecular know-how to produce exquisite datasets that are employed to generate pathway models and treatment and will hopefully directly contribute to stratified medicine en-route to personalized healthcare [4, 8–11].  The application of systems biology within the remit of present day medical research can be defined as systems medicine […]  In essence, this change in research perspective by scrutinizing overall molecular network interactions, rather than individual molecules, allows for more effective and clinically applicable research outcomes. |
| **26** | we demonstrate that our approach links disease-associated genes to the phenotypes they produce, a key goal within systems medicine. |
| **28** | One idea is to define SM as an implementation of Systems Biology (SB) in the Medical disciplines with a particular attention to clinical applications [1], including clinical Bioinformatics and the discrimination of pathological states and related morbidities and comorbidities The extension of SB (Table 1) to the clinical practice implies the establishment of a connection between a molecular-centered to a patient-centered world, through an organ-centered intermediate layer. This mapping (Figure 1) requires the extensive use of computational tools such as statistical, mathematical and bioinformatical techniques  Systems Medicine (SM) can be defined as an extension of Systems Biology (SB) to Clinical-Epidemiological disciplines through a shifting paradigm, starting from a cellular, toward a patient centered framework. According to this vision, the three pillars of SM are Biomedical hypotheses, experimental data, mainly achieved by Omics technologies and tailored computational, statistical and modeling tools. The three SM pillars are highly interconnected, and their balancing is crucial  The definition of SM is deeply related to complex networks: it involves a systemic view of the organism where the various building elements are considered in their interplay [16, 93, 94]. |
| **29** | Systems Medicine platforms represent the most suitable approach to personalized medicine, enabling to identify new patterns in the pathogenesis, diagnosis and prognosis of chronic diseases. |
| **30** | The ‘precision medicine (systems medicine)’ concept promises to achieve a shift to future healthcare systems with a more proactive and predictive approach to medicine, where the emphasis is on disease prevention rather than the treatment of symptoms. The individualization of treatment for each patient will be at the centre of this approach, with all of a patient’s medical data being computationally integrated and accessible  ‘Precision medicine’ has emerged as a computational approach to functionally interpret omics and big data, and facilitate their application to healthcare provision. [In this new era, patients are not segregated by disease, or disease subtype.] Instead, the aim is to treat every patient as an individual case, incorporating a range of personalized data including genomic, epigenetic, environmental, lifestyle and medical history. The aspiration is that the accumulation of these data into and individualized virtual representation of the patient, combined with predictive modelling based on known interactions will inform rational therapy design for each patient (Figure 1).  To achieve these goals, precision medicine aims to develop computational models that integrate data and knowledge from both clinic and basic research to gain a mechanistic understanding of disease [10], thereby facilitating personalized treatment decisions. |
| **31** | Systems medicine analyzes the dynamic data cloud that surrounds each patient and uses this to derive “actionable possibilities” that can improve wellness or avoid disease for each patient.  predictive, preventive, personalized, and participatory medicine. This extension of systems biology was coined systems medicine.14  The new systems medicine allows a strategy for looking at the earliest transition of wellness to disease—and opens the possibility of developing new diagnostic and therapeutic reagents  to terminate a disease trajectory for each individual early, returning them to wellness. Second, these approaches rely on data as the primary modeling material, not knowledge.  Systems medicine, which purports to design multiscale mathematical disease models,  Systems medicine aims at predicting the course of a disease in a given patient and how far it can be altered by available therapies.  The fundamental principle of systems medicine should thus be the prediction of benefit–risk for a single subject, a group, or a population. |
| **34** | […] systems medicine, the application of systems biology to medicine. The underlying philosophy looks at biology as ‘information science’ and is concerned with the network of molecular interactions that define biological processes [14,15]. Additionally, disease states are viewed as a perturbation of these molecular networks [15]. In the case of traditional TBI biomarker discovery, the approach has been to seek an individual molecule to represent a disease state, while disregarding any notion of a network let alone its perturbation.  […] systems biology (and by extension, systems medicine), which is concerned with the complex network interplay of a biological unit and represents injury and illness as a perturbation to the network. |
| **35** | Systems medicine amalgamates systems biology techniques with medical treatment decision-making, where information from many biological measurements is combined and analysed for complex patterns of change.19 |
| **36** | […] systems biology is evolving to systems medicine as a new discipline that aims to offer new approaches for addressing the diagnosis and treatment of major human diseases uniquely, effectively, and with personalized precision.  Systems medicine is not simply the application of systems biology in medicine; rather, it is the logical next step and necessary extension of systems biologywithmore emphasis on clinically relevant applications.17 Building on the success of systems biology, systems medicine is defined as an emerging discipline that integrates comprehensively computational modeling, ’omics data, clinical data, and environmental factors to model and predict disease expression (the pathophenome).17,18 Systems medicine integrates basic research and clinical practice, and emphasizes translational and clinical research.  Systems medicine is highly comprehensive and integrative, and utilizes all types of nonlinear information.  Systems medicine aims to offer a powerful set of methodologies to improve our understanding of disease pathogenesis and to design personalized therapies to address the complexity of human diseases. |
| **37** | Historically, Systems Medicine has been defined as the clinical application of Systems Biology approaches to medicine, where traditional model-driven experiments are informed by data-driven models in an iterative manner [25]. We see Systems Medicine as the long-term objective of a wider paradigm shift in medical science, at the end of which a range of different models and approaches will coexist under the Systems Medicine umbrella. All of these models will be substantially more complex than the models used in Reductionism or Network Medicine. We suggest that Systems Medicine models should include two or more of the following organizing principles of the human body: non-linearity, multi-agency, multi-levelness, or adaptivity [26]. |
| **45** | Systems biology methodologies could be applied to the study of diseases, allowing us to identify diagnostics and therapeutics connected through molecular components and this general approach is now known as systems medicine [47]. The crucial principle of systems medicine is that molecular fingerprints resulting from biological networks perturbed by the disease will be used to detect and stratify various pathological conditions. Central to the systems medicine approach is the use of network-based models of biological process combined with the information on the patient, mainly of molecular origin [47]. Systems medicine integrates physiopathology, network biology and molecular variations, providing novel insights into the mechanisms of various diseases, such as diabetes [48] and obesity [49], overcoming the current limitations of disease complexity, possibly through stratification of patients and diseases. |
| **47** | Systems Medicine is the new discipline that emerges as a translational extension of systems biology, an interdisciplinary approach that systematically describes the complex interactions between all parts of a biological system, with a view to elucidating new biological rules capable of predicting the behavior of the biological system. To this aim, data are collected from all the components of the immune system, analyzed and integrated in order to generate a mathematical model that describes or predicts the response of the system to individual perturbations. |
| **56** | Systems Medicine, as an adaptation and extension of Systems Biology, embraces this paradigm and is becoming a cornerstone in the study of complex diseases. |
| **57** | “An integrative approach to medical needs taking advantage and emphasizing information and tools made available by the greatest possible spectrum of scientific disciplines, aimed at improving risk prediction and individual treatment respecting ethical and legal requirements. This approach should improve medical practice by standardization, information, integration, monitoring and personalization”. |
| **59** | Systems medicine [2–4] – defined as ‘the application of systems biology to medical research and practice’ |
| **60** | Systems biology or systems medicine is a complementary approach aimed at analyzing the interactions between the different components within one organizational level (genome, transcriptome, proteome), and then between the different levels. |
| **61** | Systems medicine is an emerging discipline that aims to find novel diagnostic markers and therapeutic targets by combining omics with bioinformatics, as well as functional and clinical studies. |
| **71** | Systems biology for disease modelling and treatment, or systems medicine, represents an innovative approach to complex diseases understanding and drug discovery. It consists of representing all the available knowledge on the disease of interest with a mathematical symbolism allowing generation and testing of hypotheses through computational simulation and experimental validation. |
| **77** | Systems medicine is the application of systems biology to medical research and medical practice. Its objective is to integrate a variety of data at all relevant levels of cellular organisation with clinical and patientreported disease markers, using the power of computational and mathematical modelling, to enable the understanding of the mechanisms, prognosis, diagnosis and treatment of disease. |
| **78** | Systems Medicine applies the perspective of SB [Systems Biology] to the study of disease mechanisms, with the aim of improving the diagnostic process, disease management, and outcomes. |
| **83** | This [SM] is an emerging discipline that aims to gain a translational understanding of the complex mechanisms underlying common diseases.  Systems medicine is an emerging discipline that aims to address the problem that a disease is rarely caused by malfunction of one individual gene product, but instead depends on multiple gene products that interact in a complex network.  We focus on systems medicine as a network-based approach to analysis of high-throughput and routine clinical data to predict disease mechanisms to diagnoses and treatments.  Some view it as an interdisciplinary approach that integrates research data and clinical practice and others view it as fusion of systems biology and bioinformatics with a focus on disease and the clinic. Recent articles have described systems medicine as a high-precision, mathematical model of variables from different genomic layers that relate to clinical outcomes such as treatment response.  […] systems medicine is a natural extension of, or is complementary to, current models for clinical decision-making. |
| **84** | Systems medicine is an interdisciplinary approach that integrates data from basic research and clinical practice to improve our understanding and treatment of diseases. Systems medicine can be seen as a further development of systems biology and bioinformatics towards applications of clinical relevance. The term 'systems' refers to systems approaches, emphasizing a close integration of data generation with mathematical modeling.  The aim is to derive a mechanistic understanding of pathologies, prophylaxy and support of therapy optimization. This requires the development of concepts, methods and tools that support the integration of organizational levels to develop interfaces between the computational and mathematical frameworks used in systems medicine. |
| **86** | Systems biology is an interdisciplinary effort to integrate molecular, cellular, tissue, organ, and organism levels of function into computational models that facilitate the identification of general principles. Systems medicine adds a disease focus.  Systems medicine applies the tools and concepts from systems biology and addresses complexity in two key ways. First, systems medicine uses molecular diagnostics to stratify patients and diseases to better characterize and understand disease complexity. By applying a network-level view of disease to create disease networks, systems medicine will overcome current limitations in drug discovery by identifying important functional and regulatory modules within these networks. Then, by analyzing and targeting hubs—the most highly interconnected nodes—within these regulatory networks, and enzymatic activity in metabolic networks, network-based approaches will be able to explore the effects of various drugs in mathematical models. |
| **88** | […] discuss the scientific and clinical progress made over the past few years towards unravelling the complexity of airway diseases, from the definition of clinical phenotypes and endotypes to a better understanding of cellular and molecular networks as key pathogenic elements of human diseases (so-called systems medicine). |
| **91** | ‘Systems Medicine involves the implementation of Systems Biology approaches in medical concepts, research and practice, through iterative and reciprocal feedback between data-driven computational and mathematical models as well as model-driven translational and clinical investigations’. Systems medicine is therefore where specific but large and static data sets acquired across multiple modalities are used to construct computational models for the dynamic prediction of disease progression or response to treatment at a personal level. |
| **92** | Systems medicine should be understood as application of the systems biology approach to disease-focused or clinically relevant research problems. |
| **96** | While systems medicine can provide a conceptual and theoretical framework, its practical goal is to provide physicians the tools necessary for harnessing the rapid advances in basic biomedical science into their routine clinical arsenal.  systems medicine aims to provide the tools to take into account the complexity of the human body and disease in the everyday medical practice. |
| **98** | […] Systems Medicine, whose objective is to answer clinical questions based on theoretical methods and high-throughput “omics” data.  […] Systems Medicine, an emerging field aiming to provide answers to clinical questions based on theoretical methods and high-throughput “omics” data. |
| **99** | […]integrated “systems medicine” approaches in which clinical decision making is supported by statistical and computational analysis of metabolic, phenotypic, and physiological data.  […] “systems medicine”, the integrated study of system level metabolic, phenotypic, and physiological changes in response to disease processes or therapies by application of computational and statistical approaches to support clinical decisions. |
| **101** | […] systems medicine, i.e. the application of systems biology in a clinical context, […] |
| **103** | However, systems medicine is not the mere translation of the terminology from computer and life sciences to the medical field. From the systemic thinking perspective, a human being is a complex system with structural multiplicity and multilevel hierarchies organized as a scale-free network. It functions as an open informational system characterized by robustness, self organization, adaptability and connectivity. Digital biological information, genome encoded or epigenetic, is integrated and transferred in biological networks. Information is coded hierarchically in DNA, RNA, proteins, differentiated cells, tissues, organs, organisms, and the environment. The system is characterized by multilevel interactions within networks with interdependent connectivity. |
| **104** | Rooted in proteome and metabolome analytics for helping to deconvolute complex data sets and help to relate molecules to function, systems biology has been an academic discipline largely dedicated to deciphering the control mechanisms existing within model organisms such as yeast and has developed tools for data integration. Systems models of disease require sophisticated measurement of molecular moieties. For humans this is being termed systems medicine or systems healthcare. |
| **105** | Systems medicine has united genomics and genetics through family genomics to more readily identify disease genes. |
| **106** | The uniqueness of systems medicine (SM) is the recognition that different specific complex factors are important in disease management and that these factors need to be incorporated in some meaningful way for treatment selection and delivery. |
| **107** | […] the application of a systems biology approach in medical research and clinical practice has defined the rise of systems medicine over the past years.  The core concept of a systems medicine approach is to intervene at an early stage to prevent the occurrence and reduce the suffering of the effects of disease, in contrast to chiefly targeting reactive measures only following the occurrence of disease.  […] a systems medicine approach embraces and includes programs such as P4 medicine and personalized medicine.  […] at the core of a systems medicine analysis is the data integration from omics to the clinic, which requires standardization of data. |
| **108** | […]systems medicine is the logical next step and necessary extension of systems biology […]  Although systems biology is an approach by which biological questions are addressed through integrating experiments in iterative cycles with computational modeling, simulation, and theory (15), systems medicine carries this approach forward into a disease-oriented era, driven by clinical and public health needs (16). |
| **118** | “Systems medicine” is the application of systems biology approaches to medical research and medical practice. |
| **119** | […] ‚systems medicine‘: the application of systems biology to the challenge of human disease. |
| **121** | […] systems medicine, a systems approach to health and disease.  Systems medicine, the child of systems biology,  […] systems medicine is all about identifying all the components of a system, establishing their interactions and assessing their dynamics – both temporal and spatial – as related to their functions.  Systems medicine […] is holistic and utilizes all types of biological information – DNA, RNA, protein, metabolites, small molecules, interactions, cells, organs, individuals, social networks and external environmental signals – integrating them so as to lead to predictive and actionable models for health and disease. |
| **122** | We are at the dawn of predictive, preventive, personalized, and participatory (P4) medicine, the fully implementation of which requires marrying basic and clinical researches through advanced systems thinking and the employment of high-throughput technologies in genomics, proteomics, nanofluidics, single-cell analysis, and computation strategies in a highly-orchestrated discipline we termed translational systems medicine. |
| **124** | “Systems Medicine”, which is defined as “the application of systems biology approaches to medical research and medical practice. Ist objective is to integrate a variety of biological/medical data on all relevant levels of cellular organization, using the power of computational and mathematical modelling, to enable an understanding of the pathophysiological mechanisms, prognosis, diagnosis and treatment of disease [ 1 ]. In other words, (molecular) systems biology in medicine aims to represent signs and symptoms of diseases in multi-level computational models of cells, tissues, organs, organ systems and even organisms [ 2 – 5 ]. |
| **126** | […] systems medicine aims to reconstruct organs and organisms using knowledge of their molecular components to determine clinical behaviours and interventions [ 26 ].  […] a holistic  approach to medicine (systems medicine), that could benefit patients and society, must exploit more limited data sets, arising from multiple open-ended investigations upon highly heterogeneous patient populations in conjunction with vast amounts of poorly correlated published results.Hence, systems medicine must proceed on the basis of existing, highly heterogeneous data and not on the basis of homogeneous datasets arising from specifically targeted investigations. |
| **130** | While some aspects of systems medicine are being adopted in clinical oncology practice through companion moleculardiagnostics for personalized therapy, the mounting influx of global quantitative data from both wellness and diseases, is shaping up a transformational paradigm in medicine we termed predictive, preventive, personalized, and participatory (P4) medicine, which requires new strategies, both scientific and organizational, to enable bringing this revolution in medicine to patients and to the healthcare system. |
| **131** | The reconstruction of such biological network models, the combination of these models with omics data and their application to specific medical questions are often referred to as systems medicine.  Systems medicine allows for a better understanding of the structure and function of the human genome and its associations by determining the links between genotypes, phenotypes and environmental factors (e.g. diet and exposure to toxins) [15] (Fig. 1). The integrative systems medicine research approach helps to understand the behaviour of the human body at all levels of organization by analysing its different constituents. Subsequently, it offers the prospects of modelling complex diseases, establishing novel diagnostic and therapeutic techniques [16], identifying new drug targets [17], developing a system-orientated drug design strategy [18] and eventually achieving effective personalized medicine [19, 20]. |
| **134** | Systems Medicine (Auffray et al., 2009, 2010) emphasizes the role of systems biology in medical/clinical applications. With the advent of new technologies, the “omics” explosion (i.e., next generation sequencing) and the induced changes from data-poor to data-rich applications (for instance related to high-content imaging, physiology, and structural biology) have established the necessity of a systems approach (Noble, 2008) not to be caught in the data deluge.  Systems medicine represents a mosaic of distinct and interconnected micro-systems allowing to infer the macro-systems dynamics and produce elements of synthesis such as signatures (Hood and Friend, 2011; Sung et al., 2012) and profiles originated by a variety of information sources and consequently characterized. |
| **141** | The new and emerging field of systems medicine, an application of systems biology approaches to biomedical problems in the clinical setting, leverages complex computational tools and high-dimensional data to derive personalized assessments of disease risk. Systems medicine offers the potential for more effective individualized diagnosis, prognosis, and treatment options. Achieving this goal requires the effective use of petabytes of data, which necessitates the development of both new types of tools and a new type of physician—one with a grasp of modern computational sciences, “omics” technologies, and a systems approach to the practice of medicine.  While systems biology will provide the foundation for a practice of systems medicine in the future that will be predictive, personalized, preventive, and participatory |
| **142** | Systems or ‘P4’ medicine offers a grand vision for achieving better population health. The four Ps - predictive, preventive, personalized and participatory - invoke a patient-centered approach that prioritizes health promotion over disease treatment. |
| **143** | This proposed holistic strategy involves comprehensive patient-centered integrated care and multi-scale, multi-modal and multi-level systems approaches to tackle NCDs as a common group of diseases. Rather than studying each disease individually, it will take into account their intertwined gene-environment, socio-economic interactions and co-morbidities that lead to individual-specific complex phenotypes. It will implement a road map for predictive, preventive, personalized and participatory (P4) medicine based on a robust and extensive knowledge management infrastructure that contains individual patient information. It will be supported by strategic partnerships involving all stakeholders, including general practitioners associated with patient-centered care. This systems medicine strategy, which will take a holistic approach to disease, is designed to allow the results to be used globally, taking into account the needs and specificities of local economies and health systems.  Systems medicine is the application of systems biology to medical research and practice [54,55]. Its objective is to integrate a variety of data at all relevant levels of cellular organization with clinical and patient-reported disease markers. It uses the power of computational and mathematical modeling to enable understanding of the mechanisms, prognosis, diagnosis and treatment of disease [56]. It involves a transition to predictive, preventive, personalized and participatory (P4) medicine, which is a shift from reactive to prospective medicine that extends far beyond what is usually covered by the term personalized medicine  The ‘systems medicine’ concept, which takes a holistic view of health and disease, encapsulates this perspective. Systems medicine aims to tackle all components of the complexity of NCDs so as to understand these various phenotypes and hence enable prevention (Box 2), control through health promotion [50] and personalized medicine [51], and an efficient use of health service resources [52]. It does this through integrated care using multidisciplinary and teamwork approaches centered in primary and community |
| **146** | Understanding the unique events in an individual’s life as influencing the development of illness and disease appears to be the key to what is emerging under the names of ‘personalized medicine’ and ‘systems medicine’. Personalized medicine presupposes systems biology and complexity sciences, […] |
| **148** | Systems biology and [!?] medicine focuses on deciphering mechanisms at multiple levels, reconstructing networks in cells, tissues and organs, measuring and predicting phenotypes, building quantitative models that describe and simulate normal and pathological physiological functions, and then testing the validity of these models and predictions experimentally. |
| **149** | The main goal of systems medicine is to provide predictive models of the pathophysiology of complex diseases as well as define healthy states. |
| **150** | Similarly, exploration of tumor microenvironment2,15 and of a more global approach to link individual tumors with their multiple host variables, including heritable causal mutations, environmental exposures and lifestyle, are of fundamental importance and represent systems medicine approaches. |
| **153** | Understanding drugs and their modes of action is a fundamental challenge in systems medicine. Key to addressing this challenge is the elucidation of drug targets, an important step in the search for new drugs or novel targets for existing drugs. Incorporating multiple biological information sources is of essence for improving the accuracy of drug target prediction. |
| **157** | […] new strategies capable of integrating all known information about the elements that make up the reality called asthma, thus offering a detailed mapping of its complexity. This approach is known under the generic name of systems-oriented medicine and the applicable methodology tool, systems biology. |
| **158** | […] systems medicine, as a translationally relevant extension of systems biology (Auffray et al. 2009).  Systems medicine, the translational science counterpart to basic science’s systems biology, is the interface at which these tools may be constructed.  […] systems medicine is the coupling of systems science with medical treatment decision-making (Auffray et al. 2009). |
| **160** | systems medicine approaches focus on the dynamic interactions among multiple factors that affect complex diseases, such as diabetes, coronary artery disease and cancers1. The increasing availability of powerful high-throughput technologies, computational tools and integrated knowledge bases, has made it possible to establish new links between genes, biologic functions and human diseases, providing the hallmarks of systems medicine, including signatures of pathology biology, and links to clinical research and drug discovery2. Holistic systems biology methodologies promise to provide the foundation for such prospective medicine through the construction of integrated biomolecular networks3. |
| **162** | The knowledge of network dynamics through in vitro experimental perturbation and modeling allows us to determine the state of the networks, to identify molecular correlates, and to derive new disease treatment approaches to reverse the pathology or prevent its progress into a more severe state through the manipulation of network states. This general approach, including diagnostics and therapeutics, is becoming known as systems medicine.  The transformation in biology through systems biology will enable a new medical discipline – systems medicine – to begin to emerge in the next few years. The central premise of systems medicine is that clinically detectable molecular fingerprints resulting from disease-perturbed biological networks will be used to detect and stratify various pathological conditions. Disease associated molecular fingerprints will eventually be used to group individuals into sub-populations based on variations in genetic makeup of the population that affects disease progression. The key to this revolution lies in harnessing the power of network models of core biological processes learned through systems biology methods, combined with vast amounts of diverse molecular information generated from patient samples.  The therapeutic component of systems medicine then, in which we infer network states from biomarker measurements and intervene to halt and reverse the networks progress into an undesired state, depends on our ability to: 1) precisely infer network state from the results of assessing the levels  of a panel of informative, diagnostic biomarkers in the blood, and 2) specifically manipulate a network to avoid or revert the pathology.  The future of systems medicine will ultimately be the application of our understanding of the integrated dynamical responses of various molecular networks that determine the critical states of the body. |
| **164** | […] that systems medicine be defined as the application of systems biology to the prevention of, understanding and modulation of, and recovery from developmental disorders and pathologic processes in human health.  […] systems medicine emphasizes that the essential purpose and relevance of models is translational, aimed at diagnostic, predictive, and therapeutic applications.  […] systems medicine aims to discover and select the key factors at each level and integrate them into models of translational relevance, which include measurable readouts and clinical predictions. |
| **165** | […] “systems medicine,” which incorporates the complex biochemical, physiological, and environmental interactions that sustain living organisms.  […] systems medicine incorporates interactions between all components of health and disease.  A key feature of systems medicine is that existing networks, through dynamic (time-dependent) interactions, manifest “emergent properties” that define the whole and that these properties are not simply the sum of the features of its component parts. |
| **170** | Systems medicine […] tries to understand perturbed physiological systems and complex pathologies in their entirety by integrating all levels of quantitative functional, structural, and morphological information into a coherent model. In contrast to systems biology, systems medicine seeks an integrative and systemic approach for the diagnosis, therapy, and prevention of diseases [47]. It investigates the physiological network of diseases from gene to organ systems with four main goals — predictive, preventive, personalized, and participative medicine (P4 medicine). |
| **171** | via an integrative approach that includes clinical examinations, experimental modeling and in-silico simulation. Systems medicine seeks to understand perturbed physiological systems and complex pathologies in their entirety by integrating all levels of quantitative functional, structural and morphological information into a coherent model. In contrast to systems biology, systems medicine is geared towards obtaining clinical impact with both diagnostic and therapeutic end points. |
| **175** | Systems medicine is an emerging concept that acknowledges the complexity of a multitude of non-linear interactions among molecular and physiological variables. Under this new paradigm, rather than a collection of symptoms, diseases are seen as the product of deviations from a robust steady state compatible with life. This concept requires the incorporation of mathematics and physics to the more classical arsenal of physiology and molecular biology with which physicians are trained today. |
