# Supplementary material for: Applying systems biology to biomedical research and health care: a précising definition of systems medicine
Source: BMC Health Serv Res. 2017 Nov 21;17:761. doi: 10.1186/s12913-017-2688-z (PMC5698952; doi:10.1186/s12913-017-2688-z)
Supplement: Supplementary file 11 — List of all definitions divided into means and ends (DOCX 30 kb) [file 12913_2017_2688_MOESM11_ESM.docx]

**List of all definitions divided into means and ends**

| **Number** | **Means** | **Ends** | |  |
| --- | --- | --- | --- | --- |
| **3** | combines systems biology and pathophysiological approaches to translational research, integrating various bio-medical tools and using the power of computational and mathematical modelling  using molecular and dynamic parameters | enables the personalization of diagnosis, prognosis and treatment  helps to re-define clinical phenotypes  to discover new diagnostic and prognostic biomarkers  to guide the design of new clinical trials | |  |
| **8** | inferred models | accurately predict sensitivity of an individual tumor to a drug or drug combination  to generate genomics informed personalized therapeutic regimes with higher efficacy  assist in designing personalized cancer therapy treatments with expected effectiveness significantly higher than current standard of care approaches | |  |
| **9** | incorporating genomic information (genomic medicine) along with appropriate biological and computational tools for data interpretation | to deliver P4 and precision medicine in the future. This will enable introduction of individualized tailored prevention and/or treatment strategies | |  |
| **13** | leverages systems biology for clinical application |  | |  |
| **21** | information and communication technologies, and the conceptual framework of complex system studies | to understand the critical points of health maintanance and prevent disease development  to aid understanding of the nonpulmonary determinants of heterogeneity in the common and debiliating condition of chronic obstructive pulmonary disease (COPD) | |  |
| **23** | shedding light in multiple research scenarios, ultimately leading to the practical result of uncovering novel dynamic interaction networks that are critical  clinical and molecular know-how  scrutinizing overall molecular network interactions, rather than individual molecules | identify clinically important molecular targets for diagnostic and therapeutic measures against such a condition  influencing the course of medical conditions  to produce exquisite datasets that are employed to generate pathway models and treatment and will hopefully directly contribute to stratified medicine en-route to personalized healthcare  The application of systems biology  for more effective and clinically applicable research outcomes | |  |
| **26** |  | links disease-associated genes to the phenotypes they produce, a key goal within systems medicine. | |  |
| **28** | an implementation of Systems Biology (SB) in the Medical disciplines  implies the establishment of a connection between a molecular-centered to a patient-centered world, through an organ-centered intermediate layer. This mapping (Figure 1) requires the extensive use of computational tools such as statistical, mathematical and bioinformatical techniques  through a shifting paradigm, starting from a cellular, toward a patient centered framework. According to this vision, the three pillars of SM are Biomedical hypotheses, experimental data, mainly achieved by Omics technologies and tailored computational, statistical and modeling tools. The three SM pillars are highly interconnected, and their balancing is crucial  is deeply related to complex networks: it involves a systemic view of the organism where the various building elements are considered in their interplay | a particular attention to clinical applications, including clinical Bioinformatics and the discrimination of pathological states and related morbidities and comorbidities  extension of Systems Biology (SB) to Clinical-Epidemiological disciplines | |  |
| **29** |  | identify new patterns in the pathogenesis, diagnosis and prognosis of chronic diseases | |  |
| **30** | with all of a patient’s medical data being computationally integrated and accessible  to functionally interpret omics and big data  incorporating a range of personalized data including genomic, epigenetic, environmental, lifestyle and medical history  To achieve these goals, precision medicine aims to develop computational models that integrate data and knowledge from both clinic and basic research to gain a mechanistic understanding of disease | to achieve a shift to future healthcare systems with a more proactive and predictive approach to medicine, where the emphasis is on disease prevention rather than the treatment of symptoms. The individualization of treatment for each patient will be at the centre of this approach  to facilitate their application [of omics and big data] to healthcare provision  the aim is to treat every patient as an individual case  inform rational therapy design for each patient  thereby facilitating personalized treatment decisions | |  |
| **31** | Systems medicine analyzes the dynamic data cloud that surrounds each patient and uses this  rely on data as the primary modeling material, not knowledge  which purports to design multiscale mathematical disease models | to derive “actionable possibilities” that can improve wellness or avoid disease for each patient.  predictive, preventive, personalized, and participatory medicine  developing new diagnostic and therapeutic reagents to terminate a disease trajectory for each individual early, returning them to wellness  aims at predicting the course of a disease in a given patient and how far it can be altered by available therapies  the prediction of benefit–risk for a single subject, a group, or a population | |  |
| **34** | is concerned with the network of molecular interactions that define biological processes. Additionally, disease states are viewed as a perturbation of these molecular networks | the application of systems biology to medicine  concerned with the complex network interplay of a biological unit and represents injury and illness as a perturbation to the network | |  |
| **35** | amalgamates systems biology techniques with medical treatment decision-making, where information from many biological measurements is combined and analysed for complex patterns of change. |  | |  |
| **36** | Systems medicine is not simply the application of systems biology in medicine; rather, it is the logical next step and necessary extension of systems biology with more emphasis on clinically relevant applications. Building on the success of systems biology, systems medicine is defined as an emerging discipline that integrates comprehensively computational modeling, ’omics data, clinical data, and environmental factors  utilizes all types of nonlinear information | aims to offer new approaches for addressing the diagnosis and treatment of major human diseases uniquely, effectively, and with personalized precision  to model and predict disease expression (the pathophenome). Systems medicine integrates basic research and clinical practice, and emphasizes translational and clinical research  highly comprehensive and integrative  aims to offer a powerful set of methodologies to improve our understanding of disease pathogenesis and to design personalized therapies to address the complexity of human diseases | |  |
| **37** | where traditional model-driven experiments are informed by data-driven models in an iterative manner | the clinical application of Systems Biology approaches to medicine | |  |
| **45** | molecular fingerprints resulting from biological networks perturbed by the disease will be used  the use of network-based models of biological process combined with the information on the patient, mainly of molecular origin  integrates physiopathology, network biology and molecular variations  through stratification of patients and diseases | to detect and stratify various pathological conditions  providing novel insights into the mechanisms of various diseases, such as diabetes and obesity, overcoming the current limitations of disease complexity | |  |
| **47** | data are collected from all the components of the immune system, analyzed and integrated | 1. to generate a mathematical model that describes or predicts the response of the system to individual perturbations 2. interdisciplinary approach that systematically describes the complex interactions between all parts of a biological system, with a view to elucidating new biological rules capable of predicting the behavior of the biological system | |  |
| **57** | embraces this paradigm [Systems Biology] | adaptation and extension of Systems Biology | |  |
| **58** | 1. taking advantage and emphasizing information and tools made available by the greatest possible spectrum of scientific disciplines 2. standardization, information, integration, monitoring and personalization | aimed at improving risk prediction and individual treatment respecting ethical and legal requirements | |  |
| **59** | application of systems biology to medical research and practice |  | |  |
| **60** | analyzing the interactions between the different components within one organizational level (genome, transcriptome, proteome), and then between the different levels |  | |  |
| **61** | combining omics with bioinformatics, as well as functional and clinical studies | To find novel diagnostic markers  to find novel therapeutic targets | |  |
| **71** | representing all the available knowledge on the disease of interest with a mathematical symbolism allowing generation and testing of hypotheses through computational simulation and experimental validation | innovative approach to complex diseases understanding and drug discovery | |  |
| **77** | integrate a variety of data at all relevant levels of cellular organisation with clinical and patientreported disease markers, using the power of computational and mathematical modelling | enable the understanding of the mechanisms, prognosis, diagnosis and treatment of disease | |  |
| **78** | applies the perspective of SB [Systems Biology] to the study of disease mechanisms | improving the diagnostic process, disease management, and outcomes | |  |
| **83** | 1. network-based approach to analysis of high-throughput and routine clinical data to predict disease mechanisms to diagnoses and treatments 2. interdisciplinary approach that integrates research data and clinical practice and others view it as fusion of systems biology and bioinformatics with a focus on disease and the clinic 3. high-precision, mathematical model of variables from different genomic layers that relate to clinical outcomes such as treatment response | 1. gain a translational understanding of the complex mechanisms underlying common diseases 2. to address the problem that a disease is rarely caused by malfunction of one individual gene product, but instead depends on multiple gene products that interact in a complex network 3. natural extension of, or is complementary to, current models for clinical decision-making | |  |
| **84** | 1. interdisciplinary approach that integrates data from basic research and clinical practice 2. close integration of data generation with mathematical modeling 3. development of concepts, methods and tools that support the integration of organizational levels | 1. improve our understanding and treatment of diseases 2. further development of systems biology and bioinformatics towards applications of clinical relevance 3. to derive a mechanistic understanding of pathologies, prophylaxy and support of therapy optimization 4. develop interfaces between the computational and mathematical frameworks used in systems medicine | |  |
| **86** | 1. interdisciplinary effort 2. applies the tools and concepts from systems biology and addresses complexity in two key ways. First, systems medicine uses molecular diagnostics to stratify patients and diseases 3. applying a network-level view of disease 4. identifying important functional and regulatory modules within these networks 5. by analyzing and targeting hubs—the most highly interconnected nodes—within these regulatory networks, and enzymatic activity in metabolic networks | 1. [Systems Biology] integrate molecular, cellular, tissue, organ, and organism levels of function into computational models that facilitate the identification of general principles. Systems medicine adds a disease focus. 2. to better characterize and understand disease complexity 3. to create disease networks 4. overcome current limitations in drug discovery 5. network-based approaches will be able to explore the effects of various drugs in mathematical models | |  |
| **88** |  | a better understanding of cellular and molecular networks as key pathogenic elements of human diseases | |  |
| **91** | 1. iterative and reciprocal feedback between data-driven computational and mathematical models as well as model-driven translational and clinical investigations 2. specific but large and static data sets acquired across multiple modalities are used | 1. implementation of Systems Biology approaches in medical concepts, research and practice 2. to construct computational models for the dynamic prediction of disease progression or response to treatment at a personal level | |  |
| **92** |  | application of the systems biology approach to disease-focused or clinically relevant research problems | |  |
| **96** |  | 1. provide a conceptual and theoretical framework 2. practical goal is to provide physicians the tools necessary for harnessing the rapid advances in basic biomedical science into their routine clinical arsenal 3. to provide the tools to take into account the complexity of the human body and disease in the everyday medical practice | |  |
| **98** | based on theoretical methods and high-throughput “omics” data | to answer clinical questions | |  |
| **99** | 1. statistical and computational analysis of metabolic, phenotypic, and physiological data 2. application of computational and statistical approaches to support clinical decisions | 1. clinical decision making is supported 2. integrated study of system level metabolic, phenotypic, and physiological changes in response to disease processes or therapies | |  |
| **101** |  | application of systems biology in a clinical context | |  |
| **103** |  | not the mere translation of the terminology from computer and life sciences to the medical field | |  |
| **104** | 1. tools for data integration 2. sophisticated measurement of molecular moieties | 1. dedicated to deciphering the control mechanisms existing within model organisms such as yeast 2. Systems models of disease | |  |
| **105** | united genomics and genetics through family genomics | more readily identify disease genes | |  |
| **106** | different specific complex factors are important in disease management and that these factors need to be incorporated in some meaningful way | treatment selection and delivery | |  |
| **107** | standardization of data | 1. application of a systems biology approach in medical research and clinical practice 2. to intervene at an early stage to prevent the occurrence and reduce the suffering of the effects of disease, in contrast to chiefly targeting reactive measures only following the occurrence of disease 3. embraces and includes programs such as P4 medicine and personalized medicine 4. data integration from omics to the clinic | |  |
| **108** | integrating experiments in iterative cycles with computational modeling, simulation, and theory | 1. extension of systems biology 2. carries this approach forward into a disease-oriented era | |  |
| **118** |  | application of systems biology approaches to medical research and medical practice | |  |
| **119** |  | application of systems biology to the challenge of human disease | |  |
| **121** | 1. identifying all the components of a system, establishing their interactions and assessing their dynamics – both temporal and spatial – as related to their functions 2. utilizes all types of biological information – DNA, RNA, protein, metabolites, small molecules, interactions, cells, organs, individuals, social networks and external environmental signals – integrating them | 1. a systems approach to health and disease 2. to lead to predictive and actionable models for health and disease | |  |
| **122** | the fully implementation of which requires marrying basic and clinical researches through advanced systems thinking and the employment of high-throughput technologies in genomics, proteomics, nanofluidics, single-cell analysis, and computation strategies in a highly-orchestrated discipline | | predictive, preventive, personalized, and participatory (P4) medicine  translational systems medicine | |
| **124** | using the power of computational and mathematical modeling | | to integrate a variety of biological/medical data on all relevant levels of cellular organization,  to enable an understanding of the pathophysiological mechanisms, prognosis, diagnosis and treatment of disease  to represent signs and symptoms of diseases in multi-level computational models of cells, tissues, organs, organ systems and even organisms  the application of systems biology approaches to medical research and medical practice  molecular) systems biology in medicine | |
| **125** | using knowledge of their molecular components   must exploit more limited data sets, arising from multiple open-ended investigations upon highly heterogeneous patient populations in conjunction with vast amounts of poorly correlated published results. Hence, systems medicine must proceed on the basis of existing, highly heterogeneous data and not on the basis of homogeneous datasets arising from specifically targeted investigations. | | to reconstruct organs and organisms  to determine clinical behaviours and interventions   a holistic approach to medicine (systems medicine), that could benefit patients and society | |
| **130** | companion molecular diagnostics for personalized therapy  the mounting influx of global quantitative data from both wellness and diseases,  which requires new strategies, both scientific and organizational | | is shaping up a transformational paradigm in medicine we termed predictive, preventive, personalized, and participatory (P4) medicine  to enable bringing this revolution in medicine to patients and to the healthcare system. | |
| **131** | by determining the links between genotypes, phenotypes and environmental factors (e.g. diet and exposure to toxins)  by analysing its different constituents | | The reconstruction of such biological network models, the combination of these models with omics data and their application to specific medical questions are often referred to as systems medicine.  a better understanding of the structure and function of the human genome and its associations  helps to understand the behaviour of the human body at all levels of organization  it offers the prospects of modelling complex diseases, establishing novel diagnostic and therapeutic techniques [16], identifying new drug targets [17], developing a system-orientated drug design strategy [18] and eventually achieving effective personalized medicine | |
| **134** | emphasizes the role of systems biology in medical/clinical applications  With the advent of new technologies, the “omics” explosion (i.e., next generation sequencing) and the induced changes from data-poor to data-rich applications (for instance related to high-content imaging, physiology, and structural biology) have established the necessity of a systems approach (Noble, 2008  Systems medicine represents a mosaic of distinct and interconnected micro-systems  originated by a variety of information sources and consequently characterized. | | not to be caught in the data deluge.  allowing to infer the macro-systems dynamics and produce elements of synthesis such as signatures (Hood and Friend, 2011; Sung et al., 2012) and profiles | |
| **141** | leverages complex computational tools and high-dimensional data  the effective use of petabytes of data, which necessitates the development of both new types of tools and a new type of physician—one with a grasp of modern computational sciences, “omics” technologies, and a systems approach to the practice of medicine  systems biology | | an application of systems biology approaches to biomedical problems in the clinical setting,  to derive personalized assessments of disease risk  more effective individualized diagnosis, prognosis, and treatment options  the foundation for a practice of systems medicine in the future that will be predictive, personalized, preventive, and participatory | |
| **142** |  | | Systems or ‘P4’ medicine offers a grand vision for achieving better population health. The four Ps - predictive, preventive, personalized and participatory - invoke a patient-centered approach that prioritizes health promotion over disease treatment | |
| **143** | This proposed holistic strategy involves comprehensive patient-centered integrated care and multi-scale, multi-modal and multi-level systems approaches  Rather than studying each disease individually, it will take into account their intertwined gene-environment, socio-economic interactions and co-morbidities that lead to individual-specific complex phenotypes.  based on a robust and extensive knowledge management infrastructure that contains individual patient information.  It will be supported by strategic partnerships involving all stakeholders, including general practitioners associated with patient-centered care. This systems medicine strategy, which will take a holistic approach to disease  It uses the power of computational and mathematical modeling].  takes a holistic view of health and disease  through integrated care using multidisciplinary and teamwork approaches centered in primary and community | | to tackle NCDs as a common group of diseases.  for predictive, preventive, personalized and participatory (P4) medicine  designed to allow the results to be used globally, taking into account the needs and specificities of local economies and health systems.  Systems medicine is the application of systems biology to medical research and practice [54,55].  to integrate a variety of data at all relevant levels of cellular organization with clinical and patient-reported disease markers.  to enable understanding of the mechanisms, prognosis, diagnosis and treatment of disease [56]  It involves a transition to predictive, preventive, personalized and participatory (P4) medicine, which is a shift from reactive to prospective medicine that extends far beyond what is usually covered by the term personalized medicine  to tackle all components of the complexity of NCDs so as to understand these various phenotypes and hence enable prevention (Box 2), control through health promotion [50] and personalized medicine [51], and an efficient use of health service resources [52] | |
| **146** | Understanding the unique events in an individual’s life as influencing the development of illness and disease appears to be the key to what is emerging under the names of ‘personalized medicine’ and ‘systems medicine’.  Personalized medicine presupposes systems biology and complexity sciences, […] | |  | |
| **148** | Systems biology and [!?] medicine focuses on deciphering mechanisms at multiple levels, reconstructing networks in cells, tissues and organs, measuring and predicting phenotypes, building quantitative models that describe and simulate normal and pathological physiological functions, and then testing the validity of these models and predictions experimentally. | |  | |
| **149** |  | | The main goal of systems medicine is to provide predictive models of the pathophysiology of complex diseases as well as define healthy states. | |
| **150** | exploration of tumor microenvironment2,15 and of a more global approach to link individual tumors with their multiple host variables, including heritable causal mutations, environmental exposures and lifestyle, | |  | |
| **153** | the elucidation of drug targets, an important step in the search for new drugs or novel targets for existing drugs. Incorporating multiple biological information sources is of essence | | Understanding drugs and their modes of action  for improving the accuracy of drug target prediction | |
| **157** | applicable methodology tool, systems biology. | | new strategies capable of integrating all known information about the elements that make up the reality called asthma, thus offering a detailed mapping of its complexity. | |
| **158** | Systems medicine, the translational science counterpart to basic science’s systems biology, is the interface at which these tools may be constructed  […] systems medicine is the coupling of systems science with medical treatment decision-making (Auffray et al. 2009). | | […] systems medicine, as a translationally relevant extension of systems biology (Auffray et al. 2009). | |
| **160** | systems medicine approaches focus on the dynamic interactions among multiple factors that affect complex diseases, such as diabetes, coronary artery disease and cancers1. The increasing availability of powerful high-throughput technologies, computational tools and integrated knowledge bases, has made it possible to establish new links between genes, biologic functions and human diseases, providing the hallmarks of systems medicine, including signatures of pathology biology, and links to clinical research and drug discovery2.  Holistic systems biology methodologies  through the construction of integrated biomolecular networks3. | | promise to provide the foundation for such prospective medicine | |
| **162** | The knowledge of network dynamics through in vitro experimental perturbation and modeling allows us to determine the state of the networks, to identify molecular correlates, and.  The transformation in biology through systems biology  The central premise of systems medicine is that clinically detectable molecular fingerprints resulting from disease-perturbed biological networks will be used to detect and stratify various pathological conditions. Disease associated molecular fingerprints will eventually be used to group individuals into sub-populations based on variations in genetic makeup of the population that affects disease progression. The key to this revolution lies in harnessing the power of network models of core biological processes learned through systems biology methods, combined with vast amounts of diverse molecular information generated from patient samples.  depends on our ability to: 1) precisely infer network state from the results of assessing the levels  of a panel of informative, diagnostic biomarkers in the blood, and 2) specifically manipulate a network to avoid or revert the pathology.  the application of our understanding of the integrated dynamical responses of various molecular networks that determine the critical states of the body.  the therapeutic component of systems medicine then, in which we infer network states from biomarker measurements | | to derive new disease treatment approaches to reverse the pathology or prevent its progress into a more severe state through the manipulation of network states  This general approach, including diagnostics and therapeutics, is becoming known as systems medicine.  will enable a new medical discipline – systems medicine  intervene to halt and reverse the networks progress into an undesired state | |
| **164** | the application of systems biology | | to the prevention of, understanding and modulation of, and recovery from developmental disorders and pathologic processes in human health  systems medicine emphasizes that the essential purpose and relevance of models is translational, aimed at diagnostic, predictive, and therapeutic applications.  systems medicine aims to discover and select the key factors at each level and integrate them into models of translational relevance, which include measurable readouts and clinical predictions. | |
| **165** | incorporates the complex biochemical, physiological, and environmental interactions that sustain living organisms.  incorporates interactions between all components of health and disease.  A key feature of systems medicine is that existing networks, through dynamic (time-dependent) interactions, manifest “emergent properties” that define the whole and that these properties are not simply the sum of the features of its component parts. | |  | |
| **170** | by integrating all levels of quantitative functional, structural, and morphological information into a coherent model.  It investigates the physiological network of diseases from gene to organ systems | | tries to understand perturbed physiological systems and complex pathologies in their entirety  an integrative and systemic approach for the diagnosis, therapy, and prevention of diseases [47  with four main goals — predictive, preventive, personalized, and participative medicine (P4 medicine). | |
| **171** | via an integrative approach that includes clinical examinations, experimental modeling and in-silico simulation.  by integrating all levels of quantitative functional, structural and morphological information into a coherent model. | | to understand perturbed physiological systems and complex pathologies in their entirety  geared towards obtaining clinical impact with both diagnostic and therapeutic end points. | |
| **125** | Systems medicine is an emerging concept that acknowledges the complexity of a multitude of non-linear interactions among molecular and physiological variables.  Under this new paradigm, rather than a collection of symptoms, diseases are seen as the product of deviations from a robust steady state compatible with life.  the incorporation of mathematics and physics to the more classical arsenal of physiology and molecular biology with which physicians are trained today. | |  | |
