# Supplementary material for: Applying systems biology to biomedical research and health care: a précising definition of systems medicine
Source: BMC Health Serv Res. 2017 Nov 21;17:761. doi: 10.1186/s12913-017-2688-z (PMC5698952; doi:10.1186/s12913-017-2688-z)
Supplement: Supplementary file 12 — Application of the six adequacy criteria to the ends of Systems Medicine (DOCX 32 kb) [file 12913_2017_2688_MOESM12_ESM.docx]

**Application of the six adequacy criteria to the ends of Systems Medicine**(Green shaded text passages are not eligible for the definition. For each text passage deemed not eligible we give a short blue colored explanation concerning what criterion is not met and why it is not met)

| **Number** | **Ends** |
| --- | --- |
| **3** | enables the personalization of diagnosis, prognosis and treatment  helps to re-define clinical phenotypes  to discover new diagnostic and prognostic biomarkers  to guide the design of new clinical trials  too vague: it is not clear, what “guiding a trial design” means and what might be new of the “new clinical trials”. (Criterion 5) |
| **8** | accurately predict sensitivity of an individual tumor to a drug or drug combination  to generate genomics informed personalized therapeutic regimes with higher efficacy  assist in designing personalized cancer therapy treatments with expected effectiveness significantly higher than current standard of care approaches |
| **9** | to deliver P4 and precision medicine in the future. This will enable introduction of individualized tailored prevention and/or treatment strategies |
| **13** |  |
| **21** | to understand the critical points of health maintanance and prevent disease development  to aid understanding of the nonpulmonary determinants of heterogeneity in the common and debiliating condition of chronic obstructive pulmonary disease (COPD) |
| **23** | influencing the course of medical conditions  identify clinically important molecular targets for diagnostic and therapeutic measures against such a condition  to produce exquisite datasets that are employed to generate pathway models and treatment and will hopefully directly contribute to stratified medicine en-route to personalized healthcare  The application of systems biology  for more effective and clinically applicable research outcomes  too vague: the meaning and comparative reference of the expression “more effective research outcomes” remains unspecified (Criterion 5) |
| **26** | links disease-associated genes to the phenotypes they produce, a key goal within systems medicine. |
| **28** | a particular attention to clinical applications, including clinical Bioinformatics and the discrimination of pathological states and related morbidities and comorbidities  too vague: it is unclear what the “particular attention to clinical applications” concretely consists in and what it means in real world (Criterion 5)  extension of Systems Biology (SB) to Clinical-Epidemiological disciplines  too vague: “extension of Systems Biology” is not further specified and has no clear meaning (Criterion 5) |
| **29** | identify new patterns in the pathogenesis, diagnosis and prognosis of chronic diseases |
| **30** | to achieve a shift to future healthcare systems with a more proactive and predictive approach to medicine, where the emphasis is on disease prevention rather than the treatment of symptoms. The individualization of treatment for each patient will be at the centre of this approach  facilitate their application [of omics and big data] to healthcare provision  the aim is to treat every patient as an individual case  inform rational therapy design for each patient  thereby facilitating personalized treatment decisions |
| **31** | to derive “actionable possibilities” that can improve wellness or avoid disease for each patient.  predictive, preventive, personalized, and participatory medicine  developing new diagnostic and therapeutic reagents to terminate a disease trajectory for each individual early, returning them to wellness  aims at predicting the course of a disease in a given patient and how far it can be altered by available therapies  the prediction of benefit–risk for a single subject, a group, or a population |
| **34** | the application of systems biology to medicine  concerned with the complex network interplay of a biological unit and represents injury and illness as a perturbation to the network |
| **35** |  |
| **36** | aims to offer new approaches for addressing the diagnosis and treatment of major human diseases uniquely, effectively, and with personalized precision  to model and predict disease expression (the pathophenome). Systems medicine integrates basic research and clinical practice, and emphasizes translational and clinical research  highly comprehensive and integrative  aims to offer a powerful set of methodologies to improve our understanding of disease pathogenesis and to design personalized therapies to address the complexity of human diseases |
| **37** | the clinical application of Systems Biology approaches to medicine |
| **45** | to detect and stratify various pathological conditions  providing novel insights into the mechanisms of various diseases, such as diabetes and obesity, overcoming the current limitations of disease complexity |
| **47** | 1. to generate a mathematical model that describes or predicts the response of the system to individual perturbations 2. interdisciplinary approach that systematically describes the complex interactions between all parts of a biological system, with a view to elucidating new biological rules capable of predicting the behavior of the biological system |
| **56** | adaptation and extension of Systems Biology  too vague: not clear what “extension” is supposed to mean here (Criterion 5) |
| **57** | aimed at improving risk prediction and individual treatment respecting ethical and legal requirements  too vague: unclear what this specification may mean as and add to an end of Systems Medicine (Criterion 5) |
| **61** | find novel diagnostic markers and therapeutic targets |
| **71** | innovative approach to complex diseases understanding and drug discovery |
| **77** | enable the understanding of the mechanisms, prognosis, diagnosis and treatment of disease |
| **78** | improving the diagnostic process, disease management, and outcomes |
| **83** | 1. gain a translational understanding of the complex mechanisms underlying common diseases 2. to address the problem that a disease is rarely caused by malfunction of one individual gene product, but instead depends on multiple gene products that interact in a complex network 3. natural extension of, or is complementary to, current models for clinical decision-making   vague terms: “natural extension”  “complementary to...” are unclear within that context (Criterion 5) |
| **84** | 1. improve our understanding and treatment of diseases 2. further development of systems biology and bioinformatics towards applications of clinical relevance 3. to derive a mechanistic understanding of pathologies, prophylaxy and support of therapy optimization 4. develop interfaces between the computational and mathematical frameworks used in systems medicine   circularity: the definiendum is part of the definiens (Criterion 2) |
| **86** | 1. [Systems Biology] integrate molecular, cellular, tissue, organ, and organism levels of function into computational models that facilitate the identification of general principles. Systems medicine adds a disease focus. 2. to better characterize and understand disease complexity 3. to create disease networks 4. overcome current limitations in drug discovery 5. network-based approaches will be able to explore the effects of various drugs in mathematical models |
| **88** | a better understanding of cellular and molecular networks as key pathogenic elements of human diseases |
| **91** | 1. implementation of Systems Biology approaches in medical concepts, research and practice 2. to construct computational models for the dynamic prediction of disease progression or response to treatment at a personal level |
| **92** | application of the systems biology approach to disease-focused or clinically relevant research problems |
| **96** | 1. provide a conceptual and theoretical framework   too vague; it is not clear what providing a conceptual and theoretical framework might precisely mean as end within the medical context (Criterion 5)   1. practical goal is to provide physicians the tools necessary for harnessing the rapid advances in basic biomedical science into their routine clinical arsenal 2. to provide the tools to take into account the complexity of the human body and disease in the everyday medical practice |
| **98** | to answer clinical questions  Too vague; the precise meaning of the term “clinical questions” is not clear (Criterion 5) |
| **99** | 1. clinical decision making is supported 2. integrated study of system level metabolic, phenotypic, and physiological changes in response to disease processes or therapies |
| **101** | application of systems biology in a clinical context |
| **103** | not the mere translation of the terminology from computer and life sciences to the medical field1  too vague for a definition since it only tells us what systems medicine is *not* ; (Criterion 5) |
| **104** | 1. dedicated to deciphering the control mechanisms existing within model organisms such as yeast 2. Systems models of disease |
| **105** | more readily identify disease genes |
| **106** | treatment selection and delivery |
| **107** | 1. application of a systems biology approach in medical research and clinical practice 2. to intervene at an early stage to prevent the occurrence and reduce the suffering of the effects of disease, in contrast to chiefly targeting reactive measures only following the occurrence of disease 3. embraces and includes programs such as P4 medicine and personalized medicine     too vague: there are two elements which make the whole too vague: first, it is unclear what the term „programs“ means; second, the syntactical element „such as“ is not appropriate for a definition for it explains only through reference to an example and not in general terms (Criterion 5)   1. data integration from omics to the clinic |
| **108** | 1. extension of systems biology   too vague; it is not clear what extension means in that context (Criterion 5)   1. carries this approach forward into a disease-oriented era   too vague; it is unclear what “disease-oriented era” means (as opposed to what?) (Criterion 5) |
| **118** | application of systems biology approaches to medical research and medical practice |
| **119** | application of systems biology to the challenge of human disease |
| **121** | 1. a systems approach to health and disease 2. to lead to predictive and actionable models for health and disease |
| **122** | predictive, preventive, personalized, and participatory (P4) medicine  translational systems medicine  circularity: the definiendum appears in the definiens (Criterion 2) |
| **124** | to integrate a variety of biological/medical data on all relevant levels of cellular organization,  to enable an understanding of the pathophysiological mechanisms, prognosis, diagnosis and treatment of disease  to represent signs and symptoms of diseases in multi-level computational models of cells, tissues, organs, organ systems and even organisms  the application of systems biology approaches to medical research and medical practice  molecular) systems biology in medicine |
| **125** | to reconstruct organs and organisms to determine clinical behaviours and interventions   a holistic approach to medicine (systems medicine), that could benefit patients and society  too vague: it is expressed in very vague terms, does not refer to any concrete thing or meaning and, additionally, is expressed only in terms of possibility (“could”) (Criterion 5) |
| **130** | is shaping up a transformational paradigm in medicine we termed predictive, preventive, personalized, and participatory (P4) medicine  to enable bringing this revolution in medicine to patients and to the healthcare system. |
| **131** | The reconstruction of such biological network models, the combination of these models with omics data and their application to specific medical questions are often referred to as systems medicine.  Too vague: the meaning of „specific medical questions“ remains completey vague and can not be derived. No additional information or meaning to the mere term “Systems Medicine” is added (Criterion 5)  a better understanding of the structure and function of the human genome and its associations  helps to understand the behaviour of the human body at all levels of organization  , it offers the prospects of modelling complex diseases, establishing novel diagnostic and therapeutic techniques [16], identifying new drug targets [17], developing a system-orientated drug design strategy [18]  circularity: an essential part of the definiendum (the term “system”) is part of the definiens (in “system-approach”) (Criterion 2)  and eventually achieving effective personalized medicine |
| **134** | not to be caught in the data deluge  too vague: a merely negative sentence just telling us what Systems Medicine does not consist in (Criterion 5)  allowing to infer the macro-systems dynamics and produce elements of synthesis such as signatures (Hood and Friend, 2011; Sung et al., 2012) and profiles |
| **141** | an application of systems biology approaches to biomedical problems in the clinical setting,  to derive personalized assessments of disease risk  more effective individualized diagnosis, prognosis, and treatment options  the foundation for a practice of systems medicine in the future that will be predictive, personalized, preventive, and participatory |
| **142** | Systems or ‘P4’ medicine offers a grand vision for achieving better population health.  The four Ps - predictive, preventive, personalized and participatory - invoke a patient-centered approach that prioritizes health promotion over disease treatment |
| **143** | to tackle NCDs as a common group of diseases.  for predictive, preventive, personalized and participatory (P4) medicine  designed to allow the results to be used globally, taking into account the needs and specificities of local economies and health systems.  Too vague; it is a combination of several vague and highly general “big” terms; we can not derive any concrete meaning from this with regard to potential end of Systems Medicine (Criterion 5)  Systems medicine is the application of systems biology to medical research and practice [54, 55].  to integrate a variety of data at all relevant levels of cellular organization with clinical and patient-reported disease markers.  to enable understanding of the mechanisms, prognosis, diagnosis and treatment of disease [56]  It involves a transition to predictive, preventive, personalized and participatory (P4) medicine, which is a shift from reactive to prospective medicine that extends far beyond what is usually covered by the term personalized medicine  to tackle all components of the complexity of NCDs so as to understand these various phenotypes and hence enable prevention (Box 2), control through health promotion [50] and personalized medicine [51], and an efficient use of health service resources [52]  Too vague; we do not know what goods or criteria are used to define efficiency; There remains the open question: efficiency in/of what? (Criterion 5) |
| **146** |  |
| **148** |  |
| **149** | The main goal of systems medicine is to provide predictive models of the pathophysiology of complex diseases as well as define healthy states. |
| **150** |  |
| **153** | Understanding drugs and their modes of action 1  for improving the accuracy of drug target prediction |
| **157** | new strategies capable of integrating all known information about the elements that make up the reality called asthma, thus offering a detailed mapping of its complexity. |
| **158** | […] systems medicine, as a translationally relevant extension of systems biology (Auffray et al. 2009).  Too vague; it is unclear what is meant by “translationally releveant extension” (Criterion 5)  […] systems medicine is the coupling of systems science with medical treatment decision-making (Auffray et al. 2009). |
| **160** | promise to provide the foundation for such prospective medicine |
| **162** | to derive new disease treatment approaches to reverse the pathology or prevent its progress into a more severe state through the manipulation of network states  This general approach, including diagnostics and therapeutics, is becoming known as systems medicine.  Too vague: it is unclear what “this approach” really means and whether diagnostics and therapeutics are all that is included. (Criterion 5)  will enable a new medical discipline – systems medicine  intervene to halt and reverse the networks progress into an undesired state |
| **164** | to the prevention of, understanding and modulation of, and recovery from developmental disorders and pathologic processes in human health  systems medicine emphasizes that the essential purpose and relevance of models is translational, aimed at diagnostic, predictive, and therapeutic applications.  too vague: it is unclear what “emphasizes” might mean (Criterion 5)  systems medicine aims to discover and select the key factors at each level and integrate them into models of translational relevance, which include measurable readouts and clinical predictions. |
| **165** |  |
| **170** | tries to understand perturbed physiological systems and complex pathologies in their entirety  an integrative and systemic approach for the diagnosis, therapy, and prevention of diseases [47]  with four main goals — predictive, preventive, personalized, and participative medicine (P4 medicine). |
| **171** | to understand perturbed physiological systems and complex pathologies in their entirety  geared towards obtaining clinical impact with both diagnostic and therapeutic end points. |
