# Supplementary material for: Applying systems biology to biomedical research and health care: a précising definition of systems medicine
Source: BMC Health Serv Res. 2017 Nov 21;17:761. doi: 10.1186/s12913-017-2688-z (PMC5698952; doi:10.1186/s12913-017-2688-z)
Supplement: Supplementary file 13 — Inductive assigning of remaining ends to preliminary categories (DOCX 30 kb) [file 12913_2017_2688_MOESM13_ESM.docx]

**Inductive assigning of remaining ends to preliminary categories**

**End i) Improving Participation**

(only mentioned in regards to P4 Medicine: predictive, preventive, personalized, and participatory (P4) medicine)

**End ii) Improving Prediction**

describes or predicts the response of the system to individual perturbations

predicting the behavior of the biological system

improving risk prediction

dynamic prediction of disease progression or response to treatment at a personal level

to derive personalized assessments of disease risk

The main goal of systems medicine is to provide predictive models of the pathophysiology of complex diseases

for improving the accuracy of drug target prediction

to achieve a shift to future healthcare systems with a more proactive and predictive approach to medicine

aims at predicting the course of a disease in a given patient and how far it can be altered by available therapies

the prediction of benefit–risk for a single subject, a group, or a population

predict disease expression (the pathophenome)

to lead to predictive and actionable models

accurately predict sensitivity of an individual tumor to a drug or drug combination

**End iii) Improving Prevention**

to intervene at an early stage to prevent the occurrence and reduce the suffering of the effects of disease, in contrast to chiefly targeting reactive measures only following the occurrence of disease

which is a shift from reactive to prospective medicine that extends far beyond what is usually covered by the term personalized medicine

promise to provide the foundation for such prospective medicine

or prevent its progress into a more severe state through the manipulation of network states

and intervene to halt and reverse the networks progress into an undesired state,

to the prevention of (…) developmental disorders and pathologic processes in human health

where the emphasis is on disease prevention rather than the treatment of symptoms.

identify new patterns in the pathogenesis, diagnosis and prognosis of chronic diseases

to derive prophylaxy

**End iv) Improving Stratification**

eventually achieving effective personalized medicine

more effective individualized diagnosis, prognosis, and treatment options

enables the personalization of diagnosis, prognosis and treatment

and prevent disease development

The individualization of treatment for each patient will be at the centre of this approach

the aim is to treat every patient as an individual case

inform rational therapy design for each patient

thereby facilitating personalized treatment decisions

developing new diagnostic and therapeutic reagents to terminate a disease trajectory for each individual early, returning them to wellness

and with personalized precision

and to design personalized therapies to address the complexity of human diseases

to detect and stratify various pathological conditions

enable personalized medicine

to produce exquisite datasets that will hopefully directly contribute to stratified medicine en-route to personalized healthcare

to generate genomics informed personalized therapeutic regimes with higher efficacy

assist in designing personalized cancer therapy treatments with expected effectiveness significantly higher than current standard of care approaches

**End iv) Improving Treatment**

aimed at improving individual treatment

improving disease management, and outcomes

improve our and treatment of diseases

and support of therapy optimization

to provide the tools to take into account the complexity of the human body and disease in the everyday medical practice

to provide physicians the tools necessary for harnessing the rapid advances in basic biomedical science into their routine clinical arsenal

determine clinical behaviours and interventions

establishing novel therapeutic techniques

to derive new disease treatment approaches to reverse the pathology

recovery from developmental disorders and pathologic processes in human health

aims to offer new approaches for addressing treatment of major human diseases uniquely, effectively

clinical decision making is supported

treatment selection and delivery

geared towards obtaining clinical impact with both diagnostic and therapeutic end points

intervene to halt and reverse the networks progress into an undesired state

influencing the course of medical conditions

assist in designing personalized cancer therapy treatments with expected effectiveness significantly higher than current standard of care approaches

To tackle NCDs as a common group of diseases

to generate genomics informed personalized therapeutic regimes with higher efficacy

**End vi) Improving Diagnostics**

improving the diagnostic process

find novel diagnostic markers

establishing novel diagnostic techniques

aims to offer new approaches for addressing the diagnosis of major human diseases uniquely, effectively

geared towards obtaining clinical impact with both diagnostic and therapeutic end points

identify new patterns in the pathogenesis, diagnosis and prognosis of chronic diseases

**End vii) Modelling**

to represent signs and symptoms of diseases in multi-level computational models of cells, tissues, organs, organ systems and even organisms

to reconstruct organs and organisms

it offers the prospects of modelling complex diseases

integrate them (the key factors at each level) into models of translational relevance, which include measurable readouts and clinical predictions

ultimately leading to the practical result of uncovering novel dynamic interaction networks that are critical for influencing the course of medical conditions

to produce exquisite datasets that are employed to generate pathway models and treatment

to model disease expression (the pathophenome)

**End viii) Improving Understanding of Disease/Pathologies/Health States**

innovative approach to complex diseases understanding

improve our understanding of diseases

to derive a mechanistic understanding of pathologies

to better characterize and understand disease complexity

a better understanding of cellular and molecular networks as key pathogenic elements of human diseases

to lead to actionable models for health and disease

helps to understand the behaviour of the human body at all levels of organization

to tackle all components of the complexity of NCDs so as to understand these various phenotypes

as well as define healthy states

tries to understand perturbed physiological systems and complex pathologies in their entirety

to understand the critical points of health maintanance

to gain a mechanistic understanding of disease

providing novel insights into the mechanisms of various diseases, such as diabetes and obesity, overcoming the current limitations of disease complexity

aims to offer a powerful set of methodologies to improve our understanding of disease pathogenesis

understanding and modulation of developmental disorders and pathologic processes in human health

systems medicine aims to discover and select the key factors at each level

enable the understanding of the mechanisms, prognosis, diagnosis and treatment of disease

gain a translational understanding of the complex mechanisms underlying common diseases

dedicated to deciphering the control mechanisms existing within model organisms such as yeast

to enable an understanding of the pathophysiological mechanisms, prognosis, diagnosis and treatment of disease

to enable understanding of the mechanisms, prognosis, diagnosis and treatment of disease

Understanding drugs and their modes of action

new strategies capable of integrating all known information about the elements that make up the reality called asthma, thus offering a detailed mapping of its complexity

to aid understanding of the nonpulmonary determinants of heterogeneity in the common and debiliating condition of chronic obstructive pulmonary disease (COPD)

identify new patterns in the pathogenesis, diagnosis and prognosis of chronic diseases

**End ix) Innovative Approach to Drug Discovery**

to explore the effects of various drugs

overcome current limitations in drug discovery

Understanding drugs and their modes of action

**End x) Finding Novel Therapeutic Targets**

identifying new drug targets

identify clinically important molecular targets for diagnostic and therapeutic measures against such a condition

**End xi) Discovering New Diagnostic and Prognostic Biomarkers**

more readily identify disease genes

links disease-associated genes to the phenotypes they produce, a key goal within systems medicine

**End xii) Re-Defining Clinical Phenotypes**

helps to re-define clinical phenotypes

links disease-associated genes to the phenotypes they produce

**End xiii) Improving Health Care**

facilitate their application [of omics and big data] to healthcare provision

to achieve a shift to future healthcare systems with a more proactive and predictive approach to medicine

to produce exquisite datasets that will hopefully directly contribute to stratified medicine en-route to personalized healthcare

**End xiv) Achieving better Population Health**

offers a grand vision for achieving better population health
