# Supplementary material for: Applying systems biology to biomedical research and health care: a précising definition of systems medicine
Source: BMC Health Serv Res. 2017 Nov 21;17:761. doi: 10.1186/s12913-017-2688-z (PMC5698952; doi:10.1186/s12913-017-2688-z)
Supplement: Supplementary file 14 — Application of the six adequacy criteria to the means of Systems Medicine (DOCX 30 kb) [file 12913_2017_2688_MOESM14_ESM.docx]

**Application of the six adequacy criteria to the means of Systems Medicine**(Green shaded text passages are not eligible for the definition. For each text passage deemed not eligible we give a short blue colored explanation concerning what criterion is not met and why it is not met)

| **Number** | **Means** |
| --- | --- |
| **3** | combines systems biology and pathophysiological approaches to translational research, integrating various bio-medical tools and using the power of computational and mathematical modelling  using molecular and dynamic parameters |
| **8** | inferred models |
| **9** | incorporating genomic information (genomic medicine) along with appropriate biological and computational tools for data interpretation |
| **13** | leverages systems biology for clinical application |
| **21** | information and communication technologies, and the conceptual framework of complex system studies  circularity: an essential part of the definiendum (the term “systems”) is part of the definiens (in “system studies”) (Criterion 2) |
| **23** | shedding light in multiple research scenarios,  too vague: the terms “shedding light” and “scenarios” are quite unclear (Criterion 5)  ultimately leading to the practical result of uncovering novel dynamic interaction networks that are critical  clinical and molecular know-how  too vague: it is not clear what this might consist in or implies and means. (criterion 5)  scrutinizing overall molecular network interactions, rather than individual molecules |
| **26** |  |
| **28** | an implementation of Systems Biology (SB) in the Medical disciplines  implies the establishment of a connection between a molecular-centered to a patient-centered world, through an organ-centered intermediate layer. This mapping (Figure 1) requires the extensive use of computational tools such as statistical, mathematical and bioinformatical techniques  through a shifting paradigm, starting from a cellular, toward a patient centered framework. According to this vision, the three pillars of SM are Biomedical hypotheses, experimental data, mainly achieved by Omics technologies and tailored computational, statistical and modeling tools. The three SM pillars are highly interconnected, and their balancing is crucial  is deeply related to complex networks: it involves a systemic view of the organism where the various building elements are considered in their interplay  circularity: an essential part of the definiendum (the term “systems”) is part of the definiens (in the form of “systemic”) (Criterion 2) |
| **29** |  |
| **30** | with all of a patient’s medical data being computationally integrated and accessible  to functionally interpret omics and big data  incorporating a range of personalized data including genomic, epigenetic, environmental, lifestyle and medical history  To achieve these goals, precision medicine aims to develop computational models that integrate data and knowledge from both clinic and basic research to gain a mechanistic understanding of disease |
| **31** | Systems medicine analyzes the dynamic data cloud that surrounds each patient and uses this  rely on data as the primary modeling material, not knowledge  too vague: the whole element remains unclear as the supposed opposition between data and knowledge is not made explicit, specified or explained. (criterion 5)  which purports to design multiscale mathematical disease models |
| **34** | is concerned with the network of molecular interactions that define biological processes. Additionally, disease states are viewed as a perturbation of these molecular networks |
| **35** | amalgamates systems biology techniques with medical treatment decision-making, where information from many biological measurements is combined and analysed for complex patterns of change. |
| **36** | Systems medicine is not simply the application of systems biology in medicine; rather, it is the logical next step and necessary extension of systems biology with more emphasis on clinically relevant applications. Building on the success of systems biology, systems medicine is defined as an emerging discipline that integrates comprehensively computational modeling, ’omics data, clinical data, and environmental factors  utilizes all types of nonlinear information  too vague: It is unclear what “nonlinear information” exactly means (criterion 5) |
| **37** | where traditional model-driven experiments are informed by data-driven models in an iterative manner |
| **45** | molecular fingerprints resulting from biological networks perturbed by the disease will be used  the use of network-based models of biological process combined with the information on the patient, mainly of molecular origin  integrates physiopathology, network biology and molecular variations  through stratification of patients and diseases |
| **47** | data are collected from all the components of the immune system, analyzed and integrated |
| **56** | embraces this paradigm [Systems Biology] |
| **57** | 1. taking advantage and emphasizing information and tools made available by the greatest possible spectrum of scientific disciplines   too vague; the expression “greatest possible spectrum of scientific disciplines” is too wide and vague (criterion 5)   1. standardization, information, integration, monitoring and personalization   too vague: an unspecified combination of „big“ terms which does not explain the kind of relation occurring among these terms (criterion 5) |
| **59** | application of systems biology to medical research and practice |
| **60** | analyzing the interactions between the different components within one organizational level (genome, transcriptome, proteome), and then between the different levels |
| **61** | combining omics with bioinformatics, as well as functional and clinical studies |
| **71** | representing all the available knowledge on the disease of interest with a mathematical symbolism allowing generation and testing of hypotheses through computational simulation and experimental validation |
| **77** | integrate a variety of data at all relevant levels of cellular organisation with clinical and patientreported disease markers, using the power of computational and mathematical modelling |
| **78** | applies the perspective of SB [Systems Biology] to the study of disease mechanisms |
| **83** | 1. network-based approach to analysis of high-throughput and routine clinical data to predict disease mechanisms to diagnoses and treatments 2. interdisciplinary approach that integrates research data and clinical practice and others view it as fusion of systems biology and bioinformatics with a focus on disease and the clinic 3. high-precision, mathematical model of variables from different genomic layers that relate to clinical outcomes such as treatment response |
| **84** | 1. interdisciplinary approach that integrates data from basic research and clinical practice 2. close integration of data generation with mathematical modeling 3. development of concepts, methods and tools that support the integration of organizational levels   too vague: it is not clear what “organizational levels” refers to within this semantic context |
| **86** | 1. interdisciplinary effort   too vague since it does not mention the disciplines concretely involved, it is not sufficiently specified to be a means of Systems Medicine (criterion 5)   1. applies the tools and concepts from systems biology and addresses complexity in two key ways. First, systems medicine uses molecular diagnostics to stratify patients and diseases 2. applying a network-level view of disease 3. identifying important functional and regulatory modules within these networks 4. by analyzing and targeting hubs—the most highly interconnected nodes—within these regulatory networks, and enzymatic activity in metabolic networks |
| **88** |  |
| **91** | 1. iterative and reciprocal feedback between data-driven computational and mathematical models as well as model-driven translational and clinical investigations 2. specific but large and static data sets acquired across multiple modalities are used   too vague: the exact meaning of “multiple modalities are used” is very vague and not sufficiently concrete to potentially serve as means of Systems Medicine (criterion 5) |
| **92** |  |
| **96** |  |
| **98** | based on theoretical methods and high-throughput “omics” data  too vague: it is unclear what “theoretical methods” means and in what these methods might consist in (criterion 5) |
| **99** | 1. statistical and computational analysis of metabolic, phenotypic, and physiological data 2. application of computational and statistical approaches to support clinical decisions |
| **101** |  |
| **103** |  |
| **104** | 1. tools for data integration 2. sophisticated measurement of molecular moieties   too vague: the term “sophisticated measurement” is completely unspecified and thus too vague (criterion 5) |
| **105** | united genomics and genetics through family genomics  too vague: the concepts used (“genomics”, ”genetics” and “family genomics”) are not clear, i.e. their differences are not specified, and the reasons why and the way how they shall be united remain open. (criterion 5) |
| **106** | different specific complex factors are important in disease management and that these factors need to be incorporated in some meaningful way |
| **107** | standardization of data |
| **108** | integrating experiments in iterative cycles with computational modeling, simulation, and theory |
| **118** |  |
| **119** |  |
| **121** | 1. identifying all the components of a system, establishing their interactions and assessing their dynamics – both temporal and spatial – as related to their functions 2. utilizes all types of biological information – DNA, RNA, protein, metabolites, small molecules, interactions, cells, organs, individuals, social networks and external environmental signals – integrating them |
| **122** | the fully implementation of which requires marrying basic and clinical researches through advanced systems thinking and the employment of high-throughput technologies in genomics, proteomics, nanofluidics, single-cell analysis, and computation strategies in a highly-orchestrated discipline  circularity: an essential part of the definiendum (the term “system”) is part of the definiens (in “system-thinking”) (Criterion 2) |
| **124** | using the power of computational and mathematical modeling |
| **125** | using knowledge of their molecular components   must exploit more limited data sets, arising from multiple open-ended investigations upon highly heterogeneous patient populations in conjunction with vast amounts of poorly correlated published results.Hence, systems medicine must proceed on the basis of existing, highly heterogeneous data and not on the basis of homogeneous datasets arising from specifically targeted investigations. |
| **130** | companion moleculardiagnostics for personalized therapy  the mounting influx of global quantitative data from both wellness and diseases,  which requires new strategies, both scientific and organizational  too vague: the terms „new scientific strategies“ and „new organizational strategies“ are very general and broad terms without a clear meaning (criterion 5) |
| **131** | by determining the links between genotypes, phenotypes and environmental factors (e.g. diet and exposure to toxins)  by analysing its different constituents  too vague: it is unclear what the „different constituents“ consist in and what they are constituting. (criterion 5) |
| **134** | emphasizes the role of systems biology in medical/clinical applications  With the advent of new technologies, the “omics” explosion (i.e., next generation sequencing) and the induced changes from data-poor to data-rich applications (for instance related to high-content imaging, physiology, and structural biology) have established the necessity of a systems approach (Noble, 2008)  circularity: an essential part of the definiendum (the term “system”) is part of the definiens (in “systems approach”) (Criterion 2)  Systems medicine represents a mosaic of distinct and interconnected micro-systems  originated by a variety of information sources and consequently characterized. |
| **141** | leverages complex computational tools and high-dimensional data  too vague: the exact meaning of the whole sentence, is unclear, due in particular to the term “leverages”, (criterion 5)  the effective use of petabytes of data, which necessitates the development of both new types of tools and a new type of physician—one with a grasp of modern computational sciences, “omics” technologies, and a systems approach to the practice of medicine  circularity: an essential part of the definiendum (the term “systems”) is part of the definiens (in “systems approach”) (Criterion 2)  systems biology |
| **142** |  |
| **143** | This proposed holistic strategy involves comprehensive patient-centered integrated care and multi-scale, multi-modal and multi-level systems approaches  Rather than studying each disease individually, it will take into account their intertwined gene-environment, socio-economic interactions and co-morbidities that lead to individual-specific complex phenotypes.  based on a robust and extensive knowledge management infrastructure that contains individual patient information.  too vague: it is unclear what the attributes “robust” und “extensive” might concretely mean in that context (criterion 5)  It will be supported by strategic partnerships involving all stakeholders, including general practitioners associated with patient-centered care.  too vague: the term “strategic partnerships” is unclear and is not sufficiently specified through the reference to one party included.(Criterion 5)  This systems medicine strategy, which will take a holistic approach to disease,  It uses the power of computational and mathematical modeling].  takes a holistic view of health and disease  through integrated care using multidisciplinary and teamwork approaches centered in primary and community  too vague: the whole expression is very vague and broad since it uses vague and unspecified terms such as “integrated care”; the meaning of the terms “primary” and “community” remains unclear. As a whole, it is too vague (criterion 5) |
| **146** | Understanding the unique events in an individual’s life as influencing the development of illness and disease appears to be the key to what is emerging under the names of ‘personalized medicine’ and ‘systems medicine’.  Personalized medicine presupposes systems biology and complexity sciences, […]  too vague: the predicate “presupposes” belongs more to logic than concrete means. Mentioning a presupposition does not explain very much (criterion 5) |
| **148** | Systems biology and medicine focuses on deciphering mechanisms at multiple levels, reconstructing networks in cells, tissues and organs, measuring and predicting phenotypes, building quantitative models that describe and simulate normal and pathological physiological functions, and then testing the validity of these models and predictions experimentally.  Not coherent: Systems Medicine is understood as *equivalent* to Systems Biology; at least no difference between the biological and the medical field is drawn (Criterion 6) |
| **150** | exploration of tumor microenvironment and of a more global approach to link individual tumors with their multiple host variables, including heritable causal mutations, environmental exposures and lifestyle, |
| **153** | the elucidation of drug targets, an important step in the search for new drugs or novel targets for existing drugs.  Incorporating multiple biological information sources is of essence |
| **157** | applicable methodology tool, systems biology. |
| **158** | Systems medicine, the translational science counterpart to basic science’s systems biology, is the interface at which these tools may be constructed.  […] systems medicine is the coupling of systems science with medical treatment decision-making (Auffray et al. 2009). |
| **160** | systems medicine approaches focus on the dynamic interactions among multiple factors that affect complex diseases, such as diabetes, coronary artery disease and cancers1. The increasing availability of powerful high-throughput technologies, computational tools and integrated knowledge bases, has made it possible to establish new links between genes, biologic functions and human diseases, providing the hallmarks of systems medicine, including signatures of pathology biology, and links to clinical research and drug discovery.  Holistic systems biology methodologies  through the construction of integrated biomolecular networks. |
| **162** | The knowledge of network dynamics through in vitro experimental perturbation and modeling allows us to determine the state of the networks, to identify molecular correlates, and.  The transformation in biology through systems biology  not coherent: if it is all about biology, there is no coherent relation to (Systems) medicine (Criterion 6)  The central premise of systems medicine is that clinically detectable molecular fingerprints resulting from disease-perturbed biological networks will be used to detect and stratify various pathological conditions. Disease associated molecular fingerprints will eventually be used to group individuals into sub-populations based on variations in genetic makeup of the population that affects disease progression. The key to this revolution lies in harnessing the power of network models of core biological processes learned through systems biology methods, combined with vast amounts of diverse molecular information generated from patient samples.  depends on our ability to: 1) precisely infer network state from the results of assessing the levels  of a panel of informative, diagnostic biomarkers in the blood, and 2) specifically manipulate a network to avoid or revert the pathology.  the application of our understanding of the integrated dynamical responses of various molecular networks that determine the critical states of the body.  The therapeutic component of systems medicine then, in which we infer network states from biomarker measurements and intervene |
| **164** | the application of systems biology |
| **165** | incorporates the complex biochemical, physiological, and environmental interactions that sustain living organisms.  incorporates interactions between all components of health and disease.  A key feature of systems medicine is that existing networks, through dynamic (time-dependent) interactions, manifest “emergent properties” that define the whole and that these properties are not simply the sum of the features of its component parts. |
| **170** | by integrating all levels of quantitative functional, structural, and morphological information into a coherent model.  It investigates the physiological network of diseases from gene to organ systems |
| **171** | via an integrative approach that includes clinical examinations, experimental modeling and in-silico simulation.  by integrating all levels of quantitative functional, structural and morphological information into a coherent model. |
| **175** | Systems medicine is an emerging concept that acknowledges the complexity of a multitude of non-linear interactions among molecular and physiological variables.  Under this new paradigm, rather than a collection of symptoms, diseases are seen as the product of deviations from a robust steady state compatible with life.  the incorporation of mathematics and physics to the more classical arsenal of physiology and molecular biology with which physicians are trained today. |
