# Supplementary material for: Applying systems biology to biomedical research and health care: a précising definition of systems medicine
Source: BMC Health Serv Res. 2017 Nov 21;17:761. doi: 10.1186/s12913-017-2688-z (PMC5698952; doi:10.1186/s12913-017-2688-z)
Supplement: Supplementary file 15 — Inductive derived categories from the remaining means (DOCX 37 kb) [file 12913_2017_2688_MOESM15_ESM.docx]

**Inductive derived categories from the remaining means**

**Means i) Application of Systems Biology to Medical Research and Practice**

embraces this paradigm [Systems Biology]

application of systems biology to medical research and practice

applies the perspective of SB [Systems Biology] to the study of disease mechanisms

as fusion of systems biology and bioinformatics with a focus on disease and the clinic

applies the tools and concepts from systems biology

emphasizes the role of systems biology in medical/clinical applications

systems biology

applicable methodology tool, systems biology.

Systems medicine, the translational science counterpart to basic science’s systems biology, is the interface at which these tools may be constructed.

core biological processes learned through systems biology methods, combined with vast amounts of diverse molecular information generated from patient samples

the application of systems biology

combines systems biology and pathophysiological approaches to translational research,

leverages systems biology for clinical application

an implementation of Systems Biology (SB) in the Medical disciplines

amalgamates systems biology techniques with medical treatment decision-making

Systems medicine is not simply the application of systems biology in medicine; rather, it is the logical next step and necessary extension of systems biology with more emphasis on clinically relevant applications. Building on the success of systems biology, systems medicine is defined as an emerging discipline that integrates comprehensively computational modeling, ’omics data, clinical data, and environmental factors.

[…] systems medicine is the coupling of systems science with medical treatment decision-making (Auffray et al. 2009).

**Means ii) Data Integration**

combining omics with bioinformatics, as well as functional and clinical studies

representing all the available knowledge on the disease of interest

integrate a variety of data at all relevant levels of cellular organisation with clinical and patientreported disease markers

interdisciplinary approach that integrates research data and clinical practice

close integration of data generation

tools for data integration

tandardization of data

utilizes all types of biological information – DNA, RNA, protein, metabolites, small molecules, interactions, cells, organs, individuals, social networks and external environmental signals – integrating them

must exploit more limited data sets, arising from multiple open-ended investigations upon highly heterogeneous patient populations in conjunction with vast amounts of poorly correlated published results. Hence, systems medicine must proceed on the basis of existing, highly heterogeneous data and not on the basis of homogeneous datasets arising from specifically targeted investigations.

the mounting influx of global quantitative data from both wellness and diseases

by determining the links between genotypes, phenotypes and environmental factors (e.g. diet and exposure to toxins)

originated by a variety of information sources and consequently characterized.

Incorporating multiple biological information sources is of essence

has made it possible to establish new links between genes, biologic functions and human diseases, providing the hallmarks of systems medicine, including signatures of pathology biology, and links to clinical research and drug discovery

of diverse molecular information generated from patient samples

by integrating all levels of quantitative functional, structural, and morphological information into a coherent model

by integrating all levels of quantitative functional, structural and morphological information into a coherent model

with all of a patient’s medical data being computationally integrated and accessible

to functionally interpret omics and big data

incorporating a range of personalized data including genomic, epigenetic, environmental, lifestyle and medical history

integrate data and knowledge from both clinic and basic research

Systems medicine analyzes the dynamic data cloud that surrounds each patient and uses this

where information from many biological measurements is combined and analysed for complex patterns of change

omics data, clinical data, and environmental factors

with the information on the patient, mainly of molecular origin

integrates physiopathology, network biology and molecular variations

incorporating genomic information (genomic medicine) along with appropriate biological and computational tools for data interpretation

high-throughput “omics” data

data are collected from all the components of the immune system, analyzed and integrated

**Means iii) Modelling**

with a mathematical symbolism allowing generation and testing of hypotheses through computational simulation

using the power of computational and mathematical modeling

high-precision, mathematical model of variables from different genomic layers that relate to clinical outcomes such as treatment response

close integration of data generation with mathematical modeling

integrating experiments in iterative cycles with computational modeling, simulation, and theory

using the power of computational and mathematical modeling

It uses the power of computational and mathematical modeling

and modeling allows us to determine the state of the networks, to identify molecular correlates

via an integrative approach that includes clinical examinations, experimental modeling and in-silico simulation

integrating various bio-medical tools and using the power of computational and mathematical modelling

inferred models

through a shifting paradigm, starting from a cellular, toward a patient centered framework . According to this vision, the three pillars of SM are Biomedical hypotheses, experimental data, mainly achieved by Omics technologies and tailored computational, statistical and modeling tools. The three SM pillars are highly interconnected, and their balancing is crucial

To achieve these goals, precision medicine aims to develop computational models that integrate data and knowledge from both clinic and basic research

which purports to design multiscale mathematical disease models

systems medicine is defined as an emerging discipline that integrates comprehensively computational modeling

where traditional model-driven experiments are informed by data-driven models in an iterative manner

the use of network-based models of biological process combined with the information on the patient, mainly of molecular origin

by integrating all levels of quantitative functional, structural and morphological information into a coherent model

**Mean iv) Network**

network-based approach to analysis of high-throughput and routine clinical data to predict disease mechanisms to diagnoses and treatments

applying a network-level view of disease

analyzing the interactions between the different components within one organizational level (genome, transcriptome, proteome), and then between the different levels

identifying important functional and regulatory modules within these networks

by analyzing and targeting hubs—the most highly interconnected nodes—within these regulatory networks, and enzymatic activity *in metabolic networks*

through the construction of integrated biomolecular networks

The knowledge of network dynamics through in vitro experimental perturbation

The key to this revolution lies in harnessing the power of network models of core biological processes learned through systems biology methods

depends on our ability to: 1) precisely infer network state from the results of assessing the levels

of a panel of informative, diagnostic biomarkers in the blood and 2) specifically manipulate a network

the application of our understanding of the integrated dynamical responses of various molecular networks that determine the critical states of the body

A key feature of systems medicine is that existing networks, through dynamic (time-dependent) interactions

It investigates the physiological network of diseases from gene to organ systems

scrutinizing overall molecular network interactions, rather than individual molecules

implies the establishment of a connection between a molecular-centered to a patient-centered world, through an organ-centered intermediate layer

is concerned with the network of molecular interactions that define biological processes

the therapeutic component of systems medicine then, in which we infer network states from biomarker measurements and intervene

Additionally, disease states are viewed as a perturbation of these molecular networks

ultimately leading to the practical result of uncovering novel dynamic interaction networks that are critical

molecular fingerprints resulting from biological networks perturbed by the disease will be used

integrates physiopathology, network biology and molecular variations

**Means v) Bioinformatics/Computer tools /Computational Analysis**

as fusion of systems biology and bioinformatics

statistical and computational analysis of metabolic, phenotypic, and physiological data

application of computational and statistical approaches to support clinical decisions

the incorporation of mathematics and physics to the more classical arsenal of physiology and molecular biology with which physicians are trained today

incorporating genomic information (genomic medicine) along with appropriate biological and computational tools for data interpretation

This mapping (Figure 1) requires the extensive use of computational tools such as statistical, mathematical and bioinformatical techniques

The increasing availability of powerful high-throughput technologies, computational tools and integrated knowledge bases has made it possible to establish new links between genes, biologic functions and human diseases

**Means vi) Addressing Complexity**

and addresses complexity in two key ways

different specific complex factors are important in disease management and that these factors need to be incorporated in some meaningful way

identifying all the components of a system, establishing their interactions and assessing their dynamics – both temporal and spatial – as related to their functions

Systems medicine represents a mosaic of distinct and interconnected micro-systems

Rather than studying each disease individually, it will take into account their intertwined gene-environment, socio-economic interactions and co-morbidities that lead to individual-specific complex phenotypes.

systems medicine approaches focus on the dynamic interactions among multiple factors that affect complex diseases, such as diabetes, coronary artery disease and cancers

incorporates the complex biochemical, physiological, and environmental interactions that sustain living organisms

incorporates interactions between all components of health and disease

existing networks, through dynamic (time-dependent) interactions, manifest “emergent properties” that define the whole and that these properties are not simply the sum of the features of its component parts

Systems medicine is an emerging concept that acknowledges the complexity of a multitude of non-linear interactions among molecular and physiological variables. Under this new paradigm, rather than a collection of symptoms, diseases are seen as the product of deviations from a robust steady state compatible with life.

where information from many biological measurements is combined and analysed for complex patterns of change

scrutinizing overall molecular network interactions, rather than individual molecules

**Means vii) Stratification**

First, systems medicine uses molecular diagnostics to stratify patients and diseases

using knowledge of their molecular components

companion molecular diagnostics for personalized therapy

The central premise of systems medicine is that clinically detectable molecular fingerprints resulting from disease-perturbed biological networks will be used to detect and stratify various pathological conditions. Disease associated molecular fingerprints will eventually be used to group individuals into sub-populations based on variations in genetic makeup of the population that affects disease progression.

using molecular and dynamic parameters

through stratification of patients and diseases

**Means viii) Holistic Approach**

This proposed holistic strategy involves comprehensive patient-centered integrated care and multi-scale, multi-modal and multi-level systems approaches

This systems medicine strategy, which will take a holistic approach to disease

takes a holistic view of health and disease

Holistic systems biology methodologies

**Means ix) Understanding of Illnesses/Diseases**

Understanding the unique events in an individual’s life as influencing the development of illness and disease appears to be the key to what is emerging under the names of ‘personalized medicine’ and ‘systems medicine’

the elucidation of drug targets, an important step in the search for new drugs or novel targets for existing drugs

To achieve these goals, precision medicine aims to develop computational models that integrate data and knowledge from both clinic and basic research

to functionally interpret omics and big data

exploration of tumor microenvironment and of a more global approach to link individual tumors with their multiple host variables, including heritable causal mutations, environmental exposures and lifestyle,

**Means x) Experimental Validation/Experimental examination**

representing all the available knowledge on the disease of interest with a mathematical symbolism allowing generation and testing of hypotheses through computational simulation and experimental validation

integrating experiments in iterative cycles with computational modeling, simulation, and theory

The knowledge of network dynamics through in vitro experimental perturbation

via an integrative approach that includes clinical examinations, experimental modeling and in-silico simulation

through a shifting paradigm, starting from a cellular, toward a patient centered framework . According to this vision, the three pillars of SM are Biomedical hypotheses, experimental data

where traditional model-driven experiments are informed by data-driven models in an iterative manner
